# Development and validation of an integrative pan-solid tumor predictor of PD-1/PD-L1 blockade benefit

**DOI:** 10.1038/s43856-023-00243-7

**Published:** 2023-02-07

**Authors:** Scott A. Tomlins, Nickolay A. Khazanov, Benjamin J. Bulen, Daniel H. Hovelson, Melissa J. Shreve, Laura E. Lamb, Marc R. Matrana, Mark E. Burkard, Eddy Shih-Hsin Yang, William Jeffery Edenfield, E. Claire Dees, Adedayo A. Onitilo, Michael Thompson, Gary L. Buchschacher, Alan M. Miller, Alexander Menter, Benjamin Parsons, Timothy Wassenaar, Leon C. Hwang, J. Marie Suga, Robert Siegel, William Irvin, Suresh Nair, Jennifer N. Slim, Jamal Misleh, Jamil Khatri, Gregory Masters, Sachdev Thomas, Malek Safa, Daniel M. Anderson, Kat Kwiatkowski, Khalis Mitchell, Tina Hu-Seliger, Stephanie Drewery, Andrew Fischer, Komal Plouffe, Eric Czuprenski, Jennifer Hipp, Travis Reeder, Hana Vakil, D. Bryan Johnson, Daniel R. Rhodes

**Affiliations:** 1 Strata Oncology, Ann Arbor, MI USA; 2 Ochsner Cancer Institute, New Orleans, LA USA; 3 University of Wisconsin Carbone Cancer Center, Madison, WI USA; 4 O’Neal Comprehensive Cancer Center, University of Alabama at Birmingham School of Medicine, Birmingham, AL USA; 5 Prisma Health Greenville Memorial Hospital, Greenville, SC USA; 6 UNC Lineberger Comprehensive Cancer Center, Chapel Hill, NC USA; 7 Cancer Care and Research Center, Marshfield Clinic Research Institute, Marshfield, WI USA; 8 Aurora Cancer Care, Advocate Aurora Health, Milwaukee, WI USA; 9 Kaiser Permanente Southern California, Los Angeles, CA USA; 10 SCL Health-CO, Broomfield, CO USA; 11 Kaiser Permanente Colorado, Lone Tree, CO USA; 12 Gundersen Health System, La Crosse, WI USA; 13 UW Health Cancer Center at ProHealth Care, Waukesha, WI USA; 14 Kaiser Permanente of the Mid-Atlantic States, Rockville, MD USA; 15 Kaiser Permanente Northern California, Vallejo, CA USA; 16 Bon Secours St. Francis Cancer Center, Greenville, SC USA; 17 Bon Secours Cancer Institute, Midlothian, VA USA; 18 Lehigh Valley Topper Cancer Institute, Allentown, PA USA; 19 MultiCare Regional Cancer Center, Tacoma, WA USA; 20 The US Oncology Network, Newark, DE USA; 21 ChristianaCare Oncology Hematology, Newark, DE USA; 22 Medical Oncology Hematology Consultants, Helen F Graham Cancer Center and Research Institute,, Newark, DE USA; 23 Kaiser Permanente - Northern California, Oakland, CA USA; 24 Kettering Health, Kettering, OH USA; 25 Metro-Minnesota Community Oncology Research Consortium, St. Louis Park, MN USA; 26 Present Address: Tempus Labs, Chicago, IL USA; 27 Present Address: Translational Drug Development, Scottsdale, USA

**Keywords:** Predictive markers, Tumour biomarkers

## Abstract

**Background:**

Anti-PD-1 and PD-L1 (collectively PD-[L]1) therapies are approved for many advanced solid tumors. Biomarkers beyond PD-L1 immunohistochemistry, microsatellite instability, and tumor mutation burden (TMB) may improve benefit prediction.

**Methods:**

Using treatment data and genomic and transcriptomic tumor tissue profiling from an observational trial (NCT03061305), we developed Immunotherapy Response Score (IRS), a pan-tumor predictive model of PD-(L)1 benefit. IRS real-world progression free survival (rwPFS) and overall survival (OS) prediction was validated in an independent cohort of trial patients.

**Results:**

Here, by Cox modeling, we develop IRS—which combines TMB with *CD274*, *PDCD1*, *ADAM12* and *TOP2A* quantitative expression—to predict pembrolizumab rwPFS (648 patients; 26 tumor types; IRS-High or -Low groups). In the 248 patient validation cohort (248 patients; 24 tumor types; non-pembrolizumab PD-[L]1 monotherapy treatment), median rwPFS and OS are significantly longer in IRS-High vs. IRS-Low patients (rwPFS adjusted hazard ratio [aHR] 0.52, p = 0.003; OS aHR 0.49, p = 0.005); TMB alone does not significantly predict PD-(L)1 rwPFS nor OS. In 146 patients treated with systemic therapy prior to pembrolizumab monotherapy, pembrolizumab rwPFS is only significantly longer than immediately preceding therapy rwPFS in IRS-High patients (interaction test p = 0.001). In propensity matched lung cancer patients treated with first-line pembrolizumab monotherapy or pembrolizumab+chemotherapy, monotherapy rwPFS is significantly shorter in IRS-Low patients, but is not significantly different in IRS-High patients. Across 24,463 molecularly-evaluable trial patients, 7.6% of patients outside of monotherapy PD-(L)1 approved tumor types are IRS-High/TMB-Low.

**Conclusions:**

The validated, predictive, pan-tumor IRS model can expand PD-(L)1 monotherapy benefit outside currently approved indications.

## Plain language summary

Therapies activating the immune system (checkpoint inhibitors) have revolutionized the treatment of patients with advanced cancer, however new molecular tests may better identify patients who could benefit. Using treatment data and clinical molecular test results, we report the development and validation of Immunotherapy Response Score (IRS) to predict checkpoint inhibitor benefit. Across patients with more than 20 advanced cancer types, IRS better predicted checkpoint inhibitor benefit than currently available tests. Data from >20,000 patients showed that IRS identifies ~8% of patients with advanced cancer who may dramatically benefit from checkpoint inhibitors but would not receive them today based on currently available tests. Our approach may help clinicians to decide which patients should receive checkpoint inhibitors to treat their disease.

## Abstract (web summary)

Tomlins et al. develop an Immunotherapy Response Score (IRS) to predict clinical benefit from checkpoint inhibition across solid tumors. IRS integrates TMB and gene expression, is evaluable from FFPE material, and is predictive for real-world progression-free survival and overall survival in patients receiving PD-1/PD-L1 blockade therapy.

## Introduction

Anti-PD-1 and anti-PD-L1 (PD-[L]1) monoclonal antibodies, known as checkpoint inhibitors (CPIs), have transformed cancer care, and are approved for use in multiple tumor types and pan tumor indications (microsatellite instability high/mismatch repair deficient [MSI-H/dMMR] and tumor mutation burden [TMB] ≥ 10 mutations/megabase [Muts/Mb])^1–3^. Improved biomarkers capable of predicting anti-PD-(L)1 benefit have the potential to expand CPIs to additional patient populations outside of currently approved indications, and to focus their application more effectively on likely responsive patients when alternative therapies exist. PD-L1 immunohistochemistry (IHC) is required for treatment in many tumor types and serves as a companion diagnostic biomarker; although antibodies, staining platforms, PD-L1 expressing cells included in scoring algorithms, and cutoffs vary across tumor types^4–14^. In addition, high TMB predicts CPI response across multiple tumor types, although TMB determination approaches vary across studies and tests, only a fraction of TMB high (TMB-H) patients benefit, and a single TMB cutoff may not be optimum across tumor types or CPIs^15–24^. For example, in the KEYNOTE-158 study of 9 tumor types leading to pan-solid tumor approval of second-line pembrolizumab (anti-PD-1) in patients with TMB ≥ 10 Muts/Mb by the FoundationOne companion diagnostic (CDx) comprehensive genomic profiling (CGP) device, objective responses were observed in 37%, 13%, and 6% of patients with TMB ≥ 13 Muts/Mb, ≥10 and <13 Muts/Mb, and <10 Muts/Mb, respectively^25,26^.

Additionally, although only pembrolizumab is approved for patients with high TMB, numerous retrospective and prospective analyses support the clinical utility of high TMB by comprehensive genomic profiling (CGP) for predicting durable responses to other anti-PD-(L)1 monotherapies, including both other PD-1 (e.g. nivolumab) and PD-L1 (e.g. atezolizumab) monoclonal antibodies^27–32^. Notably, in prospective basket studies of patients with ≥2nd line solid tumors having high TMB by FoundationOne CDx treated with nivolumab or atezolizumab, ORRs of 28% (n = 10/36) and 19% (n = 17/90), respectively, were observed in patients with TMB ≥ 10 Mut/Mb, with increased ORRs of 47% (*n* = 8/17) and 38% (*n* = 16/42), respectively, in patients with TMB ≥ 16 Mut/Mb^31,32^. In addition to potentially identifying patients outside of current indications who may benefit from PD-(L)1 monotherapy, given the increasing number of approved PD-(L)1 combination therapy regimens and the thousands of ongoing combination trials, biomarkers enabling the identification of PD-(L)1 monotherapy benefit is of particular importance in tumor types where only combination therapy regimens are approved (or monotherapy is only approved in later lines) as combination regimens have increased clinical and financial toxicity and a recent meta-analysis demonstrating essentially no evidence for additive or synergistic benefit between PD-(L)1 therapies and other agents in approved combination regimens^33^.

Numerous translational research studies have demonstrated that PD-L1 expression, TMB (with clonal TMB showing increased predictive ability vs. TMB methods including all somatic mutations), and other immune related gene expression markers focusing on the tumor microenvironment (TME) are independent predictors of response^15,34–47^. For example, in bladder cancer, multiple studies have demonstrated the potential for PD-L1 by IHC, TMB, and T-cell-inflamed gene expression to predict PD-(L)1 therapy benefit, whether alone or in combination with chemotherapy, with an only increasing need to maximize PD-(L)1 benefit given the number of other approved agents in different therapy classes (chemotherapy, antibody drug conjugates and small molecule inhibitors) that must sequenced^48–53^.

Importantly, however, a single, integrative, clinically applicable and validated test for treatment selection across solid tumors is lacking. Herein, leveraging PD-(L)1 therapy treatment data and CGP plus quantitative transcriptomic profiling (CGP + qTP) data from the Strata Trial (NCT03061305)—an observational clinical trial evaluating the impact of molecular profiling on patients with advanced solid tumors—we report the development and validation of an integrated Immunotherapy Response Score (IRS) that predicts pan-solid tumor PD-(L)1 benefit by both real-world progression free survival (rwPFS) and overall survival (OS) by an analytically and clinically validated CGP + qTP laboratory developed test (LDT) applicable to minute formalin-fixed paraffin-embedded (FFPE) tissue specimens.

## Methods

### Cohort

The Strata Trial (NCT03061305), is an observational clinical trial evaluating the impact of molecular profiling on patients with advanced solid tumors. It has been centrally reviewed and approved by Advarra Institutional Review Board (IRB; IRB Pro00019183) prior to study start; this study was performed in accordance with the Declaration of Helsinki and we have complied with all relevant ethical regulations. All patients provided written informed consent for Strata Trial participation, except at institutions where a waiver of informed consent was granted by the central and/or local IRB (if not ceded to central review) and applied due to minimal risk of using surplus tissue specimens (Supplementary Data 1). At enrolling health care systems, all adult patients with locally advanced (stage III), unresectable or metastatic (stage IV) solid tumors and available FFPE tumor tissue were eligible; the protocol also allowed enrollment of patients with rare early-stage tumors.

The Strata Clinical Molecular Database (SCMD) contains deidentified subject, molecular profiling, treatment, and survival data for all enrolled NCT03061305 participants. Prior antineoplastic therapy, including start and stop dates, were collected for trial participants at the time of study entry. Antineoplastic therapy data and survival status were prospectively collected for up to 3 years from the time of enrollment and/or informed consent. First, a case series analysis was performed herein focusing on the development of an integrative CGP + qTP based PD-(L)1 benefit predictor, an exploratory aim of the trial. Post-hoc power analysis was not performed to determine the sample size of this discovery cohort. A power analysis was then performed to determine the cohort size needed for an independent validation cohort as described below. Patients in the SCMD tested by a version of StrataNGS assessing TMB (see Biomarker Data below) with parallel gene expression testing data completed between 25 January 2017 to 12 July 2022 were eligible for analysis with a data cutoff of 12 July 2022; for the discovery cohort, only patients tested through 04 May 2021 were eligible and the data cutoff date was the same as the overall cohort. General validity analyses of the SCMD are described in the Supplementary Methods.

For both the discovery and validation cohorts, common inclusion criteria were: valid TMB measurements from StrataNGS testing (including meeting the overall 20% tumor content requirement), valid immune gene expression quantification from an investigative multiplex PCR based transcriptomic profiling test, and documented treatment with at least one antineoplastic agent. For the discovery cohort, additional inclusion and exclusion criteria included: (1) treatment with a pembrolizumab containing systemic line of therapy, (2) the tested tissue specimen was collected prior to the systemic pembrolizumab line start date, and (3) the patient had no prior anti-PD-(L)1 or CTLA4 blockade therapy prior to the pembrolizumab line start date. For the validation cohort, additional inclusion and exclusion criteria included: (1) treatment with systemic non-pembrolizumab anti-PD-(L)1 monotherapy, (2) the tested tissue specimen was collected prior to the PD-(L)1 therapy start date, (3) had no prior anti-PD-(L)1 or CTLA4 blockade therapy prior to the non-pembrolizumab PD-(L)1 line start date, and (4) patients were not in the discovery cohort. Additional inclusion/exclusion criteria for other analyses are described below and in the overall study diagram (Fig. S1). Except in the analysis specifically assessing IRS performance in samples collected after PD-(L)1 therapy, patients with samples collected after the start date of the analyzed therapy line were excluded from all analyses.

Source data verification in the Strata Trial was performed for high-risk data fields such as demographics and treatment history per an approved Trial Monitoring Plan. Data completeness, consistency, and quality assurance checks were performed across the Strata electronic data capture (EDC) system per an approved Data Management Plan; 100% source data verification was performed for the discovery cohort. Additional details on the Strata Trial experience and Strata molecular profiling have been described^54–56^.

### Real-world treatment data

Patient treatment history from electronic health records (EHRs) or manual updating was standardized to enable derivation of real-world progression free survival (rwPFS) by time to next therapy (TTNT) and OS. All medications were classified into anti-neoplastic or non-antineoplastic treatments, and all anti-neoplastic treatments were further subclassified (e.g chemotherapy, immune-oncology [IO; PD-(L)1 or CTLA4], oncogene TKI, hormonal, etc); non-antineoplastic treatments were excluded from further consideration. Line of therapy assignment was performed in two stages: first, single-dose treatments with consecutive doses administered within 90 days were combined into a course of treatment with a single start and end date; next, non-overlapping lines of treatment were inferred by considering each course of medication sequentially by start date. Subsequent treatment courses that began more than 30 days after the start of a given line of treatment, or whose duration of overlap with the line was less than 50%, were considered to establish a new line of treatment. Any treatment line with more than one anti-neoplastic therapy administered during the line was considered combination therapy. First line chemo and/or hormonal therapies which concluded 180 days or more prior to the start of subsequent therapy were considered as adjuvant.

To determine rwPFS, an effective end date was defined for each course of treatment as either (a) date of last record if treatment is ongoing (censored), (b) date of death (event), (c) the start date of the subsequent therapy line (event), or (d) the latest available end date (censored if no subsequent line of therapy or death). rwPFS was calculated as the difference, in months, between the start date and effective end date of the treatment line. OS was calculated as the difference, in months, between the start date of the treatment line and date of death (or censoring).

### Biomarker data

Multiplex PCR-based comprehensive genomic profiling (PCR-CGP), including TMB assessment, was performed on FFPE solid tumor tissue using StrataNGS (Strata Oncology, Ann Arbor, MI). The current version of StrataNGS is a 437 gene laboratory-developed test (LDT) for FFPE tumor tissue samples performed on co-isolated DNA and RNA, which has been validated on over 1,900 FFPE tumor samples, and is covered for Medicare beneficiaries^55^. While earlier StrataNGS versions were also used during the study period, all had similar performance for the TMB assessment (and MSI) used herein^56^. In parallel, immune gene expression was determined by analytically and clinically validated multiplex PCR-based qTP via an investigational/supplementary test performed on the same co-isolated RNA as described^54^; different versions of this quantitative transcriptomic profiling test have been run in parallel with StrataNGS (assessing 26, 46, and currently 103 expression targets), with panel specific scaling validated by concordance analyses performed as needed. One or more exon-spanning PCR amplicons were selected for each target gene and multiple housekeeping genes (see Supplementary Methods) were included, with three pan-cancer stable housekeeping genes used for clinical testing. qTP was performed using Ampliseq after reverse transcription followed by Ion Torrent-based next-generation sequencing. Expression target transcripts were measured in normalized reads per million (nRPM), whereby raw expression target read counts were normalized by a factor that results in the median housekeeping gene expression value matching the same gene’s standard reads per million in a reference FFPE normal cell line sample (GM24149) run in parallel with all clinically tested samples^54^. Relevant components of the analytical and clinical validation of the current version of the integrated CGP + qTP LDT that includes the IRS model are described in the Supplementary Methods.

### Statistical analysis

Unadjusted rwPFS and overall-survival (OS) across groups and treatments were visualized using the Kaplan Meier method. Adjusted rwPFS and OS analyses were performed to compare group outcomes (by adjusted hazard ratios and two-sided *p*-values) using Cox proportional hazard models unless otherwise specified. Covariate adjustments shared between all models include age and gender. Repeated measures were accounted for in settings where participants had multiple records (e.g., prior treatment then pembrolizumab monotherapy). Analysis dependent covariates, as appropriate, included IRS group, tumor type (most prevalent in cohort vs. all other types), systemic therapy line number, TMB status (High vs. Low), therapy type (monotherapy or combination), PD-(L)1 therapy type (PD-1 or PD-L1 therapy), *CDKN2A* status (wild type or deep deletion) and tumor content (continuous). The indicated analysis used MSKCC definition of TMB sensitive tumor types (MSI-H, *POLE*^mutant^, non-small cell lung cancer [NSCLC], head and neck cancer, or melanoma as TMB sensitive; all other samples as TMB insensitive)^57^ instead of most prevalent tumor type vs. all others. Performance status (or surrogates) were not available from data collected as part of the Strata Trial. Proportional hazard assumptions were checked for each model and cohort of interest using Schoenfeld residuals. Unstratified analysis results are presented throughout, as stratifying analyses to preserve proportional hazards produced similar covariate effect sizes where the assumption was not met (discovery cohort); all monotherapy discovery cohort analyses and validation cohort analyses met proportional hazard assumptions. Where specified, the two-sided log-rank test was used to test rwPFS and OS curve differences (Benjamini Hochberg adjusted as appropriate).

For the predictive analysis using the internal comparator cohort considering rwPFS on the immediately preceding systemic therapy vs. subsequent pembrolizumab monotherapy, adjusted Cox proportional hazards models were utilized to examine the interaction between pembrolizumab vs. prior chemotherapy rwPFS within the same patient and IRS status (IRS-high vs. low). The likelihood ratio test for interaction compared the reduced model, which excluded the IRS by treatment interaction, with the competing full model, which included the IRS by treatment interaction.

To determine the performance of IRS in a setting where both PD-(L)1 monotherapy and combination therapy are used in the same line, we limited the discovery cohort to the subset of patients with NSCLC treated with first line pembrolizumab monotherapy or pembrolizumab + chemotherapy combination therapy. As PD-L1 IHC status and performance status were not available and known confounders driving this treatment decision, we performed nearest neighbor propensity score matching (with a 0.25 standard deviation caliper applied)^58^ using age, gender, TMB, IRS, and the normalized *PD-L1* expression component of the IRS biomarker (see Supplementary Results for validation of this biomarker vs. IHC in a separate cohort). All patients in the combination therapy cohort who could not be matched to within 0.25 * standard deviation a monotherapy patient’s propensity score were dropped. Confirmation that the final matched monotherapy and combination therapy did not significantly differ (two tailed *t*-tests for continuous variables and two-tailed Fisher’s exact test for categorical variables; both at *p* < 0.05 as significant) was performed. Kaplan Meier analysis was used to visualize monotherapy vs. combination therapy rwPFS in the separate IRS-H and IRS-L populations, using a two-sided log-rank test to compare therapy group outcomes.

The correlation between rwPFS and OS was calculated using Spearman’s *p* among patients with both a documented death event and at least two lines of therapy. Throughout this study, TMB-H was defined as ≥10 Muts/Mb by StrataNGS, given the previous validation of TMB by StrataNGS and high concordance with TMB estimates from FoundationOne tissue testing (see Supplementary Methods)^55^. All statistical analyses were performed in R (v. 4), and SAS (v. 9.4). For all cohort analyses, two-sided *p*-values < 0.05 were considered statistically significant.

### Immunotherapy response score (IRS) model development and validation

The association of TMB and 23 candidate immune and proliferation gene expression biomarkers with pembrolizumab rwPFS was determined using Cox proportional hazards regression in the 648-patient pembrolizumab (both monotherapy and combination therapy) discovery cohort. TMB measurements were log_2_-transformed and gene expression measurements were log_2_-transformed and median-centered per laboratory workflow prior to analysis. Feature selection was performed via Lasso-penalized Cox proportional hazards regression in this 648-patient discovery cohort, with the Lasso penalty term chosen as the value which maximized the concordance index via 5-fold cross validation. Model coefficients for the five features with non-0 coefficients in the Lasso model were finalized via standard Cox regression. Individual patient IRS were derived from the Cox model as:

IRS = 0.273758 * TMB + 0.112641 * *PD-1* + 0.061904 * *PD-L1* - 0.077011 * *TOP2A* - 0.057991 * *ADAM12*

We assigned patients to one of two IRS groups to compare patient outcomes (i.e., Low (L) < 0.873569 and High (H) ≥ 0.873569; more likely to benefit) based on balancing minimization of the hazard ratio for IRS-H vs. IRS-L with maximization of the IRS-H monotherapy population.

After locking the IRS model (and -H vs. -L threshold), a power analysis was performed to determine the size of an appropriate independent validation cohort. In the overall discovery cohort, 46% patients were IRS-H, and we observed an adjusted hazard ratio for IRS-H vs. IRS-L rwPFS of 0.49 (47% event rate); therefore, assuming an IRS-H to IRS-L ratio of 1:1 and a 50% event rate, a validation cohort of 180 patients would have 90% power to detect a similar (0.5) hazard ratio. We then identified all (*n* = 248) patients in the SCMD meeting the above-described validation cohort inclusion/exclusion criteria (the same as the discovery cohort except only including any non-pembrolizumab PD-(L)1 monotherapy treatment); the locked IRS model (and -H vs. -L threshold) was then applied to these subjects.

### Reporting summary

Further information on research design is available in the Nature Portfolio Reporting Summary linked to this article.

## Results

### Clinical molecular data

The Strata Trial (NCT03061305) is an observational clinical trial evaluating the impact of tumor molecular profiling for patients with advanced solid tumors. De-identified demographic, clinical and molecular data from patients in the Strata Trial is maintained in the Strata Clinical Molecular Database (SCMD). With a data-cutoff of 12 July 2022, the SCMD contains clinical and molecular data from a total of 57,648 unique patients with advanced solid tumors (from 47 tumor types) from 59 United States health care systems who had routine FFPE tumor tissue molecularly profiled by the StrataNGS CGP test^55,56^, with 9899 Strata Trial patients from 30 United States health care systems (from 43 tumor types) having treatment data from at least one systemic antineoplastic agent (Figure S1, and Supplementary Data 2 and 3).

For all Strata Trial patients with treatment data in the SCMD, antineoplastic treatment start and stop dates (for all prior therapies and up to 3 years after Strata trial enrollment) were obtained from automated electronic health record queries or manual entry; data was updated regularly by submitting institutions, and date of death was obtained similarly. Time to next therapy (TTNT) as a measure of real-world progression free survival (rwPFS) was determined directly from treatment start and stop dates for each line of therapy accounting for adjuvant/systemic therapy, monotherapy/combination therapy, potential overlap of treatment start/stop dates, repeating lines of therapy (whether monotherapy in combination) given the variance in real world treatment patterns (Figure S2). Clinical results from the overall 9,899 patient cohort, including shorter rwPFS with subsequent therapy lines (as expected), as well as analyses supporting the general validity of the SCMD, are shown in Figure S3 &4 and described in the Supplementary Results.

### Biomarkers of anti-PD-1/PD-L1 blockade benefit analysis

**Fig. 1:**
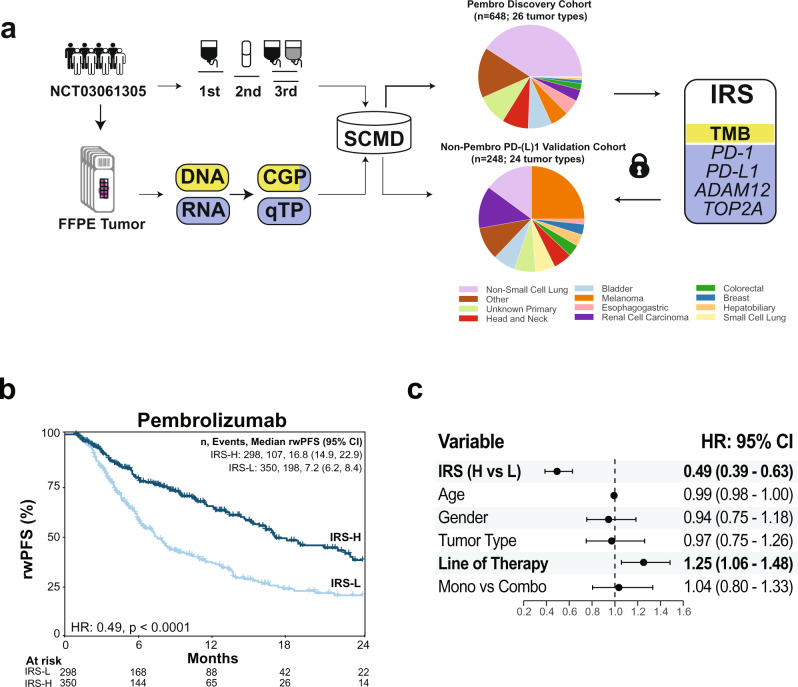
Development of an integrative immunotherapy response score (IRS) model to stratify PD-(L)1 therapy benefit in patients with advanced solid tumors. **a** Real-world treatment and molecular profiling data from formalin fixed paraffin embedded (FFPE) tumor tissue from patients enrolled in the StrataTrial (NCT03061305) are collected in the Strata Clinical Molecular Database (SCMD). Molecular data from both DNA (yellow) and RNA (blue) include both comprehensive genomic profiling (CGP) with both DNA and RNA components, and in-parallel quantitative transcriptional profiling (qTP) comprised of RNA from analytically and clinically validated tests. To develop an integrative predictor of PD-(L)1 therapy benefit, we identified a cohort of 648 patients (from 26 tumor types) with available molecular information who were treated with a pembrolizumab (pembro; PD-1) containing systemic therapy line of treatment. Lasso-penalized Cox proportional hazards modeling with five-cross validation was used to develop the IRS model for predicting real world progression free survival (rwPFS; by time to next therapy), which includes tumor mutation burden (TMB; from CGP) and expression of *PD-1*, *PD-L1*, *ADAM12* and *TOP2A* (from qTP). The locked IRS model and threshold to assign patients to IRS-Low [L] or IRS-High [H; increased benefit] was then applied to an independent validation cohort of 248 patients (from 24 tumor types) treated with non-pembrolizumab PD-[L]1systemic monotherapy. Pie charts for the development and validation cohorts show tumor type distributions for the 11 most common tumor types and other tumor types. **b** IRS stratifies pembrolizumab rwPFS in the development cohort. Pembrolizumab rwPFS in the development cohort stratified by IRS groups is shown by Kaplan Meier analysis with the adjusted hazard ratio (HR) and *p* value (adjusted by variables shown in (**c**) for IRS-H vs. IRS-L. The number (*n*) of patients, events, and median rwPFS (with 95% confidence intervals [CI]) for each group are shown. **c** IRS is robust to potential confounders in the development cohort. Forest plot of variables included in the adjusted Cox proportional hazards model used to evaluate the ability of IRS to stratify pembrolizumab rwPFS. Adjusted hazard ratios with 95% CIs are shown for each variable with statistically significant variables bolded. *n* = 648 patients (from 26 tumor types).

To develop an integrative, CGP + qTP based tumor-agnostic PD-(L)1 blockade predictive biomarker, we first limited results to the 648 of 9899 (6.5%) patients in the SCMD who met all of the inclusion/exclusion criteria (see Methods) including: valid TMB measurements from StrataNGS testing (including meeting the overall 20% tumor content requirement), valid immune gene expression quantification from an investigative multiplex PCR based qTP test, and with a pembrolizumab containing systemic line of therapy (Fig. S1). As shown in Fig. 1a, this discovery cohort was comprised of patients with 26 tumor types, with NSCLC accounting for 265 (40.9%); tumor types and demographics are provided in Supplementary Data 2 and 3. rwPFS was inferred for each patient as the time from starting the pembrolizumab containing therapy line to the time of stopping that line and starting a new therapy line or death; both rwPFS and OS were used for studying treatment outcome based on comparisons of these endpoints (Supplementary Results and Fig. S5). The clinical validity of TMB status by StrataNGS was confirmed as shown in Fig. S6 and described in the Supplementary Results.

To identify potential expression-based biomarkers of PD-(L) therapy benefit beyond TMB, we first assessed the association of pembrolizumab rwPFS with 23 candidate immune and proliferation gene expression biomarkers (from 21 genes; two amplicons targeting separate exon-exon junctions of *PDCD1* [*PD-1*] and *CD274* [*PD-L1*] were included) assessed across clinical RNA tests run in parallel with the StrataNGS CGP test (which generates TMB), with univariate predictor results shown in Table S1. Data on housekeeping gene selection, correlation of independent *PD-1* and *PD-L1* amplicons, correlation of tumor-type expression profiles for candidate gene expression biomarkers between SCMD and The Cancer Genome Atlas (TCGA) profiled tumors, and analytical and clinical validation of the qTP component of the CGP + qTP test (including qRT-PCR and clinical IHC data from >1,000 total FFPE tumors) is described in the Supplementary Methods, Supplementary Results, Table S2, and Figs. S7 and S8.

### Integrative immunotherapy response score (IRS) to predict PD-(L)1 blockade benefit

To develop an integrative model predictive of PD-(L)1 therapy benefit, we performed Lasso-penalized Cox proportional hazards regression with five-fold cross-validation in this 648 patient discovery cohort, with the highest concordance index obtained using a five-term model that included TMB, *PD-1, PD-L1, ADAM12*, and *TOP2A* (Fig. S9), with increasing TMB, *PD-1* and *PD-L1* associated with longer pembrolizumab rwPFS, while increasing *ADAM12* and *TOP2A* were associated with shorter pembrolizumab rwPFS. As the same feature set was also obtained via exhaustive combinatorial search of all five-term models by standard Cox proportional hazards regression, the five term Cox proportional hazards model was used to generate the final integrative model (multivariate analysis on the final five variable set is shown in Table S1). As shown in Table S3, across 24,463 Strata Trial samples in the SCMD with informative TMB and gene expression (regardless of treatment data availability), TMB was minimally correlated with all final model gene expression biomarkers (Spearman *ρ* = 0.032 [*ADAM12*] to 0.211 [*TOP2A]*), while correlation of individual gene expression biomarkers ranged from *ρ* = 0.033 (*PD-1* vs. *TOP2A*) to *ρ* = 0.571 (*PD-1* vs. *PD-L1*).

To evaluate the potential of the multivariate model to predict PD-1/PD-L1 blockade treatment outcome, we derived individual Immunotherapy Response Scores (IRS) from the final five variable model, assigned the 648 patients to either IRS-High [-H; *n* = 298 (46.0%); associated with greater benefit of PD-1/PD-L1 blockade] or IRS-Low groups (threshold set by balancing maximization of IRS-H group size vs. minimization of the unadjusted rwPFS IRS hazard ratio), and compared group outcomes by Kaplan Meier analysis and Cox proportional hazards modeling after adjusting for age, gender, most frequent tumor type (NSCLC) vs. others, line type (monotherapy/combination therapy) and line of systemic therapy. As shown in Fig. 1b, c, IRS-H patients had significantly longer pembrolizumab rwPFS (IRS-H vs. IRS-L median rwPFS 16.8 [95% CI: 14.9–22.9] vs. 7.2 [95% CI: 6.2–8.4] months, adjusted hazard ratio 0.49 [95% CI: 0.39–0.63], *p* < 0.0001) and OS (IRS-H vs. IRS-L median OS Not Reached [95% CI: 29.9–NA] vs. 17.1 [95% CI 13.4-22.8] months, adjusted hazard ratio 0.53 [95% CI: 0.40–0.70], *p* < 0.0001; Fig. S10a, b). IRS-H also showed significant rwPFS and OS benefit when using restricted mean survival time analysis (prespecified periods of 24 months and 36 months, respectively), both in an unadjusted analysis (IRS-H vs. IRS-L average event free rwPFS 15.70 [95% CI: 14.53–16.88] vs.10.63 [95% CI: 9.61–11.65]; OS 25.50 [95% CI: 23.61–27.39] vs. 19.24 [95% CI: 17.48–21.00] and when adjusting for the same CPH model covariates above (rwPFS IRS-H vs. IRS-L 4.80 [95% CI: 3.20–6.41], *p* < 0.0001; OS IRS-H vs. IRS-L 6.00 [95% CI: 3.37–8.63], *p* < 0.0001) Table S4).

**Fig. 2:**
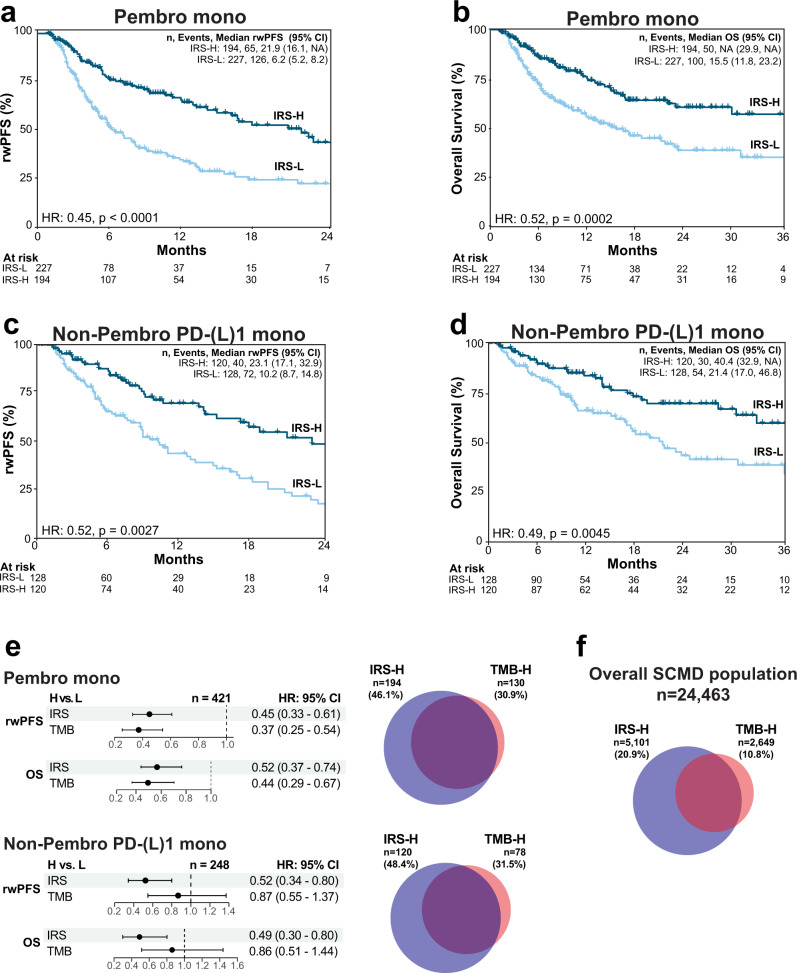
PD-[L]1 monotherapy real-world progression-free survival (rwPFS) and overall survival (OS) by immunotherapy response score (IRS) status. **a** rwPFS for monotherapy pembrolizumab (pembro; PD-1 therapy) treated patients in the discovery cohort. Pembrolizumab monotherapy rwPFS in the development cohort stratified by IRS groups is shown by Kaplan–Meier analysis with the adjusted hazard ratio (HR) and *p*-value for IRS-High [H] vs. IRS-Low [L] groups. The number (n) of patients, events, and median rwPFS (with 95% confidence intervals [CI]) for each group are shown. **b** As in a, except assessing OS. **c**, **d** As in **a**, **b**, except assessing rwPFS (**c**) and OS (**d**) in the independent validation cohort of patients treated with non-pembrolizumab PD-(L)1 monotherapy. **e** Forest plots of adjusted HRs with 95% CI for IRS and tumor mutation burden (TMB; TMB-High [H] ≥ 10 mutations/megabase) in otherwise equivalent models separately adjusted for IRS and TMB (H vs. L for each) in both cohorts for rwPFS and OS. The Venn diagrams show the number (*n*) and overlap of the IRS-H (blue) and TMB-H (red) populations in both cohorts. **f** Overlap of IRS-H and TMB-H populations in the 24,463 patients with informative IRS and TMB status (regardless of treatment status) in the Strata Clinical Molecular Database (SCMD).

As PD-(L)1 combination therapy regimens vary across tumor types and there is little evidence of even additive benefit from currently approved PD-(L)1 combination regimens^33^, while TMB has shown to be broadly predictive of monotherapy PD-(L)1 benefit^25–32^, we also restricted results in the discovery cohort to just those patients treated with pembrolizumab monotherapy (*n* = 421; 46.1% IRS-H). As shown in Fig. 2a, b, IRS-H patients had significantly longer pembrolizumab rwPFS (IRS-H vs. IRS-L median rwPFS 21.9 [95% CI: 16.1–NA] vs. 6.2 [95% CI: 5.2–8.2] months, adjusted [as for the entire cohort except for line type] hazard ratio 0.45 [95% CI: 0.33–0.61], *p* < 0.0001) and OS (IRS-H vs. IRS-L median OS Not Reached [95% CI: 29.9–NA] vs. 15.5 [95% CI: 11.8–23.2] months, adjusted hazard ratio 0.52 [95% CI: 0.37–0.74], *p* = 0.0002).

### Validation of the integrative IRS model to predict PD-1/PD-L1 blockade benefit

We next sought to validate the ability of IRS to predict PD-(L)1 monotherapy treatment outcome by both rwPFS and OS in an independent cohort. Based on a power analysis (see Methods) we identified a sufficient cohort of all 248 of the 9899 (2.5%) eligible patients in the SCMD (valid TMB and gene expression with documented anti-neoplastic agent treatment) who met the same inclusion/exclusion criteria as the discovery cohort, except they were treated with systemic non-pembrolizumab anti-PD-(L)1 monotherapy (and were not in the discovery cohort). As shown in Fig. 1a, the PD-(L)1 monotherapy validation cohort (*n* = 248; PD-1 *n* = 194 [78%] and *n* = 54 [22%] PD-L1) was comprised of patients with 24 tumor types (25% melanoma [most frequent tumor type]); tumor types and demographics are provided in Supplementary Data 2 and 3. All patients in the validation cohort were assigned to IRS-H or IRS-L groups using the locked IRS model (48.4% IRS-H), and group outcomes were compared after adjusting as for the discovery monotherapy analysis (except adding therapy type [PD-1 vs. PD-L1] as a covariate). As shown in Fig. 2c, d, by Kaplan Meier analysis, IRS-H patients had significantly longer PD-(L)1 monotherapy rwPFS (IRS-H vs. IRS-L median rwPFS 23.1 [95% CI 17.1-32.9] vs. 10.2 [95% CI: 8.7–14.8] months, adjusted hazard ratio = 0.52 [95% CI: 0.34–0.80], *p* = 0.003) and OS (IRS-H vs. IRS-L median OS 40.4 [95% CI: 32.9–NA] vs. 21.4 [95% CI: 17.0–46.8] months, adjusted hazard ratio = 0.49 [95% CI: 0.30–0.80], *p* = 0.005) compared to IRS-L patients. As described in the Supplementary Results and shown in Fig. S11, results were similar when stratifying patients by PD-1 vs. PD-L1 therapy. Taken together, these results demonstrate the development and validation of an integrative, DNA and RNA based predictor of PD-(L)1 blockade benefit, with IRS-H patients showing significantly longer rwPFS and OS in an independent validation cohort.

### Comparison of IRS to TMB for predicting PD-1/PD-L1 blockade benefit

As described above, TMB has been shown to predict both monotherapy PD-1 (pembrolizumab and nivolumab) and PD-L1 (atezolizumab) benefit through both retrospective and prospective studies, although ORRs at the same TMB cutoff vary across agents and TMB cutoffs. Hence, although quantitative TMB is a component of the IRS model, both TMB and IRS are reported as binary predictors (given the near requirement of categorical biomarkers for clinical implementation), therefore, to have clinical utility, the IRS model should identify a population of patients at least as large as the TMB-H population with similar PD-(L)1 benefit. As shown in Fig. 2e, in the 421-patient monotherapy treated subset of the discovery pembrolizumab cohort, 194 (46.1%) and 130 (30.9%) patients were identified as IRS-H and TMB-H, respectively, while in the 248-patient validation cohort, 120 (48.4%) and 78 (31.5%) patients were identified as IRS-H and TMB-H, respectively. In the pembrolizumab cohort, by Cox proportional hazards analysis, both categorical TMB (TMB-H vs. TMB-L) and IRS (IRS-H vs. IRS-L) were significant predictors of pembrolizumab monotherapy rwPFS (TMB-H vs. TMB-L adjusted hazard ratio 0.37 [95% CI: 0.25–0.54], *p* < 0.0001; IRS-H vs. IRS-L adjusted hazard ratio 0.45 [95% CI: 0.33–0.61], *p* < 0.0001) and OS (TMB-H vs. TMB-L adjusted hazard ratio 0.44 [95% CI: 0.29–0.67]; IRS-H vs. IRS-L adjusted hazard ratio 0.52 [95% CI: 0.37–0.74]; Fig. 2e) in models separately adjusted for IRS and TMB. However, in the validation cohort, IRS, but not TMB, was an independent predictor of PD-(L)1 rwPFS (TMB-H vs. TMB-L adjusted hazard ratio 0.87 [95% CI: 0.55–1.37], *p* = 0.54; IRS-H vs. IRS-L adjusted hazard ratio 0.52 [95% CI: 0.34–0.80], *p* = 0.003) and OS (TMB-H vs. TMB-L adjusted hazard ratio 0.86 [95% CI: 0.51–1.44], *p* = 0.56; IRS-H vs. IRS-L adjusted hazard ratio 0.49 [95% CI: 0.30–0.80], *p* = 0.005, Fig. 2e) in models separately adjusted for IRS and TMB (Kaplan-Meier plots of rwPFS and OS stratified by TMB status are shown in Figure S6c & d). As shown in Fig. 2f, across 24,463 Strata Trial samples in the SCMD with informative TMB and gene expression (regardless of treatment data availability), the overall IRS-H population was nearly twice as large as the TMB-H population (20.9% vs. 10.8%). Kaplan–Meier analysis of the discovery and validation cohorts stratified by IRS and TMB status are shown in Fig. S12 and described in the Supplementary Results.

Taken together, these results demonstrate that in both the discovery and independent validation cohorts, IRS identifies a larger proportion of patients than TMB alone with similar benefit from PD-(L1) therapy, establishing clinical utility of the IRS biomarker and demonstrating the value of integrating quantitative gene expression with TMB for predicting PD-1/PD-L1 monotherapy treatment benefit. As described in the Supplementary Results and Fig. S13, *CDKN2A* deep deletion (homozygous loss) status, which has improved upon TMB alone for predicting monotherapy PD-(L)1 benefit in two recent studies^59,60^, was not additive to IRS, further supporting the limitations of genomic markers alone for predicting PD-(L)1 therapy response. Additional analyses supporting the robustness of the IRS model to temporal sample collection (prior to CPI treatment) and variable tumor content are described in the Supplementary Results and Figs. S14 and S15.

### Confirmation of the predictive nature of IRS

**Fig. 3:**
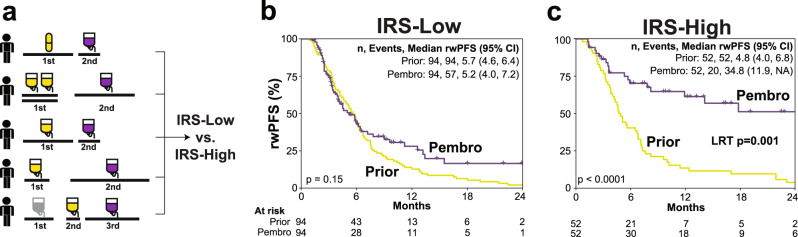
Confirmation of the predictive nature of the immunotherapy response score (IRS) biomarker. To establish the predictive nature of the IRS model, we assessed an internal comparator in the pembrolizumab monotherapy cohort, consisting of the 146 patients who had received a prior line of systemic therapy prior to pembrolizumab monotherapy (*n* = 146 individual patients). **a** For each patient, real-world progression-free survival (rwPFS) was determined for the line of systemic therapy immediately prior to pembrolizumab (pembro; yellow) and the pembrolizumab monotherapy line (purple), with rwPFS for each group then stratified by IRS status. **b** Kaplan–Meier analysis of pembrolizumab monotherapy rwPFS (purple) vs. prior systemic therapy rwPFS (yellow) in the IRS-Low [L] subset of patients (log-rank p-value shown). The number (*n*) of patients, events, and median rwPFS (with 95% confidence intervals [CI]) for each group are shown. **c** Kaplan–Meier analysis of pembrolizumab monotherapy rwPFS (purple) vs. prior systemic therapy rwPFS (yellow) in the IRS-H subset of patients (log-rank *p*-value shown). The likelihood ratio test (LRT) *p*-value for interaction between pembrolizumab vs. immediately prior treatment line and IRS status (IRS-L vs. IRS-High [H]) is also shown.

To establish the IRS model as predictive and not prognostic, we first assessed an internal comparator cohort for the pembrolizumab monotherapy cohort, consisting of the 146 of 648 (22.5%) of patients who had received a previous line of systemic therapy prior to pembrolizumab monotherapy (demographics and therapy types are shown in Supplementary Data 4). For each patient, rwPFS was determined for the line of systemic therapy immediately preceding pembrolizumab and the pembrolizumab monotherapy line, with rwPFS stratified by IRS status assessed by Kaplan–Meier analysis (Fig. 3a). While pembrolizumab monotherapy compared to the immediately preceding therapy line rwPFS did not significantly differ in IRS-L patients (IRS-L pembrolizumab vs. immediately preceding therapy median rwPFS 5.2 [95% CI: 4.0–7.2] vs. 5.7 [95% CI: 4.6–6.4] months, log-rank *p* = 0.15; Fig. 3b), pembrolizumab rwPFS was significantly longer than the immediately preceding therapy line in IRS-H patients (IRS-H pembrolizumab vs. immediately preceding therapy median rwPFS 34.8 [95% CI: 11.9–NA] vs. 4.8 [95% CI: 4.0–6.8] months, log-rank *p* < 0.0001; Fig. 3c). The test for interaction (models shown in Table S5) between pembrolizumab vs. immediately preceding treatment line and IRS status (IRS-H vs. IRS-L) was significant (likelihood ratio test for interaction *p* = 0.001). Notably, when this analysis was restricted to the 46 patients with non-MSI-H (StrataNGS clinical testing) tumors in non-PD(L)1 monotherapy approved tumor types, only IRS-H patients still had significantly longer pembrolizumab monotherapy rwPFS than the immediately preceding line of therapy (IRS-H pembrolizumab vs. immediately preceding therapy median rwPFS 11.9 [95% CI: 7.8–NA] vs. 3.2 [95% CI: 2.3–9.6] months, log rank, *p* = 0.005; Fig. S16a, b). Additional analyses supporting the predictive nature of the IRS biomarker, including a lack of significant association with IRS status and non-PD-(L)1 or CTLA4 systemic therapy rwPFS in >3000 SCMD patients, are described in the Supplementary Results and shown in Fig. S16c, d. Taken together, these results confirm the predictive nature of the IRS biomarker across tumor types.

### Exploratory analysis of IRS in patients with first-line non-small cell lung cancer (NSCLC)

**Fig. 4:**
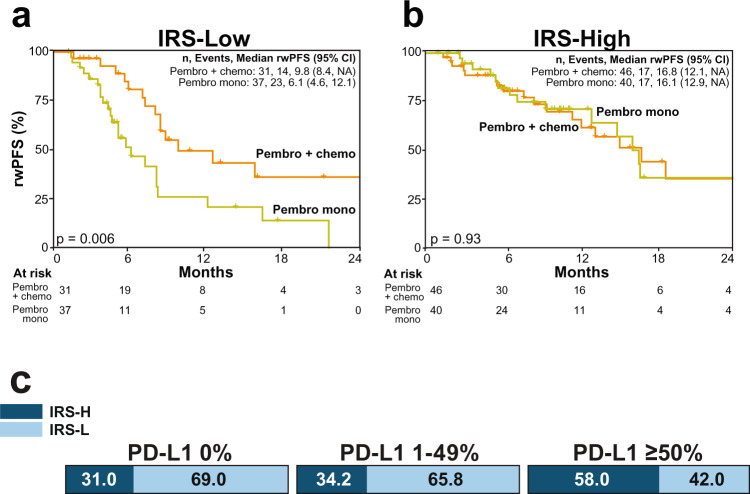
Immunotherapy response score (IRS) for predicting pembrolizumab monotherapy vs. combination chemotherapy benefit in first line non-small cell lung carcinoma (NSCLC). Propensity score matching (see Methods) was used to identify matched cohorts of patients with NSCLC treated with first line systemic pembrolizumab (pembro) monotherapy (*n* = 77 patients) or pembrolizumab + chemotherapy (chemo) combination therapy (*n* = 77 patients) that did not significantly differ in age, gender, tumor mutation burden (TMB) status, *PD-L1* expression by quantitative transcriptomic profiling (qTP; the expression biomarker component of IRS), or IRS status; PD-L1 immunohistochemistry (IHC) was only available for 24/154 samples in the matched cohort (see Fig. S8 for validation of PD-L1 by qTP vs. PD-L1 IHC). **a** Kaplan–Meier analysis of pembrolizumab monotherapy real-world progression-free survival (rwPFS; orange) vs. pembrolizumab + chemotherapy combination therapy (yellow) in the IRS-Low [L] subset of patients (log-rank *p*-value shown). The number (*n*) of patients, events, and median rwPFS (with 95% confidence intervals [CI]) for each group are shown. **b** as in **a**, except the IRS-High [H] subset of patients. **c** Distribution of IRS status in a separate cohort of *n* = 276 NSCLC tumor samples with PD-L1 IHC (Fig. S8) stratified by clinically relevant tumor proportion score (TPS) bins.

As described above, despite little, if any, evidence for additive or synergistic benefit of PD-(L)1 and other agents in approved combination regimens, PD(L)-1 combination regimens are rapidly being developed and moved to earlier lines of therapy, highlighting the need for improved biomarkers that can predict PD-(L)1 monotherapy benefit. For example, in first line advanced NSCLC, both monotherapy pembrolizumab and pembrolizumab + chemotherapy are approved for patients with PD-L1 IHC (TPS) ≥ 1% and ≥50%, however prospective data is not available to guide monotherapy vs. combination therapy decision making. Hence, in the pembrolizumab cohort, we identified 242 patients with NSCLC who were treated with first line systemic pembrolizumab monotherapy (*n* = 109) or pembrolizumab + chemotherapy (*n* = 133; Fig. S1). Although this cohort is limited by a lack of PD-L1 TPS data, IRS includes qTP expression of *PD-L1*, and we have validated the accuracy of this individual transcript vs. TPS in NSCLC FFPE tumor samples (Fig. S8). Consistent with both TPS and performance status largely driving the monotherapy vs. combination therapy treatment decision, we confirmed that monotherapy treated patients were significantly older and had higher PD-L1 qTP expression compared to combination therapy treated patients (Table S6). Hence, we performed propensity score matching (see Methods) between the monotherapy and combination therapy groups using patient age, PD-L1 qTP expression, TMB, gender and IRS, which after excluding 88 unmatchable patients resulted in a final cohort of 154 patients (77 patients in each group) without significant differences in any of these variables (Table S6). As shown by Kaplan Meier analysis of the matched cohorts, in IRS-L patients, rwPFS was significantly shorter in those treated with monotherapy vs. combination therapy (median rwPFS 6.1 [95% CI: 4.6–12.1] vs. 9.8 [95% CI: 8.4-NA] months, log rank *p* = 0.006; Fig. 4a). In contrast, in IRS-H patients, rwPFS was not significantly different in those treated with monotherapy vs. combination therapy (median rwPFS 16.1 [95% CI: 12.9–NA] vs. 16.8 [95% CI: 12.1–NA] months, log rank *p* = 0.93; Fig. 4b). Taken together, these results support pembrolizumab monotherapy as a potentially reasonable treatment option for the 34% of patients with TPS scores 1-49% who are IRS-H (Fig. 4c and S8), consistent with a recent report assessing TMB across PD-L1 IHC strata in patients with first line NSCLC treated with PD-(L)1 monotherapy^27^, and more broadly suggests potential utility in identifying patients who may benefit from monotherapy PD-(L)1 vs. combination therapy in current indications.

### Pan solid tumor distribution of IRS groups

**Fig. 5:**
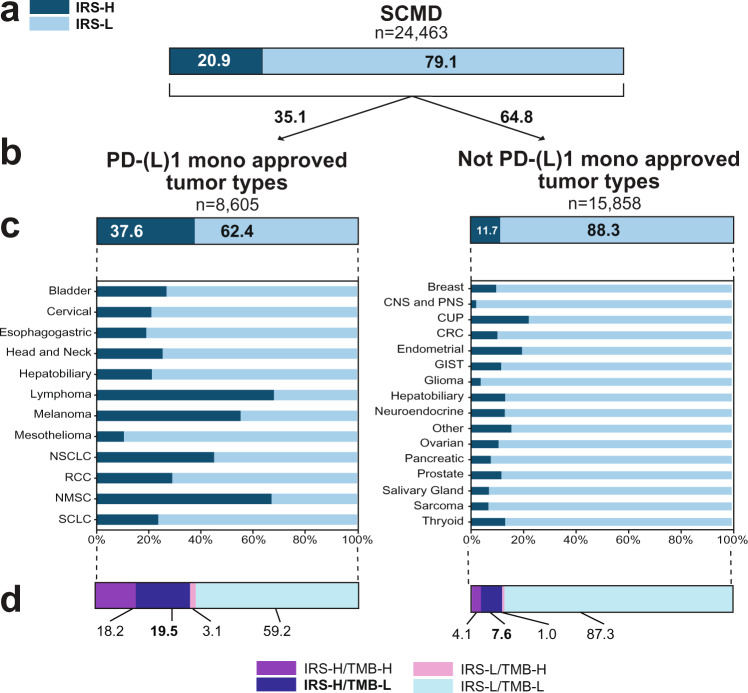
Pan-solid tumor distribution of immunotherapy response score (IRS) groups. **a** IRS groups were determined for all 24,463 patients in the Strata Clinical Molecular Database (SCMD) with informative tumor mutation burden (TMB) and gene expression data needed to generate IRS. IRS group (Low [L; light blue] vs. High [H; dark blue]) distribution is shown by box plot (numbers indicated percentages). **b** Stratification of the 24,463 patients by approved and non-approved PD-(L)1 monotherapy tumor types. **c** Breakdown of **b** by individual tumor types. **d** Breakdown of **b** by IRS and TMB (High [H] vs. Low [L]; TMB-H as ≥10 mutations/megabase). Results may not add up to 100% or be equivalent in sub-analyses due to rounding. Tumor type abbreviations: NSCLC (non-small cell lung cancer), RCC (renal cell carcinoma), NMSC (non-melanoma skin cancer), SCLC (small cell lung cancer), CNS and PNS (central nervous system and peripheral nervous system), CUP (cancer of unknown primary), CRC (colorectal cancer), GIST (gastrointestinal stromal tumor).

In both the discovery and separate validation cohorts, we demonstrated that IRS identifies a larger population of patients than TMB but with similar PD-(L)1 monotherapy benefit, however this analysis is limited by the requirement that patients received PD-1/PD-L1 treatment. Hence, we sought to leverage IRS distributions across tumor types (and pan-cancer biomarkers) in the entire SCMD to understand the potential impact of IRS both within and outside of currently approved PD-(L)1 monotherapy indications. Thus, we determined IRS for the 24,463 patients in the SCMD (NCT03061305) with informative TMB and gene expression data, with 20.9% and 79.1% of all patients classified as IRS-H and -L, respectively (Fig. 5a). PD-(L)1 monotherapy approved tumor types^61^ (without consideration of PD-L1 IHC status) had a substantially higher proportion of IRS-H patients (37.6%) than non-PD-(L)1 monotherapy approved tumor types (11.7%) (Fig. 5b). Tumor types with the highest proportion of IRS-H group patients include several known to be highly responsive to PD-(L)1 therapy, including lymphoma, non-melanoma skin cancer, melanoma, NSCLC, and renal cell carcinoma (which nearly invariably has low TMB) (Fig. 5c).

We lastly examined the pan-solid tumor distribution of IRS groups by TMB status, given the pan-tumor approval of pembrolizumab in TMB-H tumors and prospective trials showing efficacy of other PD-(L)1 monotherapies patients with TMB-H. In both PD-(L)1 monotherapy approved and non-approved tumor types, the vast majority of TMB-H patients were also IRS-H (only 1.8% of overall patients were IRS-L/TMB-H [3.1% and 1.0% in approved and non-approved tumor types, respectively), however the overall IRS-H population was nearly twice as large as the TMB-H population (20.9% IRS-H vs. 10.8% TMB-H overall [Fig. 2f]; 37.7% IRS-H vs. 22.6% TMB-H in approved tumor types and 11.7% IRS-H vs. 5.1% TMB-H in non-approved tumor types, respectively; Fig. 5d) with similar PD-(L)1 monotherapy benefit as established herein. Critically, this analysis demonstrates that 7.6% of patients in non-approved tumor types are IRS-H/TMB-L, representing a sizable population predicted to have benefit from PD-(L)1 monotherapy.

## Discussion

Leveraging a robust clinical molecular database from the Strata Trial (NCT03061305), herein we developed an integrative Immunotherapy Response Score (IRS) algorithm combining TMB and quantitative gene expression from simultaneously performed, clinically validated, multiplex PCR based DNA and RNA NGS (StrataNGS CGP and a separate RNA panel for quantitative transcriptomic profiling)^54–56^ to predict pembrolizumab (anti-PD-1) rwPFS (by time to next therapy) in 648 patients from 26 solid tumor types. We then validated the locked IRS model—which incorporates TMB and quantitative gene expression of *PD-1*, *PD-L1*, *ADAM12*, and *TOP2A* in a Cox proportional hazards model—and IRS-H vs. -L threshold (IRS-H as more likely to benefit) in an independent cohort of 248 patients from 24 solid tumor types treated with other PD-(L)1 monotherapies. In this validation cohort, IRS-H status was associated with significantly longer PD-(L)1 rwPFS (IRS-H vs. IRS-L median 23.1 vs. 10.2 months; adjusted hazard ratio 0.52, *p* = 0.003) and OS (median OS 40.4 vs. 21.4 months, adjusted hazard ratio 0.49, *p* = 0.005) when adjusted for age, gender, line of therapy, PD-1 vs. PD-L1 therapy, and tumor type. Notably, TMB alone was not a significant predictor of PD-(L)1 rwPFS, nor OS, in this cohort. When applied to all 24,463 patients in the SCMD where IRS could be generated, the IRS-H population was nearly twice the size of the TMB-H population (20.9 vs. 10.8%).While IRS-H was more frequent in tumor types known to derive benefit from PD-(L)1 therapy, IRS-H occurred in subsets of nearly every tumor type. Most importantly, among TMB-L patients in tumor types without approved PD-(L)1 monotherapy, 7.6% were IRS-H (a potentially conservative estimate as many approved indications have PD-L1 IHC requirements), representing a substantial population of patients with advanced solid tumor who could immediately benefit from PD-(L)1 monotherapy treatment.

We confirmed the predictive nature of the IRS biomarker through multiple approaches. Most importantly, in the subset of pembrolizumab monotherapy treated patients who had at least one prior line of systemic therapy, we confirmed the predictive nature of the IRS model, as IRS-H patients had significantly longer rwPFS on pembrolizumab vs. their immediately preceding systemic therapy, with a significant test for interaction between IRS and pembrolizumab vs. prior therapy. Likewise, IRS status was not significantly associated with first line rwPFS in >3000 patients treated with non-immunotherapy. Although the association of IRS with PD-(L)1 rwPFS and OS were similar in the discovery (pembrolizumab) and validation (non-pembrolizumab PD-(L)1 monotherapy) cohorts and was largely insensitive to tumor type, TMB status, and pre- vs. post-non CPI therapy sample collection—suggesting that the model captures universal biological features of PD-(L)1 monotherapy benefit—the model was less predictive in patients treated with pembrolizumab combination therapy (combination and monotherapy pembrolizumab rwPFS adjusted hazard ratio 0.60 [95% CI: 0.41-0.89], *p* = 0.01 and 0.45 [95% CI: 0.33-0.61], *p* < 0.0001, respectively) and did not significantly predict combination PD-1 + CTLA4 (nivolumab + ipilumumab) benefit. These results suggest that while different approaches are likely needed to best predict combination therapy (or monotherapy of the non-IO component) responses, particularly in light of a recent meta-analysis demonstrating that there is little evidence for synergy between CPIs and other agents in approved combination regimens^33^, the IRS model can likely identify patients expected to benefit from PD-(L)1 monotherapy in settings where only combination (or both monotherapy and combination) PD-(L)1 therapy is indicated. In support of this, in an exploratory analysis on a propensity score matched cohort of patients with NSCLC treated with first line systemic pembrolizumab monotherapy vs. combination chemotherapy, while rwPFS was significantly worse in IRS-L patients treated with monotherapy vs. combination chemotherapy, no significant difference in rwPFS was present in IRS-H patients treated with monotherapy vs. combination chemotherapy. In a separate cohort of patients with NSCLC, we demonstrate that approximately one third of those with PD-L1 IHC TPS 1–49% (where the monotherapy vs. combination chemotherapy decision is most relevant) are IRS-H, suggesting potential utility in identifying those patients most likely to benefit from pembrolizumab monotherapy alone.

Current FDA-approved PD-(L)1 biomarkers include PD-L1 IHC, TMB, and MSI-H (the latter indication was initially approved without a companion diagnostic biomarker), however these biomarkers have several practical challenges for clinical use including variations in assay parameters, platforms, and predictive thresholds^4,62–65^. For example, although there are multiple tissue TMB assays commercially available (LDTs, FDA cleared devices, and a single FDA approved companion diagnostic device), TMB testing typically has a large tissue requirement, which is frequently not feasible in patients with advanced cancers, and such approaches do not allow for parallel clinical assessment of gene expression biomarkers. Likewise, liquid biopsy based TMB is not directly translatable to tissue TMB, even when both tissue and liquid biopsies are performed using FDA approved CGP devices, as in a recent study of both single agent nivolumab and nivolumab + ipilimumab combination therapy, where blood TMB’s predictive ability was conditional on tissue TMB status, but not vice versa^32^. Hence, it is notable that our study herein used assays performed as part of routine clinical testing on co-isolated DNA and RNA, and are now integrated in a combined analytically and validated clinical CGP + qTP test with key sample input requirements defined from over 30,000 consecutively received FFPE tumor samples for CGP testing: ≥20% tumor content and 2 mm^2^ tumor surface area (from 10 × 5 µm FFPE sections)^55,56^ Of note, only 37.5% and 43.5% of the discovery and validation cohort, respectively, and 35.5% of the 24,463 total patients in the SCMD used to assess IRS distribution, met the minimum tumor surface area requirements (≥25 mm^2^) of FoundationOne CDx^66^, the FDA approved companion diagnostic device to identify TMB-H tumors for pembrolizumab treatment. Although outside the scope of the current manuscript, integration of clinically validated qTP also has clinical utility outside of immunotherapy treatment decision making for patients with advanced solid tumors (Fig. S17), however, detailed discussion is outside the scope of the current manuscript.

Our analysis has several potential limitations. First, our real-world Strata Trial treatment dataset was biased toward tumor types for which PD-(L)1 therapy is indicated, and thus, as expected, was enriched for patients benefiting from PD-(L)1 therapy. Indeed, the proportion of IRS-H patients was greater in the discovery and validation cohorts (combined 46.7%) than the broader Strata Trial profiling dataset (20.9%). However, patients with more than 20 tumor types were included in both the discovery and validation cohorts, and these cohorts consisted of both pembrolizumab and other non-pembrolizumab PD-(L)1 monotherapy-treated patients respectively. Additionally, we confirmed the predictive nature IRS in the greater than first line, off label (non-MSI-H, non-approved tumor types) population, similar to the pivotal study of pembrolizumab in the TMB-H population^26^. Second, the rwPFS endpoint includes some patients who stopped treatment due to treatment toxicity (not assessable herein) or switching therapy to a more appropriate regimen based on molecular results (as described in the Supplementary Results) and not disease progression, although this likely represents a minority of events, and both rwPFS and OS results were highly similar in both the discovery and validation cohorts. Additionally, although we developed and validated the IRS model across patients treated with multiple PD-(L)1 monotherapies and tumor types, not all solid tumor types were represented in these analyses and prospective studies could determine if more optimized thresholds (or further stratification beyond two IRS groups) may improve performance in specific tumor types or better predict PD-1 vs. PD-L1 therapy benefit. Of note, IRS had essentially similar predictive ability in both the training and validation cohorts (Table S7) when the tumor type term in our adjusted models (most common tumor type vs. others) was replaced with a term using MSKCC defined TMB sensitive vs. insensitive tumor types (MSI-H, *POLE*^mutatnt^, NSCLC, head and neck cancer or melanoma as sensitive; all others as insensitive^57^), supporting the more pan-solid tumor nature of IRS vs. TMB alone. Likewise, although we showed that the inclusion of *CDKN2A* copy loss, which has been identified in two studies as improving upon TMB status for predicting PD-(L)1 response^59,60^, was not a significant predictor of PD-(L)1 rwPFS or OS in either the discovery or validation cohorts, future studies will be required to determine whether inclusion of other single gene-based DNA biomarkers identified as potentially predictive in one or more tumor types (e.g. *STK11*, *PBRM1*, and *ARID1A)*^59,60,67–73^ or additional immune related genes assessed on the current expanded qTP panel can improve the performance of the IRS model; given the clearly established clinical utility for MSI-H status, this biomarker was not included in IRS model development. Limited PD-L1 IHC data was available for subjects in the SCMD with PD-(L)1 treatment outcomes, and hence we are not able to directly compare performance of IRS and PD-L1 IHC (or other immunotherapy response biomarkers beyond TMB), which is particularly relevant for our exploratory analysis of pembrolizumab monotherapy vs. combination therapy in first line NSCLC, however we used propensity score matching by PD-L1 qTP expression to mitigate this limitation. Notably, we chose to use standard multivariate regression with a minimum number of variables versus other approaches that have included a larger number of immune related genes^34,41,46^ or used more advanced machine learning approaches^74^ to leverage the highly quantitative nature of CGP + qTP and minimize the risk of overfitting and additional biological insights derived from the IRS model are described in the Supplementary Discussion. Importantly, although demonstration that IRS predicts PD-(L)1 monotherapy rwPFS and OS at least as well as TMB in both the discovery and independent validation cohorts establishes clinical utility in the 7.6% of IRS-H/TMB-L patients outside of currently approved PD-(L)1 monotherapy indications, additional studies will be required to establish the clinical utility of IRS-H in patients with conflicting biomarker results (e.g. IRS-H/TMB-L) or where both monotherapy and combination therapy are indicated (e.g. IRS-H in PD-L1 IHC 1-49%). Likewise, in a post-hoc, exploratory analysis in the combined discovery and validation cohorts, we identified an ultra-low subset of the IRS-L population that shows particularly poor PD-(L)1 rwPFS and OS (Figure S18 & S19), suggesting that it may be possible to identify patients more likely to benefit from other therapies in PD-(L)1 approved tumor types when therapeutic choice is present. Together, these limitations, which are largely due to the retrospective nature of this study from an observational clinical trial (lack of randomization, selection and confounding biases, variability in tumor types and therapies, lack of actual progression event data, etc), support continued assessment of IRS through additional blinded prospective/retrospective studies and prospective studies in both pan-solid tumor and tumor-type specific indications, such as ongoing studies in NSCLC (NCT03793179) and basket studies used to demonstrate utility of TMB alone^25,26,31,32^.

In summary, using treatment data and molecular profiling from nearly 900 patients in the Strata Trial, a large observational trial of patients with advanced cancer, we report the development and validation of IRS, a biologically rational, integrative predictor of pan-solid tumor PD-(L)1 monotherapy benefit (by both rwPFS and OS) across solid tumors that identifies a population that is nearly twice as large as TMB-H alone with similar PD-(L)1 monotherapy benefit. Importantly, IRS was developed and validated using a single, clinically validated NGS platform capable of simultaneously performing CGP (required for TMB but also for assessing non-immunotherapy treatment biomarkers) and simultaneous, precise quantification of tumor- and tumor microenvironment (TME)-relevant gene expression from minute FFPE tumor specimens. In addition to potential utility of IRS for refining treatment decisions in patients with approved PD-(L)1 indications, we show that across the >20,000 patient Strata Trial population with evaluable IRS status, 7.6% of patients with tumor types not approved for PD-(L)1 monotherapy were IRS-H/TMB-L—a population shown herein to have similar or better PD-(L)1 benefit as TMB-H—markedly expanding the benefit of immunotherapy across solid tumors by addressing one of the most important challenges in precision oncology.

## Supplementary information

Description of Additional Supplementary Files

Supplementary Data 1

Supplementary Information

Supplementary Data 2

Supplementary Data 3

Supplementary Data 4

Supplementary Data 5

Reporting Summary

## Data Availability

Due to applicable data sharing agreements and/or patient informed consent forms with Strata Trial health care systems and participants, the authors are restricted from making raw patient-level genomic sequencing data publicly available or deposited. Interested parties may contact the authors at BD@strataoncology.com to request access for research purposes, and such requests will be handled on a case-by-case basis. All clinical and treatment data for the discovery and validation cohorts described herein (including the raw TMB and expression biomarker data used to derive the IRS algorithm output) are available in Supplementary Data 5; this file also provides source data for all figures on separate tabs. All other data are available from the corresponding author on reasonable request.

## Code availability

The IRS model algorithm is available through GitHub [10.5281/zenodo.7510725].
