# Supplementary material for: Development and validation of an integrative pan-solid tumor predictor of PD-1/PD-L1 blockade benefit
Source: Commun Med (Lond). 2023 Feb 7;3:14. doi: 10.1038/s43856-023-00243-7 (PMC9905474; doi:10.1038/s43856-023-00243-7)
Supplement: Supplementary file 1 — Description of Additional Supplementary Files [file 43856_2023_243_MOESM1_ESM.pdf]

Description of Additional Supplementary Files

File Name: Supplementary Data 1

Description: Central and Local Institutional Review Board (IRB) Review of the Strata Trial (NCT03061305)

Central Review of NCT03061305 was performed by Advarra IRB (IRB Pro00019183) with waiver of informed consent. For all participating sites, whether Review was ceded to the Central IRB or performed by a Local IRB is indicated. Whether informed consent was required by the Local IRB (where not ceded) or by individual sites is indicated.

File Name: Supplementary Data 2

Description: Tumor types of study cohorts

Breakdown by StrataNGS CGP assigned primary tumor type (total number and percentage of each cohort) for the cohorts used for discovery, validation, and additional analysis. The total number of tumor types for each cohort includes each primary tumor type for which there was at least one sample. Cohort total and total number of tumor types indicated by bolded text. IRS= Immunotherapy Response Score, n= sample size, %= percentage, PD-(L)1= PD-L1 or PD-1 therapy, Nivo. + ipi. .= nivolumb + ipilimumab, IO=immunotherapy (PD-[L]1 or CTLA4 therapy), CNS= central nervous system, PNS= peripheral nervous system.

File Name: Supplementary Data 3

Description: Demographics of study cohorts

Demographics considered for each cohort included gender, race, ethnicity, and line of therapy. For the discovery cohort, both pembrolizumab monotherapy and combination therapy containing lines were included. For the PD-(L)1 validation cohort, only PD-1 or PD-L1 monotherapy (excluding pembrolizumab) were included. . IRS= Immunotherapy Response Score, n= sample size, %= percentage, PD-(L)1= PD-L1 or PD-1 therapy, Nivo. + ipi. .= nivolumb + ipilimumab, IO=immunotherapy (PD-[L]1 or CTLA4 therapy). std= standard deviation, min= minimum, max= maximum. * Percentages based on PD-1 or PD-L1 participant totals, respectively.

File Name: Supplementary Data 4

Description: Characteristics of the internal comparator cohort (n=146) consisting of patients treated with pembrolizumab monotherapy who had prior systemic therapy

Variable category indicated by bold text. n= sample size, %= percentage.

File Name: Supplementary Data 5

Description: Clinical, Treatment, and Molecular Data for Discovery (n=648) and Validation (n=248) Cohort Subjects

Clinical, treatment, and Immunotherapy Response Score (IRS) relevant molecular data for all subjects included in the Discovery (n=648) and Validation (n=248) Cohorts are provided in the first tab. Subject age (years; binned by decade), gender, primary tumor type, and inclusion in the discovery or validation cohort is shown. For PD-(L)1 therapy lines used for IRS discovery or validation (or the line of systemic therapy immediately preceding the pembrolizumab containing line in the Discovery cohort), the line of systemic therapy (1st, 2nd, etc), the type of line (ab=antibody, angio=angiogenesis inhibitor, chemo=chemotherapy, hormonal = hormonal therapy, io=immunotherapy, oncogene=oncogene targeted TKI, multi-kinase=multikinase TKI), the included PD-(L)1 therapy in the line (NA if the line immediately preceding pembrolizumab), monotherapy status of the line, real world progression free survival (rwPFS; by time to next therapy [TTNT] in months), rwPFS event status (TRUE indicates progression event occurred; FALSE indicates censored), overall survival (OS; in months), OS event status (TRUE indicated death occurred; FALSE indicates censored), and total follow-up (in months) are provided. For subjects with a previous line of systemic therapy prior to pembrolizumab monotherapy, the therapy information (additional type of lines include: angio-ab= angiogenesis inhibiting antibodies, vaccine = vaccine/cell therapy) for the line immediately preceding pembrolizumab is also provided on a separate row with the same Subject Identifier. Tumor Mutation Burden (TMB) status (≥10 mutations/megabase [Muts/Mb] = TMB-High), microsatellite instability (MSI) status (MSS = microsatellite stable; MSI-High = microsatellite instable), MSKCC definition of TMB sensitive tumor types (MSI-H, *POLE*^{mutant}, Non-small cell lung cancer, head and neck cancer, or melanoma = TMB-sensitive; all other samples as TMB-insensitive [PMID: 33761214]), *CDKN2A* deep deletion status (present = CDKN2A deep deletion; absent = CDKN2A not deep deletion; N/A deep deletion status not evaluable due to QC or tumor content <40%), tumor content, IRS status (-High or -Low) and IRS algorithm score (-High ≥ 0.873569) are also provided. The five biomarker components of IRS are provided ($\log_2$ TMB [in Muts/Mb] and median scaled, $\log_2$ housekeeping gene normalized expression of ADAM12, CD274, TOP2A and PDCD1). For TMB, the TMB [Muts/Mb], the lower and upper confidence intervals [CI; Muts/Mb] for the TMB point estimate, and the effective panel size (in Mb) used in the TMB calculation are shown. For the four target genes included in IRS, the raw end-to-end mapping amplicon read count, the housekeeping gene normalized reads per million (nRPM), and the $\log_2$ housekeeping gene nRPM values are provided. For the three housekeeping genes (*HMBS*, *CIAO1*, *EIF2B1*) used in IRS, the raw end-to-end mapping amplicon read counts are provided. The total number of end-to-end mapping reads on the RNA panel used to generate IRS are also shown. The remaining Individual tabs correspond to the indicated primary data used to generate each figure panel. For Fig1a and Fig5, Figure_Tumor_Type indicates the tumor type per the figure. For Fig2f and Fig5, the SCMD_ID provides an ID (from the overall 24,463 IRS evaluable population) that is distinct from the Subject_Identifier that is used in the remainder of the study. For Fig4c, PD-L1 IHC categories are indicated. For Fig5 both the Primary Tumor Type and Secondary Tumor Type are provided.
