# Supplementary material for: Development and validation of an integrative pan-solid tumor predictor of PD-1/PD-L1 blockade benefit
Source: Commun Med (Lond). 2023 Feb 7;3:14. doi: 10.1038/s43856-023-00243-7 (PMC9905474; doi:10.1038/s43856-023-00243-7)
Supplement: Supplementary file 3 — Supplementary Information [file 43856_2023_243_MOESM3_ESM.pdf]

Development and Validation of an Integrative Pan-Solid Tumor Predictor of PD-1/PD-L1 Blockade Benefit

Scott A. Tomlins[1*], Nickolay Khazanov[1], Benjamin J. Bulen[1], Daniel H. Hovelson[1], Melissa J. Shreve[1], Laura E. Lamb[1], Marc R. Matrana[2], Mark E. Burkard[3], Eddy Shih-Hsin Yang[4], William Jeffery Edenfield[5], E. Claire Dees[6], Adedayo A. Onitilo[7], Michael Thompson[8], Gary L. Buchschacher[9], Alan M. Miller[10], Alexander Menter[11], Benjamin Parsons[12], Timothy Wassenaar[13], Leon C. Hwang[14], J. Marie Suga[15], Robert Siegel[16], William Irvin, Jr.[16], Suresh Nair[17], Jennifer N. Slim[18], Jamal Misleh[19], Jamil Khatri[20], Gregory Masters[19], Sachdev Thomas[15], Malek Safa[21], Daniel M. Anderson[22], Kat Kwiatkowski[1], Khalis Mitchell[1], Tina Hu-Seliger[1], Stephanie Drewery[1], Andrew Fischer[1], Komal Plouffe[1], Eric Czuprenski[1], Jennifer Hipp[1], Travis Reeder[1], Hana Vakil[1], D. Bryan Johnson[1], Daniel R. Rhodes[1*]

SUPPLEMENTARY INFORMATION

SUPPLEMENTARY METHODS

SUPPLEMENTARY RESULTS

SUPPLEMENTARY DISCUSSION

SUPPLEMENTARY REFERENCES

SUPPLEMENTARY FIGURE LEGENDS

SUPPLEMENTARY FIGURES 1-19

SUPPLEMENTARY TABLES 1-7

SUPPLEMENTARY METHODS

STRATA CLINICAL MOLECULAR DATABASE (SCMD) VALIDITY ANALYSIS

Analyses to assess the clinical validity of the SCMD included an analysis of real-world progression-free survival (rwPFS; by time to next therapy) in patients with non-small cell lung cancer (NSCLC) treated with systemic first-line, first-generation monotherapy tyrosine kinase inhibitor (TKI) against *EGFR*, *ALK*, *ROS1* or *MET* (i.e., erlotinib, gefitinib or crizotinib) vs. those treated with later-generation monotherapy (i.e., afatinib, alectinib, brigatinib, capmatinib, ceritinib, entrectinib, lorlatinib, osimertinib or tepotinib). Next, we assessed rwPFS in patients with NSCLC treated with systemic first-line, current National Comprehensive Cancer Network (NCCN; v3.2022) preferred first-line targeted oncogene TKI (i.e., osimertinib, afatinib, alectinib, brigatinib, capmatinib, entrectinib, dabrafenib + trametinib, entrectinib, lorlatinib, selpercatinib or tepotinib), based on whether the line of therapy started before or after completion of StrataNGS testing. Lastly, we assessed rwPFS in patients with NSCLC treated with biomarker (by StrataNGS testing) matched, systemic first-line, current preferred first-line targeted oncogene TKI (with therapy line start date after completion of StrataNGS testing), stratified by whether the sample: 1) passed both StrataNGS sample input criteria (≥2mm^2 tumor surface area, ≥1ng/ul isolated DNA or RNA, sample age ≤5yrs) and relevant sequencing QC metrics (≥20% tumor content, mutation QC pass [for 1st line standard of care mutations], and fusion QC pass [for 1st line standard of care fusions]) or 2) did not meet sample input or sequencing QC metrics but reported a therapy matched biomarker.

VALIDATION OF QUANTITATIVE TRANSCRIPTOMIC PROFILING (QTP) AND IRS INTEGRATED COMPREHENSIVE GENOMIC PROFILING (CGP) + QTP TEST

INTRODUCTION

The integrative comprehensive genomic profiling and quantitative transcriptomic profiling (CGP + qTP) laboratory developed test (LDT) is performed in a Clinical Laboratory Improvement Amendments (CLIA) certified, College of American Pathologist (CAP) accredited laboratory. The test is performed on

co-isolated DNA and RNA from FFPE tissue tumor specimens and simultaneously assesses mutations (SNVs, short [<40bp] indels and SSVs), CNAs (amplifications or deep deletions [equivalent to homozygous loss if diploid]), MSI status, and TMB (in mutations/megabase [Muts/Mb]), along with qTP of individual target genes; the integrative IRS algorithm is also reported by combining TMB and target gene expression of four amplicons as described in the **Methods**. Three multiplexed PCR-based panels (two DNA and one RNA) are used, with all three libraries per sample combined prior to sequencing; all variant classes are analyzed and subjected to independent quality control (QC) metrics and bioinformatics pipelines for reporting as a single integrative test report. Analyses related to the analytical and clinical validation of the general qTP component (where relevant to IRS) as well as the integrative IRS model are described herein.

Specimen evaluation, processing, and molecularly informed tumor content determination, nucleic acid isolation, quantification, normalization, library preparation, sequencing, quality control and CGP reporting are essentially as described in the validation of the StrataNGS CGP test[1]. For tumor content determination, briefly, given the challenges of estimating tumor content in difficult samples, the final molecularly informed tumor content is set after completion of StrataNGS testing, initially incorporating variant allele frequencies (VAF) and copy state of mutations in tumor suppressors known to nearly always be homozygous[2], with subsequent refinement integrating these results with the remainder of the mutational and copy number profiles along with b-allele frequencies from high confidence SNPs[1]. For all cases in the SCMD (including those used in IRS development and validation) the final MTC was set by a single pathologist (S.A.T.) with automatic updating to all applicable variants/ variant classes.

The current CGP component of the CGP + qTP is an updated version of the validated StrataNGS CGP test, which is covered for Medicare beneficiaries with advanced solid tumors with performance characteristics characterized across >30,000 consecutively submitted FFPE tumor tissue samples[1,3]. Compared to the previous version, the current version targets 59 fusion driver genes (vs. 46 previously), resulting in a total of 437 unique genes across the CGP test. Additionally, the updated version leverages an independently trained and validated random forest classifier to detect gene fusions, and an

independently trained and validated random forest classifier to detect mutations (with equivalent or better performance to the previous version for all variant classes). The TMB component of the CGP+qTP test has been extensively validated[1] and has not been updated. Briefly, the TMB assessment for the CGP + qTP LDT (and IRS component) includes non-coding (at well characterized genomic loci) and coding, synonymous and non- synonymous, SNV and multi-nucleotide (two bases) variants from a panel with a maximum footprint of 1.7 Mb. Notably, only mutations with VAF > ¼ of the final molecularly informed tumor content are included in the TMB estimate (inclusion of only clonal mutations and tumor content correction have both been shown to improve prediction of checkpoint inhibitor response [4,5]). Additional custom filtering is employed to exclude high likelihood technical artifacts and germline variants and the TMB (Muts/Mb) estimate is calculated via the total number of positions with sufficient depth of coverage necessary for definitive assessment (maximum possible 1.7Mb).

The qTP component of the CGP + qTP test is completed using the same aliquot of RNA and multiplex PCR panel used in StrataNGS (for gene fusions), with the current version of the panel containing 950 amplicons targeting individual gene fusion isoforms involving 59 driver genes [chimeric amplicons; reported as CGP variants as described above], and 106 non-chimeric amplicons (103 target genes and 3 housekeeping genes) used for quantitative expression profiling; pre-clinical and investigational versions of the quantitative expression profiling component have included 26, 46 and 103 target genes. Target gene expression is determined in scaled, $\log_2$, median-centered units of normalized reads per million [nRPM][6], representing target gene expression normalized to housekeeping gene expression, then scaled to the distribution observed in a common control (normal FFPE genome in a bottle Ashkenazi father cell line [GIAB; GM24149 from Horizon Discovery]) per panel. For reporting, results are then scaled to a fixed pan-cancer median value of 10 after $\log_2$ normalization. Hence, all individually reported targets have a median value of 10.0 across all pan-solid tumor samples tested, with each unit increase representing a doubling from the median.

VALIDATION OF QTP AND IRS HOUSEKEEPING GENE SELECTION

To evaluate the suitability of 6 positive "control" genes included in initial pre-clinical versions of the qTP panel and identify novel housekeeping genes, we downloaded uniformly realigned, gene expression quantified, quantile normalized, and batch effect removed TCGA expression data (in fragments per kilobase per million [FPKM]) from[7]. Correlation of variation (CV) was determined for each gene per tumor type, and candidate housekeeping genes were ranked by the number of tumor types in which they ranked in the top 20 most stable genes (by lowest CV). Uniformly processed gene expression data (in transcripts per million [TPM]) from the highest-ranking housekeeping genes, the 6 pre-clinical "positive control genes", as well as the commonly used housekeeping gene *GAPDH* were then downloaded for 20,841 total samples contained in the MiPanda database[8] and CV for each gene was determined across all samples (cell line, normal and TCGA) in MiPanda and used to prioritize genes for inclusion in panels as potential housekeeping genes.

VALIDATION OF QTP AND IRS QUALITY CONTROL

As the qTP component of the CGP + qTP test was run in parallel to StrataNGS CGP clinical testing, quality control metrics (and several validation analyses) which leveraged a consecutive 4-month period of StrataNGS clinical testing using the final version of the qTP panel for the integrated CGP + qTP test (Oct 2021 to Jan 2022; n=3,904 total FFPE tumor tissue samples). In addition to a minimum total mapped read count, previous clinical versions of the qTP component modeled the expected distribution of housekeeping gene expression (Mahalanobis distance of sample housekeeping target expression < 4 standard deviations away from the population as passing QC) and confirmed that each housekeeping gene is within its LOQ (equivalent to reportable range given this includes both upper and lower bounds)[9]. Based on pre-validation experiments, we compared the correlation of panel-wide normalized target gene expression in replicate RNA aliquots from clinical FFPE tumor samples with reportable expression from the previous panel (using the previous total mapped read and housekeeping QC metrics) to those from the current qTP panel using a single metric of >150,000 total mapped reads as having reportable expression. Panel wide concordance correlation of >0.8 was considered acceptable. Additional QC is performed

through inclusion of the FFPE GIAB sample in all clinical runs of the gene expression panel subjected to the same approach. The correlation of target gene expression from replicate PD-L1, PD-1 and ADAM12 amplicons was determined across the 24,463 Strata Trial samples with complete sample information (including current tumor type reporting), informative TMB, informative gene expression, and tumor content ≥ 20% (regardless of treatment data availability).

VALIDATION OF QTP AND IRS: LIMIT OF QUANTIFICATION (LOQ), LINEARITY AND REPORTABLE RANGE

We determined LOQ and linearity for individual target genes by determining the lowest nRPM that can be precisely quantified in replicate RNA aliquots subjected to repeat multiplex PCR based library preparation (followed by templating and sequencing). Importantly, independent library preparations represent independent RNA aliquots, cDNA (from reverse transcription) and library preparation, so the nRPM level below which repeat samples show increased dispersion represents the lowest amount of RNA library that can be precisely quantified. We determined the LOQ for normalized expression of all individual target gene amplicons by evaluating the weighted root mean square error (WRMSE) using a 40 sample window (beginning with the highest expressing target gene expression in replicate #1) and evaluated the minimum RWSE, as well as additional windows (again beginning with the highest expressing target gene expression in replicate #1) where the WRMSE is first observed to be below the 50th, 25th, 15th and 10th quantile. Using this approach, the clinical implications of residual error distribution were weighed vs. the overall linearity and dynamic range of quantification in setting the most appropriate LOQ for each target gene amplicon. Linearity was thus determined by the concordance correlation coefficient for each target gene after setting all sub-LOQ values to LOQ, and the dynamic range was defined as the LOQ to the highest expressing value for that gene in replicate #1. No upper LOQ is established as there is essentially no chance of clinical misinterpretation of a value higher than that established in this approach given the observed linearity. Hence, the reportable range for each amplicon is floored at the LOQ but has no upper limit. Linearity LOQs have not been applied to any other validation analysis performed herein (unless specified) to present the full range of qTP generated data.

As a Cox proportional hazard-based algorithm, IRS produces a quantitative hazard that is proportional to the hazard rate observed in the dataset, so that higher values represent decreased hazard (i.e., more benefit from PD-(L)1 therapy benefit) and smaller values represent increased hazard (i.e., less benefit from PD-(L)1 therapy). The individual scaled hazard rate is reported for informational purposes however the IRS result is reported categorically as IRS-High or IRS-Low. Hence, for the IRS test result, measurand LOD/LOQ and linearity is not applicable, and the reportable range of the quantitative scaled hazard rate is reported as determined without upper or lower bounds. Performance of the IRS model with or without LOQs applied to the individual expression components was compared and concordance correlation of IRS scores and quantification of the % of patients changing IRS groups (IRS-H to IRS-L or vice versa) was determined.

VALIDATION OF QTP AND IRS ACCURACY

The accuracy of the qTP component was validated through a multi-part accuracy study leveraging qRT-PCR, comparison to known target gene expression across tumor types, and clinical immunohistochemistry. For representational qRT-PCR validation, clinical FFPE tumor samples from StrataNGS testing and 3 control RNA samples were subjected to qTP and qRT-PCR on replicate RNA aliquots. For qRT-PCR, 2-20ng clinically isolated FFPE RNA per sample underwent reverse transcription using SuperScript IV VILO Master Mix (Invitrogen) and pre-amplification using TaqMan PreAmp Master Mix (Applied Biosystems) using a pooled Taqman primer/hydrolysis probe assays and 14 cycles. qPCR was then performed in duplicate on a Quantstudio 3 Real Time PCR system using a 1:20 dilution of amplified product per qPCR reaction and TaqMan Fast Universal PCR Master Mix (Applied Biosystems). Individual amplicon level thresholds and baselines were set during the exponential amplification phase to determine cycle crossing threshold (C_t) values. Samples with duplicate qPCR values > 2 C_t difference were excluded unless both values were >30 or singlicate experiments were performed. All undefined C_t values were considered as having C_t of 40. qRT-PCR ΔC_t values were determined as: target amplicon C_t – (median of housekeeping gene amplicon C_t) and were otherwise scaled as for qTP results using the same FFPE reference sample run in all clinical and validation runs.

Panel-wide concordance correlation coefficient were determined across all included target genes and samples in the cohort. Acceptable concordance correlation coefficient of >0.7 was pre-specified given the expected range of transcript expression across the amplicons/samples.

To indirectly compare qTP generated target gene expression to that expected in a given tumor type, we compared target gene expression profiles for all 103 target genes from qTP ($\log_2$) to the pan-cancer TCGA tumor set[10] (RSEM batch normalized log $[n+1]_2$, from Illumina HiSeq_RNASeqV2; downloaded from cBioPortal) consisting of 9,618 samples from 30 TCGA tumor types that could be mapped directly to 28 Strata defined primary and/or secondary tumor types across the 4-month period of clinical StrataNGS testing described above. Results were summarized across the entire 103 target gene set per tumor type, and correlation (Spearman rho) of per-target gene mean expression in each dataset was determined; no acceptance criteria were pre-specified. As IRS was trained on data from both the current qTP panel and the previous 46 gene version (with appropriate panel-specific scaling), we also compared TCGA and qTP results for the 20 immune and proliferation expression candidates included in IRS development (*IFNG* was excluded from this analysis as it could not be reliably quantified across all qTP panels) across the 24,463 Strata Trial samples with complete sample information used to assess IRS distribution (see **Figure 5**). Spearman correlation was determined between TCGA and qTP profiled tumors for all candidate biomarkers.

To validate qTP and IRS components vs IHC, we used optical character recognition and natural language processing to prioritize accompanying pathology reports received with StrataNGS test requests for abstraction of IHC biomarker results by trained reviewers according to a documented SOP into a clinical database. Where orthogonal IHC biomarkers and StrataNGS were performed on different specimens from the same case (pathology accession) that are presumed to come from the same tumor (e.g., single case with a primary colon cancer, lymph node metastasis, liver metastasis resection), testing was considered as performed on the same specimen and suitable for comparison (representing usual clinical practice). Where orthogonal biomarker and StrataNGS testing were performed on different specimen from the same case grossly or histologically distinct tumors were described and commented on in the specimen report,

testing was considered as performed on distinct specimens and not suitable for comparison. For Ki67 and PD-L1, orthogonal results were excluded if a range of IHC expression >20% was provided (e.g., Ki67 staining reported as >50% were excluded); for orthogonal IHC results with ≤20% range, the average of the range was used (e.g., Ki67 >80% = 90%). All analyses used samples with reportable qTP and tumor content ≥20%.

As the CGP + qTP test reports a composite proliferation biomarker (*TOP2A+UBE2C* expression), and previous analyses using microarray expression profiling and/or research grade multiplex PCR RNAseq have shown that TOP2A and UBE2C are robust cell cycle/proliferation biomarkers[11-14], the entire available cohort (n=956) was used to establish accuracy of this proliferation biomarker by determining the correlation coefficient with clinical IHC proliferation index by percent Ki67 positive (with acceptable correlation coefficient of >0.7 pre-specified). Hence no acceptable correlation coefficient was prespecified for the analysis of *TOP2A* only (the biomarker included in the IRS model) vs. IHC proliferation index).

Likewise, the CGP + qTP test reports *PD-L1* individually through averaging target gene expression from the two *PD-L1* amplicons. Hence, we determined the accuracy of our qTP *PD-L1* quantification vs. clinical IHC using 256 NSCLC FFPE tumor samples with clinical PD-L1 IHC expression by the 22C3 clone (using tumor proportion score [TPS]) in accompanying pathology reports. As TPS does not include PD-L1 expression in non-tumor cells (as for CPS using 22C3 in other tumor types and routinely included in PD-L1 expression by other PD-L1 IHC clones [e.g., SP142], acceptable accuracy was pre-specified as statistically significant, ordinally increasing differences in median qTP *PD-L1* expression between the three clinically relevant TPS groups (Kruskal Wallis test, $p<0.05$; Jonckheere-Terpstra trend test [increasing median from 0%, 1-49%, and ≥50%, $p<0.05$). As the *PD-L1* component of IRS only uses target gene expression from a single *PD-L1* amplicon (CD274.E4E5.NM_014143.3) based on the Lasso penalized CPH modeling, no acceptable correlation coefficient was prespecified for the analysis of target gene expression from only this *PD-L1* amplicon vs. TPS IHC.

VALIDATION OF QTP AND IRS TUMOR CONTENT LIMIT OF DETECTION (LOD)

Tumor content LOD was determined by binning the development and validation cohort samples by tumor content (20-35%, 40-70% and >70%) and visualizing TTNT by Kaplan Meier analysis, given that the established LOD for accurate TMB estimation was determined as 20% tumor content, and included a tumor content term (continuous tumor content) in the overall adjusted CPH model in the IRS development cohort (including age, gender, most common tumor type [NSCLC] vs. others, therapy type [monotherapy/combination], and line of therapy) and validation cohort (including age, gender, most common tumor type [melanoma] vs. others, therapy type [PD-1 vs. PD-L1], and line of therapy). Additionally, we identified subjects in the SCMD that otherwise would have been included in the discovery or validation cohorts, but the tumor content of the tested sample was <20%, and determined the performance of IRS by CPH modeling (adjusting for age, gender, therapy line, most common tumor type (NSCLC) vs. others, PD-(L)1 type (pembrolizumab vs. other PD-[L]1), and monotherapy vs. combination therapy (for pembrolizumab).

VALIDATION OF QTP AND IRS REPRODUCIBILITY

Formal reproducibility of the gene expression panel used for the CGP + qTP test and the IRS algorithm was established separately from the previous validation of TMB reproducibility[1], as the current version of the qTP panel was not performed in parallel with the initial TMB reproducibility experiments[15]. Hence, panel-wide qTP and IRS reproducibility between operators, lots, and instrumentation was established using separate replicate nucleic acid aliquots isolated from FFPE tumor samples. Twenty-seven unique samples were assessed by two operators on different days using different library preparation instrumentation, different library preparation reagent lots, and different templating and sequencing lots. Each operator performed templating and sequencing sequentially for each run on different Ion Chefs and on different S5XL or S5 Prime sequencing instruments. For each sample, the maximum and minimum nRPM for each target gene across all replicates was plotted. Overall panel-wide acceptable concordance correlation coefficient was set at 0.8. Concordance correlation of maximum and minimum IRS score for each sample was also determined. Qualitative agreement of IRS status (High vs. Low) from

the maximum and minimum IRS score across all replicates was determined. Acceptable IRS score concordance correlation coefficient was pre-specified at 0.8 and acceptable qualitative IRS agreement accuracy was set at >90%.

CLINICAL VALIDITY AND CLINICAL UTILITY OF QTP IN PATIENTS WITH ADVANCED BREAST CANCER

For orthogonal validation of breast cancer biomarkers, identification of StrataNGS tested cases with estrogen receptor (ER; ESR1), progesterone receptor (PR; PGR), or ERBB2 (HER2) was performed as described above for Ki67 and PD-L1. Cases were excluded if they only referenced results from a previous sample, and only cases with quantitative results were included; ER and PR reported as <1% were considered negative (0% staining). HER2 IHC expression was binned into the four currently distinct clinical groups (0, 1+, 2+, and 3+). As ER, PR and ERBB2 expression by clinical IHC have established clinical utility in therapy selection for patients with advanced breast cancer, we performed a similar accuracy analysis vs. clinical IHC to that described above for the *TOP2A* and *PD-L1* components of IRS, however cohorts were randomly split into training and validation cohorts (for clinical validity analysis after setting thresholds in the training cohort as described below) before performing the accuracy analyses. *ER* and *PR* have only been assessed on the current qTP panel, while *HER2* has been assessed on previous versions.

Accuracy of *ER* by qTP was validated against clinical IHC using a cohort of 300 breast FFPE tumor samples with reportable qTP (including tumor content [TC] ≥ 20%) and ER IHC expression (by % tumor cells positive) in accompanying pathology reports. The entire cohort was used for accuracy, however the cohort was randomly split into equivalent training (n=150) and validation (n=150) cohorts to establish clinical validity prior to performing the accuracy assessment. Accuracy of *PR* by qTP was performed similarly using a cohort of 291 breast cancer samples with reportable qTP and PR IHC (training cohort, n=145; validation cohort, n=146). Accuracy of *HER2* by qTP was performed similarly using a cohort of 545 breast cancer samples with reportable qTP and HER2 IHC (training cohort, n=273; validation cohort,

n=272). For *ER* and *PR*, acceptable Pearson correlation coefficient of >0.7 was pre-specified. Given current semi-quantitative clinical HER2 IHC reporting, acceptable concordance was pre-specified as statistically significant, ordinally increasing differences in median qTP *ERBB2* expression between each of the four groups (Kruskal Wallis test, $p<0.05$; Jonckheere-Terpstra trend test [increasing median from 0 to 1+ to 2+ to 3+], $p<0.05$).

Clinical validity for ER status by qTP was established by setting thresholds for qTP ER Negative (<12.75) and Positive (>14.5) in the training cohort (n=150) of breast cancer FFPE tissue samples with clinical ER status (by % tumor cells positive) based on the clinical IHC defined categories of ER Negative (0%), Low (1-10%) and Positive (>10%). Expression between the Negative and Positive thresholds were defined as qTP ER inconclusive. Desired sensitivity (positive percent agreement [PPA]) and specificity (negative percent agreement [NPA]) for qTP *ER* Negative/Positive status (vs. IHC Negative and Positive) was pre-specified as >95% each. Locked thresholds were then applied to the validation cohort (n=150). Clinical validity for PR status by qTP was established by setting a threshold for qTP PR Negative (<12.3) in the training cohort (n=145) of breast cancer FFPE tissue samples with clinical PR status (by % tumor cells positive). Although PR does not have a “Low” clinical IHC reporting group, three clinical IHC defined categories of PR Negative (Neg.; 0%), Low (1-10%) and Positive (Pos; >10%) were used in the training cohort to facilitate appropriate balancing of PPA and NPA in the threshold setting. As the potential clinical implications of false positive PR status, namely inappropriately considering an ER negative / HER2 negative breast cancer as hormone receptor positive (vs. triple negative) are more impactful than false negative PR status (it is unclear if ER negative/PR positive breast cancer are biologically plausible), the threshold was set to favoring NPA and pre-specified acceptable NPA (versus PR 0% IHC) of greater than 95% was set. The locked threshold was then applied to the validation cohort (n=146). Clinical validity for HER2 status by qTP was established by setting thresholds in the training cohort (n=273) of breast cancer FFPE tissue samples with clinical HER2 status (0, 1+, 2+ or 3+ categories) in accompanying pathology reports. As with ER, given that the clinical utility of HER2 IHC 2+ is to reflex to FISH/ISH (and StrataNGS provides *ERBB2* copy status), and the unclear validity of

0 vs. 1+ expression in retrospective samples clinically scored before the FDA approval of trastuzumab deruxtecan in HER2 1+ and 2+ (FISH/ISH negative) breast cancer, we set thresholds for qTP HER2 Low (<18.0) and High (>19.2), with expression in between those thresholds reported as qTP HER2 Inconclusive (light gray); the threshold was set by balancing desired maximum sensitivity vs. IHC 3+ with the observation that the majority of IHC 3+ tumors with the lowest qTP *HER2* expression also lacked *ERBB2* amplifications in the training cohort. Hence, desired NPA and PPA for qTP HER2 Low/High status (vs. IHC Negative and 3+) was pre-specified as NPA >95% and PPA > 70%; no performance metrics for IHC 2+ samples were prespecified. Locked thresholds were then applied to the validation cohort (n=272 [including 51 IHC 2+ not formally evaluated]).

As ER/PR/HER2 IHC results may not be available in pathology reports submitted for CGP or may be inconclusive, we determined the impact of integrating qTP results with CGP results given that standard of care *PIK3CA* mutations are associated with FDA-approved alpelisib + fulvestrant therapy only in patients with hormone receptor positive/HER2 negative breast cancer. Hence, across the 4-month period of consecutively tested pan-solid FFPE tumor samples (n=3,904) submitted for clinical CGP testing described above, we identified those that were breast cancer and met qTP and CGP QC metrics (including the final ≥20% tumor content requirement) needed to evaluate HR status, *PIK3CA* mutations and *ERBB2* copy number status. For all cases with standard of care *PIK3CA* mutations, we stratified results by hormone receptor (HR) status by qTP (qTP ER and PR Negative as HR Negative) and *ERBB2* amplification status by StrataNGS CGP testing.

SUPPLEMENTARY RESULTS

CLINICAL AND MOLECULAR ANALYSES SUPPORTING THE VALIDITY OF THE STRATA CLINICAL MOLECULAR DATABASE (SCMD)

Among the 9,899 patients in the SCMD having treatment data from at least one systemic antineoplastic agent, the median follow-up from start of first systemic treatment was 15.4 months [interquartile range (IQR) 6.9-29.4 months]. The median number of total systemic lines of therapy per patient was 1 (IQR 1-2), with 49.2% of systemic lines being monotherapy, and the median number of systemic therapies per line was 2 (IQR 1-2). The median number of total systemic lines of therapy per patient after Strata trial enrollment was 1 (IQR 0-1), with 47.2% of systemic lines being monotherapy, and the median number of systemic therapies per line was 2 (IQR 1-2). As expected, median rwPFS was shorter in subsequent therapy lines (median rwPFS in 1st, 2nd and 3^{rd+} lines of 9.4 [95% CI 9.1-9.7], 8.7 [95% CI 8.4-9.1] and 7.1 [95% CI 6.7-7.4] months, respectively, unadjusted log rank $p<0.0001$; **Supplementary Figure S3**).

We have previously demonstrated that molecular alteration frequency in the first ~30,000 consecutive patients enrolled in the Strata Trial[3] was similar to that observed in the Memorial Sloan Kettering single institution pan-cancer profiling effort, MSK-IMPACT[16], supporting the generalizability of the SCMD. Given the substantial proportion of patients in the SCMD with NSCLC and extensive previous characterization of molecular subtypes and associated therapies, we leveraged the NSCLC cohort to assess the clinical validity of using the SCMD to support this study. Of the 9,899 Strata Trial patients, 1,416 (14.3%) had NSCLC, with 157 patients with NSCLC receiving a first line systemic NSCLC targeted oncogene targeted monotherapy against *EGFR*, *ALK*, *ROS1* or *MET* (regardless of whether currently preferred) , those receiving a second generation or later inhibitor showed significantly longer rwPFS (by TTNT) vs. patients receiving first-generation (erlotinib, gefitinib or crizotinib) inhibitor (later [n=120] vs. first generation inhibitor [n= 37], median rwPFS 25.3 [20.3-33.6] vs. 11.0 [95% CI 8.7-21.7] months; adjusted hazards ratio for later vs. first-generation inhibitor 0.44 [95% CI 0.27-0.73], p=0.001, **Supplementary Figure S4a**. Importantly, in the 129 patients with NSCLC receiving a current first-line

systemic targeted oncogene monotherapy, whether the treatment occurred before (treatment decision made from orthogonal testing, n=57) or after (n=72) receiving StrataNGS test results was not a significant predictor of rwPFS (before vs. after StrataNGS results, median rwPFS 22.8 [95% CI 17.3-31.8] months vs. 29.0 [95% CI 20.3-29.0] months; adjusted hazards ratio 1.46 [95% CI 0.77-2.79], p=0.25; **Supplementary Figure S4b**). Lastly, given the challenging nature of specimens received for CGP in our real-world experience, we routinely run samples not meeting our input sample characteristics and perform molecular pathologist review in all cases to "rescue" variants from samples not meeting usual CGP QC metrics from one or more variant classes. Hence, although limited in number, we compared rwPFS in SCMD NSCLC patients treated with a biomarker matched, first line oncogene NCCN preferred targeted monotherapy TKI after receiving StrataNGS results based on whether the sample 1) passed both StrataNGS sample input criteria and relevant sequencing QC metrics (see **Supplementary Methods**) or 2) did not meet sample input or sequencing QC metrics but reported a therapy matched biomarker. Samples failing sample and/or sequencing QC metrics (n=17) vs. meeting all QC metrics (n=48) was not a significant predictor of rwPFS (failing vs. meeting QC, median rwPFS 17.1 [95% CI 10.0-17.1] vs 29.0 [95% CI 20.3-29.0] months; adjusted hazards ratio 1.72 [95% CI 0.49-5.99], p=0.39; **Supplementary Figure S4c**).

VALIDITY OF TMB BY STRATANGS TESTING

As shown in **Supplementary Figure S6**, to confirm the validity of ≥10 Muts/Mb from StrataNGS testing to define TMB-H, we demonstrated that TMB-H patients (*n* = 130) had significantly longer pembrolizumab monotherapy rwPFS vs. TMB-L patients (*n* = 291; median rwPFS Not reached [95% CI 16.6 - NA] vs. 7.2 [95% CI 6.0 – 10.7] months, adjusted hazard ratio 0.37 [95% CI 0.25 – 0.54], p<0.0001 when adjusted for age, gender, most common tumor type [NSCLC] vs. others, and line of systemic therapy **Supplementary Figure S6a**), as well as significantly longer OS (median OS Not reached [95% CI NA - NA] vs. 16.7 [95% CI 13.2 – 22.9] months, adjusted hazard ratio 0.44 [95% CI 0.29-0.67], p=0.0001; **Supplementary Figure S6b**).

APPROPRIATENESS OF REAL-WORLD PROGRESSION-FREE SURVIVAL (RWPFS) FOR STUDYING PEMBROLIZUMAB TREATMENT OUTCOMES

To establish the appropriateness of real-world progression free survival (rwPFS; by time to next therapy) for studying PD-(L)1 treatment outcomes, rwPFS was compared to overall survival (OS) in the discovery cohort. As shown in **Supplementary Figure S5**, the overall correlation (Spearman ρ= 0.58) was impacted by several outliers. Of the four patients with the greatest difference in months of OS vs. TTNT, the first was a patient with metastatic NSCLC harboring an *EML4-ALK* fusion by StrataNGS testing who was briefly treated with pembrolizumab and chemotherapy before prolonged treatment with crizotinib and lorlatinib (**Supplementary Figure S5 red box**), the second was a patient with metastatic melanoma who was briefly treated with pembrolizumab, then ipilimumab + nivolumab, prior to an extended course with imatinib (the patient harbored two VUS in *KIT;* **Supplementary Figure S5 blue box**), the third was a patient with metastatic melanoma who was treated with pembrolizumab prior to prolonged treatment with ipilumumab monotherapy (**Supplementary Figure S5 green box**), and the fourth was a patient with metastatic NSCLC harboring EGFR p.G719C and p.S768I mutations who was briefly treated with pembrolizumab and chemotherapy before prolonged treatment with osimertinb followed by pembrolizumab and chemotherapy (**Supplementary Figure S5 purple box**). Taken together, although correlated, these results support evaluation of both TTNT and OS herein, as well as the real-world importance of identifying patients with oncogenic alterations who may be more appropriately treated with targeted therapy vs. PD-(L)1 therapy.

VALIDATION OF QTP AND IRS: INTRODUCTION

The qTP component of the CGP + qTP test used to report IRS is performed from the same aliquot of RNA and multiplex PCR panel used in StrataNGS (for gene fusions), with the current version targeting 106 non-chimeric amplicons (103 target genes and 3 housekeeping genes) used for quantitative

expression profiling. Target gene expression is determined in scaled, log_2, median-centered units of normalized reads per million [nRPM][17,18], representing target gene expression normalized to housekeeping gene expression (from *HMBS*, *CIAO1* and *EIF2B1*; see below), then scaled to the distribution observed in a common FFPE cell line control per panel. All 106 amplicons (median insert length 94.5, range 61-120) use primers (median primer length 23, range 10-31) that span exon-exon boundaries and only full length reads (<2 base-level mismatches) are counted, ensuring specificity of all normalized target gene values from each amplicon. Beyond IRS, the qTP panel includes amplicons for quality control (candidate housekeeping genes, separate amplicons targeting different regions the same transcript) and multiple classes of target gene expression biomarkers, including those potentially useful for determining hormone receptor status (see below), those measuring proliferation index, and individual biomarkers that may have utility in predicting response (or clinical trial suitability/enrollment,) to investigational or approved expression based therapies (e.g., antibodies, antibody drug conjugates [ADCs], bispecific antibodies, radiopharmaceuticals, CAR-T cells, TCRs, etc.).

To be clinically useful, qTP (and derived integrated biomarkers such as IRS), must provide precise, quantitative results that can be used to predict therapeutic efficacy.[17,18] As described in the validation of Omniseq—a New York state approved multiplex RT-PCR NGS assay—"standard measures that are routinely used to describe the analytical performance of variant detection assays (such as sensitivity and specificity) are not equally applicable to GEX [gene expression] by RNAseq, and well characterized reference standards for quantitative measurement of transcript levels are currently lacking".[17,18] Likewise, approaches used in validating assay linearity and quantitative bias in multi-gene expression assays not performed in multiplex (e.g., proportionality of threshold cycle [Ct] values vs. input RNA concentration[17,18]), cannot be used directly in the validation of a multiplex RNAseq based-test. Hence, qTP panel-wide validation analyses applicable to IRS, as well as validation of the IRS biomarker, are described herein (see **Supplementary Figure S17** for validation of breast cancer related biomarkers).

VALIDATION OF QTP AND IRS: HOUSEKEEPING GENE VALIDATION

Accurate quantitative gene expression by multiplex PCR based RNAseq requires appropriate housekeeping genes for target gene normalization; importantly, unlike approaches only applicable to a single tumor type (e.g., predictive gene expression assays for breast cancer) or only assessing a single class of biomarkers (e.g. immune cell and tumor microenvironment characterization), our approach required pan-tumor and pan-normal stable housekeeping genes given the inclusion of both tumor (e.g., *ERBB2*, *ES1R*, *PGR*) and non-tumor (e.g., *PDCD1*) components and the need to be robust to variable tumor content across tissues. Initial pre-clinical versions of the qTP panel contained 6 "positive control" genes across two RNA primer pools previously used in the RNA fusion component of the Oncomine Focus/Precision Assay. To evaluate the suitability of these markers as pan-cancer housekeeping genes for quantitative expression profiling and/or identify other candidates, we performed a multi-part evaluation of transcriptome profiles of pan-cancer, pan-normal tissue stability >20,000 tumor, normal and cancer cell line samples as shown in **Supplementary Figure S7a**. First, uniformly realigned, gene expression quantified, quantile normalized, and batch effect removed TCGA expression data (in fragments per kilobase per million [FPKM]) was downloaded for 6,875 tumor samples (from 18 tumor types) from[7]. Correlation of variation (CV) was determined for each gene per tumor type, and candidate housekeeping genes were ranked by the number of tumor types in which they ranked in the top 20 most stable genes (by lowest CV). Uniformly processed gene expression data (in transcripts per million [TPM]) from the 18 highest ranking housekeeping genes, the 6 OPA "positive control genes", as well as the commonly used housekeeping gene GAPDH were then downloaded for 20,841 total samples contained in the MiPanda database[8], which includes 935 Cancer Cell Line Encyclopedia cell lines (from 20 tumor types), 9,966 normal tissue samples (730 TCGA samples from 20 tissue types and 9,236 GTEX samples from 30 tissue types), and 9,940 TCGA cancer tissue samples (9,496 primary samples from 25 tumor types and 444 metastases from 16 tumor types). Given that most target genes are expressed at much lower levels than typical control genes (such as *GAPDH*), we prioritized inclusion of pan-cancer, pan-normal tissue stable genes with the lowest average expression (in TPM) as candidate housekeeping genes for the qTP component of the CGP + qTP test thus selected five genes for inclusion: *SLC4A1AP*, *CTCF*, *EIF2B1*, *CIAO1* and *GGNBP2*.

Three candidate housekeeping genes, *EIF2B1*, *CIAO1* and *HMBS*, had the most stable expression patterns and widest LOQ range across clinical FFPE tumor samples in previous panel versions, and these three housekeeping genes are used in the validated version of the qTP panel defined herein, with target gene expression normalized to the expression of the median housekeeping gene. Normalized expression for these three housekeeping genes and the other candidates on the current version of the qTP panel are shown from the consecutive 4-month period of StrataNGS clinical testing described above (n=3,417 samples with reportable qTP, regardless of tumor content) in **Supplementary Figure S7b**, supporting the pan-tumor stability of the housekeeping genes used in the quantitative expression component of the qTP panel and IRS.

VALIDATION OF QTP AND IRS: EXPRESSION QC METRIC VALIDATION

Previous clinical versions of the expression component modeled the expected distribution of housekeeping gene expression and confirmed that each housekeeping gene is within its LOQ[9]; however, by comparing qTP panel wide, normalized target gene expression from 394 FFPE clinical tumor samples processed on the current version of the qTP panel validated herein, we observed highly correlated expression in all samples with >150,000 total mapped reads in the current version regardless of housekeeping gene distribution or LOQ status in the previous version (correlation coefficient = 0.942 [95% CI 0.941-0.944], p-value <0.0001); in 129 samples with <150,000 mapped reads in the current qTP version, correlation coefficient vs. the previous version was only 0.649 (95% CI 0.639-0.659), supporting the use of this QC metric to determine reportability of individual qTP biomarkers and IRS. Importantly, through this series of analyses, we have demonstrated that each sample can serve as its own internal control to confirm suitability for quantitative gene expression.

Unlike traditional RNAseq, where overall gene quantification values (e.g., in FPKM) are reported and are dependent on alignment approaches, our multiplex PCR based RNAseq approach enables unambiguous read assignment to each target gene amplicon. As described above, two separate amplicons targeting different exon-exon junctions of the same gene are present in the qTP panel for several clinically relevant genes, including *PD-L1*, *PD-1* and *ADAM12* in the current qTP panel (only one of two *ADAM12*

amplicons was also present on all previous qTP panels used in IRS validation). Hence, we determined the correlation of replicate *PD-L1*, *PD-1* and *ADAM12* amplicons across the 24,463 Strata Trial clinical samples with complete sample information used to assess IRS distribution (see **Figure 5**). As shown in **Supplementary Figure S7d-f**, across the 24,463 samples (n=7,911 samples on panels with both *ADAM12* amplicons), observed correlation coefficients for *PD-L1*, *PD-1* and *ADAM12* were 0.863 (95% CI 0.859-0.866; $p<0.0001$), 0.811 (95% CI 0.807-0.815; $p<0.0001$) and 0.908 (95% CI 0.904-0.912; $p<0.0001$), respectively.

VALIDATION OF QTP AND IRS: LIMIT OF QUANTIFICATION (LOQ), LINEARITY AND REPORTABLE RANGE

Limit of quantification (LOQ) and linearity experiments are complicated in multiplex RNA sequencing due to the lack of absolute standards at the extremes of expression that can be assessed in a complex RNA mixture with variable RNA composition and amplification efficiency. Hence, we determined LOQ and linearity for individual qTP target gene expression by determining the lowest nRPM that can be precisely quantified in replicate RNA aliquots from 619 clinical FFPE tumor samples subjected to repeat qTP, as the nRPM level below which repeat samples show increased dispersion represents the lowest amount of RNA library that can be precisely quantified. Across the 103 target gene amplicons, median linearity by concordance correlation coefficient of normalized target gene expression was 0.88 (range 0.50 to 0.99) across a median dynamic range (lower LOQ to highest replicate sample) of 252 fold (range 13-92,682 fold). For IRS, the individual scaled hazard rate is reported for informational purposes, however the IRS result is categorical as IRS-High or IRS-Low. As a Cox proportional hazard-based algorithm, IRS measurand LOD/LOQ and linearity is not applicable, and the reportable range of the quantitative scaled hazard rate is reported as determined without upper or lower bounds. Performance of the IRS model with or without LOQs applied to the individual expression components was compared and concordance correlation of IRS scores and quantification of the % of patients changing IRS groups (IRS-H to IRS-L or vice versa) was determined.

VALIDATION OF QTP AND IRS ACCURACY INTRODUCTION

As described above, sensitivity/specificity/accuracy and linearity/LOQ are complicated in multiplex RNA sequencing due to the lack of absolute standards that can be assessed in a complex RNA mixture with variable RNA amplifiability, individual amplicon efficiency, and the difficulty in appropriately choosing a diluent (water to reduce input RNA amount, normal DNA for genomic alterations, or "normal" RNA to reduce relative amount of highly expressed tumor-specific transcripts are all inappropriate for a multiplex RNAseq pan-tumor approach targeting tumor and tumor microenvironment targets). Hence, the accuracy of the qTP panel and IRS expression components was validated through a multi-part accuracy study leveraging representational qRT-PCR validation, comparison to known target gene expression across tumor types (via comparison to TCGA expression data), and clinical IHC.

VALIDATION OF QTP AND IRS ACCURACY BY QRT-PCR

The accuracy of the qTP component of the integrate CGP + qTP test was first determined by representational validation through evaluating target gene expression concordance with hydrolysis probe based qRT-PCR, the gold standard for gene-expression measurement.[19] Clinical FFPE tumor samples from StrataNGS testing and 3 control RNA samples were subjected to qTP and qRT-PCR on replicate RNA aliquots. Comparison of target gene expression by qTP and qRT-PCR was performed for 32 target genes in 3 control samples and 24 clinical FFPE tumor samples (analytical validation) and the 24 FFPE tumor samples only (clinical validation). In the analytical validation, the observed concordance correlation coefficient was 0.837 (95% CI 0.816-856; p<0.0001). As shown in **Supplementary Figure S7c**, in the clinical validation, the observed concordance correlation coefficient was 0.842 (95% CI 0.820-862; p<0.0001); Likewise, when limited to just the four target genes comprising the expression component of IRS (*PD-1*, *PD-L1*, *TOP2A*, and *ADAM12*), the concordance correlation coefficient in the clinical validation was 0.833 (95% CI 0.760-0.886). Taken together, these results demonstrate the highly quantitative nature of the qTP panel and the gene expression component of IRS.

VALIDATION OF QTP AND IRS ACCURACY VS. THE CANCER GENOME ATLAS (TCGA) PROFILED TUMORS

To further evaluate the accuracy of the entire qTP panel, we compared qTP results for all 103 target genes to established gene expression profiles from the pan-cancer TCGA tumor set. Although TCGA data is largely from localized tumors, StrataNGS and the qTP panel is performed on either localized (from patients who later developed advanced/metastatic disease) or metastatic tumor samples. Hence, although an indirect measure of overall accuracy, expression profiles would be expected to be robust both across tumor types and transcriptional programs. As described in the methods, we compared Illumina HiSeq generated target gene expression profiles from the pan-cancer TCGA RNAseq tumor set, consisting of 9,618 samples from 30 TCGA tumor types that could be mapped directly to 28 Strata defined primary and/or secondary tumor types, to the 4-month period of clinical StrataNGS testing described above (3,222 FFPE tumor samples with reportable qTP and tumor content ≥20%). Target gene expression profiles were highly concordant across TCGA and qTP when stratified by tumor type (median Spearman correlation across 19 tumor types with at least 10 samples in each group = 0.897, range 0.838 to 0.925).

As the IRS model was trained using expression biomarkers present on both the current and 46 target gene qTP panels, we performed an expanded comparison of TCGA and qTP results for the immune and proliferation candidates included in IRS development across the 24,463 with complete sample information used to assess IRS distribution (see **Figure 5**). As shown in **Supplementary Table S2**, after limiting to 27 directly comparable tumor types, target gene expression per tumor type was compared for the 20 candidate expression biomarkers included in IRS development across 9,223 TCGA tumors and 18,305 qTP. Across all 20 candidates (*IFNG* was excluded from this analysis as it could not be reliably quantified across all versions of the quantitative expression profiling panel), the median Spearman correlation between TCGA and qTP tumors was 0.831 (range 0.624-0.938), while the median correlation of the four IRS components was 0.823, as *TOP2A*, *PD-L1*, *PD-1* and *ADAM12* showed correlations of 0.896, 0.831, 0.815, 0.707 (all $p<0.0001$), respectively.

VALIDATION OF QTP AND IRS ACCURACY VS. CLINICAL IMMUNOHISTOCHEMISTRY (IHC)

Although hydrolysis probe based qRT-PCR is the gold standard for RNA transcript quantification, IHC is the clinical gold standard for clinically relevant target expression evaluation. Therefore, we used optical character recognition and natural language processing to prioritize accompanying pathology reports received with StrataNGS test requests for abstraction of IHC biomarker results as described in the **Supplementary Methods**. Accuracy results relevant to IRS are described here, with results relevant to breast cancer biomarkers shown in **Supplementary Figure S17**.

Accuracy of the *PD-L1* qTP component of IRS was validated against clinical IHC using a cohort of 276 NSCLC FFPE tumor samples with reportable qTP and PD-L1 IHC expression by the 22C3 clone (using TPS) in accompanying pathology reports. As shown in **Supplementary Figure S8a**, PD-L1 expression by qTP showed ordinally increasing median expression across the clinically relevant TPS bins (0%, 1-49%, and ≥50%; Kruskal Wallis $p<0.0001$, Jonckheere-Terpstra trend test $p<0.0001$). Only 24 of these samples came from the 154 patients in the propensity matched first line NSCLC treatment analysis (see **Figure 4**), precluding direct assessment of IRS vs. PD-L1 IHC for predicting pembrolizumab benefit. However, IRS status could be generated for all 276 NSCLC samples in the PD-L1 IHC cohort, with 31.0%, 34.2% and 58.0% of TPS 0%, 1-49%, and ≥50% samples being IRS-H, respectively. Accuracy of the *TOP2A* qTP component of IRS was validated against clinical IHC using a cohort of 956 FFPE tumor tissue samples (36 tumor types) with reportable qTP with proliferation index (percentage of Ki67 positive tumor cells) in accompanying pathology reports. As shown in **Supplementary Figure S8b**, *TOP2A* by qTP was strongly correlated to Ki67 proliferative index (correlation coefficient 0.753 [95% CI 0.724-0.780], $p<0.0001$). These results further support the accuracy of the gene expression component of IRS.

VALIDATION OF QTP AND IRS REPRODUCIBILITY

Panel-wide qTP and IRS reproducibility between operators, lots, and instrumentation was established using separate replicate nucleic acid aliquots isolated from FFPE tumor samples. Twenty-seven unique samples were assessed by two operators on different days using different library preparation instrumentation, different library preparation reagent lots, and different templating and sequencing lots. As shown in Figure S8c, concordance correlation coefficient of panel-wide (n=103 target genes) maximum vs. minimum nRPM for each target gene across all replicates for the 27 samples was 0.950 (95% CI 0.946-0.953; p<0.0001). Similarly, concordance correlation of maximum vs. minimum IRS score for each sample was 0.978 (95% CI 0.952-0.990; p<0.0001) as shown in Figure S8d, with 100% concordance for IRS-H vs. -L status. Taken together, these results demonstrate the highly reproducible nature of qTP and the integrative IRS biomarker.

VALIDATION OF QTP AND IRS SUMMARY

All qTP and IRS validation analyses met pre-specified acceptance criteria. Additional data supporting accuracy vs. other IHC biomarkers, analyses supporting the 20% overall qTP tumor content requirement beyond IRS, analyses supporting the utility of the current QC metrics, and clinical utility and validity of biomarkers beyond IRS and breast cancer biomarkers will be reported separately. IRS robustness to sample collection timing (e.g. immediately prior to PD-(L)1 therapy vs. prior to a preceding systemic therapy) and tumor content (supporting the overall 20% tumor content requirement) are described below.

STRATIFICATION OF THE VALIDATION COHORT BY PD-1 VS. PD-L1 THERAPY

In the overall 248 patient non-pembrolizumab PD-(L)1 monotherapy validation cohort (**Figure 2c&d**), by Kaplan Meier analysis, IRS-H patients had significantly longer PD-(L)1 monotherapy rwPFS (IRS-H vs. IRS-L median TTNT 23.1 [95% CI 17.1-32.9] vs. 10.2 [95% CI 8.7-14.8] months, adjusted hazard ratio = 0.52 [95% CI 0.34-0.80], p=0.003) and OS (IRS-H vs. IRS-L median OS 40.4 [95% CI 32.9-NA] vs. 21.4 [95% CI 17.0-46.8] months, adjusted hazard ratio = 0.49 [95% CI 0.30-0.80], p=0.005) compared to IRS-L patients. In the rwPFS analysis, PD-1 vs. PD-L1 inhibitor was not a significant term in the adjusted

model (rwPFS adjusted hazard ratio 0.89 [95% CI 0.70-1.81], *p*=0.64), while in the OS analysis, PD-L1 inhibitors (vs. PD-1 inhibitors) were associated with significantly shorter OS (adjusted hazard ratio 1.93 [95% CI 1.14-3.25], *p*=0.014).

In the PD-L1 treated subset of the validation cohort (n=54 patients), by Kaplan Meier analysis, IRS-H patients had significantly longer rwPFS (IRS-H vs. IRS-L median rwPFS 28.2 [95% CI 15.1-NA] vs. 8.7 [95% CI 5.6-NA] months, adjusted hazard ratio = 0.26 [95% CI 0.09-0.72], *p*=0.009; **Supplementary Figure S11a**) and OS (IRS-H vs. IRS-L median OS 28.2 [95% CI 15.1-NA] vs. 10.6 [95% CI 8.8-NA] months, adjusted hazard ratio = 0.33 [95% CI 0.13-0.85], *p*=0.02; **Supplementary Figure S11b**) compared to IRS-L patients when adjusted for age, gender, most common tumor type (bladder cancer) vs. others, and line of therapy. In the PD-1 treated subset of the validation cohort (n=194 patients), by Kaplan Meier analysis, IRS-H patients had significantly longer TTNT by log-rank testing (IRS-H vs. IRS-L median TTNT 23.1 [95% CI 14.1-32.9] vs. 11.0 [95% CI 8.7-16.8] months, log-rank *p*=0.003), however when adjusted for age, gender, most common tumor type (melanoma) vs. others, and line of therapy, IRS status was not a significant predictor of TTNT (adjusted hazard ratio 0.62 [95% CI 0.38-1.01], *p*=0.054; **Supplementary Figure S11c**). Results were similar for OS analysis of the PD-1 subset, where IRS-H patients had significantly longer OS by log-rank testing (IRS-H vs. IRS-L median O Not reached [95% CI 32.9-NA] vs. 24.7 [95% CI 18.1-NA] months, log-rank *p*=0.047), however when adjusted IRS status was not a significant predictor of OS (adjusted hazard ratio 0.61 [95% CI 0.34-1.11], *p*=0.11 **Supplementary Figure S11d**). Taken together, despite the validation cohort including both PD-L1 and PD-1 therapies and having differing tumor type distributions compared to the discovery cohort, these results support the pan-solid tumor applicability of IRS to predict benefit of PD-(L)1 monotherapy.

ANALYSIS OF THE DISCOVERY AND VALIDATION COHORTS STRATIFIED BY IRS AND TMB STATUS

As shown in **Supplementary Figure S12a&b,** by Kaplan-Meier analysis of the pembrolizumab monotherapy cohort stratified by IRS and TMB status, while median rwPFS was significantly longer in IRS-H/TMB-H vs. IRS-H/TMB-L (median rwPFS Not reached [95% CI 16.6-NA] vs. 16.7 [95% CI 8.8-22.9] months, pairwise log-rank with Benjamini Hochberg adjusted p=0.02), median OS was not significantly different between IRS-H/TMB-H vs. IRS-H/TMB-L patients (median rwPFS Not reached [95% CI NA-NA] vs. 22.9 [95% CI 15.3-NA] months, pairwise log-rank with Benjamini Hochberg adjusted p=0.12). As shown in **Supplementary Figure S12c&d**, in the validation cohort, neither median PFS nor OS significantly differed between IRS-H/TMB-H vs. IRS-H/TMB-L patients (IRS-H/TMB-H vs. IRS-H/TMB-L median rwPFS 21.0 [95% CI 13.6-NA] vs. 28.2 [95% CI 17.1-NA] months, pairwise log-rank with Benjamini Hochberg adjusted p=0.31; median OS 40.4 [95% CI 30.4-NA] vs. Not Reached [95% CI 32.9-NA] months, pairwise log-rank with Benjamini Hochberg adjusted p=0.53). Of interest, in both the discovery and validation cohort (and overall SCMD population as described below), only a small minority of patients were IRS-L/TMB-H (2.1% and 4.0% of the discovery [monotherapy] and validation cohorts, respectively), and hence their benefit from PD-(L)1 monotherapy is unclear.

ASSESSMENT OF THE ABILITY OF *CDKN2A* DEEP DELETION STATUS TO ADD TO THE PREDICTIVE ABILITY OF IRS

Despite the fundamental limitations of all genomic biomarkers (including TMB) for predicting PD-(L)1 therapy response, multiple single genes have also been identified from translational research studies that may add to the ability of TMB and/or PD-L1 IHC to predict PD-(L)1 benefit[20-28]. Two recent reports have suggested that *CDKN2A* deep deletion (homozygous loss) status may improve upon TMB alone for predicting monotherapy PD-(L)1 benefit [20,28]. Hence, we assessed whether the inclusion of *CDKN2A* deep deletion status was an independent predictor of monotherapy PD-(L)1 benefit by adjusted Cox proportional hazards modeling in the subset of patients with valid *CDKN2A* deep deletion status (the StrataNGS limit of detection for deep deletions is 40% tumor content) from the discovery (n=310 [47.8%] of 648 after also excluding those treated with combination therapy) and validation (n=199 [79.9%] of

249) cohorts. As shown in **Supplementary Figure S13**, IRS status, but not *CDKN2A* deep deletion status, was a significant predictor of rwPFS and OS in both the discovery (rwPFS IRS-H vs. -L adjusted hazard ratio 0.48 [95% CI 0.33-0.69], $p<0.0001$; rwPFS *CDKN2A* deep deletion vs. *CDKN2A* wt adjusted hazard ratio 1.07 [95% CI 0.67-1.70], $p=0.78$; OS IRS-H vs. -L adjusted hazard ratio 0.48 [95% CI 0.32-0.74], $p=0.0009$; OS *CDKN2A* deep deletion vs. *CDKN2A* wt adjusted hazard ratio 1.02 [95% CI 0.61-1.72], $p=0.94$) and validation cohorts (rwPFS IRS-H vs. -L adjusted hazard ratio 0.47 [95% CI 0.30-0.75], $p=0.001$; rwPFS *CDKN2A* deep deletion vs. *CDKN2A* wt adjusted hazard ratio 1.58 [95% CI 0.97-2.57], $p=0.07$; OS IRS-H vs. -L adjusted hazard ratio 0.49 [95% CI 0.29-0.83], $p=0.008$; OS *CDKN2A* deep deletion vs. *CDKN2A* wt adjusted hazard ratio 0.99 [95% CI 0.56-1.75], $p=0.96$) cohorts when *CDKN2A* deep deletion status was added to the appropriate adjusted Cox proportional hazards model, confirming the limitations of genomic markers alone for predicting PD-(L)1 therapy response.

VALIDATION OF QTP AND IRS: STABILITY OF IRS ACROSS TEMPORAL SAMPLE COLLECTION VARIABILITY PRIOR TO CPI TREATMENT

Tissue based TMB has recently been shown to be stable for nearly all patients with advanced cancer through whole genome sequencing of sequential tissue samples,[29] however less is known about the stability of an integrative CGP + qTP model predicting pembrolizumab benefit. Hence, we assessed the impact of sample collection timing on IRS performance in the 310 patients in the discovery cohort that were also treated with a separate systemic line of therapy by stratifying patients by whether their samples were pre-systemic therapy and pembrolizumab vs. post-systemic therapy but pre-pembrolizumab. In the adjusted Cox proportional hazard model, IRS status (IRS-H [$n=113$] vs. IRS-L [$n=197$], adjusted hazard ratio 0.51 [95% CI 0.38-0.70], $p<0.0001$), but not pre-/post-systemic therapy collection timing (pre-systemic therapy [$n=229$] vs. post [$n=81$], adjusted hazard ratio 1.39 [95% CI 0.97-1.99], $p=0.07$), was

significantly associated with pembrolizumab rwPFS, demonstrating that sample collection timing does not significantly impact IRS performance.

Next, we directly assessed IRS stability across patients in the SCMD with sequentially tested tissue samples. As analyses presented thus far were limited to the most recently tested sample per patient (if testing had been performed more than once), we therefore identified 104 total patients in the SCMD who 1) had valid IRS scores from two specimens with different collection dates, 2) were confirmed to be of clonal origin as part of routine StrataNGS clinical testing, and 3) did not have PD(L)-1 or anti-CTLA4 therapy starting between the collection dates of the samples. As shown in **Supplementary Figure S14a**, the integrative IRS model scores were highly correlated (correlation coefficient=0.65) in paired specimens, and only 16 (15.3%) patients moved from the IRS-H to -L (n=6) or IRS-L to -H (n=6) (or vice versa), supporting the stability of the IRS across temporal sampling in the absence of immunotherapy.

Lastly, we assessed the performance of IRS in 181 patients (from 17 tumor types) who otherwise would have been included in the discovery or validation cohorts, but had their sample collected after starting PD-(L)1 therapy (any pembrolizumab containing line [n=92 patients] or other PD-(L)1 monotherapy [n=89 patients]). Hypothesizing that CGP testing in this clinical scenario would usually be performed as the patient was progressing on (or had already progressed on) PD-(L)1 therapy, we predicted that IRS would be minimally predictive of PD-(L)1 TTNT. In these 181 patients, IRS status was not predictive of PD-(L)1 TTNT by Kaplan Meier analysis (IRS-H [n=77] vs. IRS-L [n=104], median 14.8 [95% CI 10.8-16.8] vs. 10.7 [95% CI 8.6-14.7] months, adjusted hazard ratio IRS-H vs. IRS-L 0.88 [95% CI 0.62-1.27], p=0.50) when adjusted for age, gender, therapy line, most common tumor type (NSCLC) vs. others, PD-(L)1 type (pembrolizumab vs. other PD-[L]1), and monotherapy vs. combination therapy (for pembrolizumab); **Supplementary Figure S14b**). Together, these results support the stability and validity of IRS in tumor tissue samples collected prior to CPI treatment.

VALIDATION OF QTP AND IRS ROBUSTNESS OF IRS TO TUMOR CONTENT

For the integrative IRS model, the actual tumor content of a given sample is impacted both by normal cells unrelated to the gene expression component of the IRS model (such as benign epithelial cells), as well as tumor infiltrating lymphocytes (such as those that express PDCD1 and/or PD-L1) and cancer associated fibroblasts that express *ADAM12*[30-35], with these components (and the actual tumor content) directly relevant to the predictive ability of the IRS algorithm. Hence, an approach using "normal" RNA in a simple tumor content-based dilution series would be inappropriate to determine the clinical tumor content LOD for the IRS algorithm, even if not precluded by the difficulties in such an approach in multiplex RNA sequencing. Therefore to determine the tumor content limit of detection (LOD) for the IRS algorithm, we assessed the impact of tumor content on the predictive nature of the IRS algorithm. Thus, we included a continuous tumor content term (range: 20-100% [as the established LOD for accurate TMB estimation was determined as 20% tumor content]) and included this tumor content term in the overall adjusted CPH model for the IRS discovery cohort (including age, gender, most common tumor type [NSCLC] vs. others, therapy type [monotherapy/combination], and line of therapy). Importantly, while IRS remained a significant predictor of pembrolizumab TTNT (adjusted hazard ratio 0.49 [95% CI 0.39-0.63], p<0.0001), tumor content was not a significant predictor (adjusted hazard ratio 0.75 [95% CI 0.42-1.34], p=0.33; **Supplementary Figure S15a**). Kaplan Meier plots of pembrolizumab rwPFS stratified by IRS status is shown for relevant tumor content bins (20-35%, 40-70%, 75-100%) from analysis are shown in **Supplementary Figure S15b-d**. Similar results were observed in the validation cohort, as adding the same tumor content term to the overall adjusted CPH model in this cohort demonstrated that while IRS remained as a significant predictor of PD-(L)1 rwPFS (adjusted hazard ratio 0.55 [95% CI 0.36-0.84], p=0.006), tumor content was not a significant predictor (adjusted hazard ratio 2.5 [95% CI 0.94-6.56], p=0.07).

Lastly, we identified 64 subjects in the SCMD that otherwise would have been included in the discovery or validation cohorts, but the tumor content of the tested sample was <20%. As shown in **Supplementary Figure S15d**, the IRS model was not predictive of pembrolizumab TTNT (IRS-H [n=18] vs. IRS-L

[*n*=46], median TTNT 12.1 [95% CI 7.6-14.0] vs. 11.7 [95% CI 6.5-14.3] months; adjusted hazards ratio 0.73 [95% 0.32-1.70], p=0.47) when adjusted for age, gender, therapy line, most common tumor type (NSCLC) vs. others, PD-(L)1 type (pembrolizumab vs. other PD-[L]1), and monotherapy vs. combination therapy (for pembrolizumab). Taken together, these results further support the overall 20% tumor content LOD for the integrative IRS algorithm.

ADDITIONAL ANALYSES DEMONSTRATING THE PREDICTIVE NATURE OF IRS

As only 76 subjects in the validation cohort received PD-(L)1 blockade therapy in at least the second line, we instead leveraged the compendium of treatment data across SCMD patients not included in the discovery or validation cohorts to further confirm the predictive nature of the IRS biomarker. Across all 3,184 patients in the SCMD (*n*=592 IRS-H) with a non- PD-(L)1 or CTLA4 systemic therapy first line who otherwise met criteria for the discovery and validation cohorts, IRS status was not a significant predictor of non- PD-(L)1 or CTLA4 systemic therapy rwPFS by Cox proportional hazards modeling when adjusting for age, gender, most common tumor type (colorectal cancer) vs. others, and monotherapy vs. combination therapy (IRS-H vs. -L median rwPFS 7.0 [95% CI 6.1-7.9] vs. 8.5 [95% CI 8.0-9.0] months, adjusted hazard ratio 1.05 [95% CI 0.92-1.19], *p*=0.45), confirming the predictive nature of the IRS model (**Supplementary Figure S16c**). Next, given the mechanistic differences between CTLA4 and PD-(L)1 blockade, and the lack of additive or synergistic treatment effect between these agents in melanoma[36], we assessed the ability of IRS to stratify combined ipilimumab + nivolumab (CTLA4 + PD-1) benefit in a cohort of 70 patients (*n*=30 IRS-H) who otherwise would have been eligible for the validation cohort but received combined ipilimumab + nivolumab treatment (8 tumor types; 47% melanoma). As shown in **Supplementary Figure S16d**, after adjusting for age, gender, most common tumor type (melanoma) vs. others, and line of therapy, combined ipilimumab + nivolumab rwPFS (IRS-H vs. IRS-L median rwPFS 11.4 [95% CI 8.4-NA] vs. 10.8 [95% CI 5.9-NA] months, adjusted hazard ratio 0.78 [95% CI 0.34-1.76], p=0.55) did not significantly differ by IRS status.

SUPPLEMENTARY DISCUSSION

As the IRS was developed from a single integrative clinical platform using co-isolated DNA and RNA to generate TMB and highly quantitative gene expression assessment of the tumor and TME from 648 patients across 24 tumor types, the IRS model holds several potentially interesting biological insights. First, TMB, *PD-1* expression, and *PD-L1* expression were each independent predictors of pembrolizumab benefit, indicating a multiplicative predictive effect across these biomarkers representing increased antigenicity (TMB) and the direct targets of both PD-1 and PD-L1 monoclonal antibodies. While PD-L1 evaluation by IHC is the current FDA-approved biomarker to predict PD-(L)1 benefit either individually or in models[37-39], expression varies by antibody clone and nearly all studies show at least some responsive PD-L1–IHC low/negative patients[40-44]. In addition, PD-1 expression in both CD8+ and all lymphocytes has also been shown to be predictive of PD-(L)1 therapy benefit, most notably in Merkel cell carcinoma, where PD-1+ and PD-L1+ cell density, as well as close proximity of PD-1 and PD-L1+ cells, were associated with treatment response, while CD8+ cell density (nor CD8+ and PD-L1+ cell proximity) was not[39]. Our results are also consistent with the numerous translational research studies showing that while both high TMB and immune gene expression (or PD-L1 IHC) are predictive of PD-(L)1 benefit, these biomarkers are largely uncorrelated[19,42,45-56].

Increasing *TOP2A* and *ADAM12* expression were associated with decreased benefit from PD-(L)1 therapy in the IRS model. Although we have validated *TOP2A* as a marker of proliferating tumor cells **(Supplementary Figure S8)**, its significance in the IRS model is unclear. Although less is known about the direct role of *ADAM12* in CPI response, it is highly expressed by cancer associated fibroblasts CAFs—as shown through single cell sequencing studies and bulk tumor profiling—as a driver of feed forward TGF-β signaling, has been shown to act as a T cell co-stimulatory molecule expressed on some regulatory T cells, and has been identified in a signature of negative response to ICI in melanoma[30-35]. Of note, in colorectal cancer, where single cell sequencing demonstrated high *ADAM12* expression in CAFs[57], as well as urothelial carcinoma, TGF-β signaling from CAFs has been shown to drive T cell exclusion, a hallmark of low response to ICI[58-62]. Taken together, these results support additional

investigation into a potential mechanistic role for *ADAM12* in ICI resistance, as well as demonstrate the complementary nature of the integrative biomarkers in the IRS model, which integrates measurement of tumor neo-antigenicity (TMB), with quantification of key tumor and TME biomarkers.

SUPPLEMENTARY REFERENCES

1 Tomlins, S. A. *et al.* Development and Validation of StrataNGS, a Multiplex PCR, Semiconductor Sequencing-Based Comprehensive Genomic Profiling Test. *J Mol Diagn*, doi:10.1016/j.jmoldx.2021.08.005 (2021).

2 Priestley, P. *et al.* Pan-cancer whole-genome analyses of metastatic solid tumours. *Nature* **575**, 210-216, doi:10.1038/s41586-019-1689-y (2019).

3 Tomlins, S. A. *et al.* Real-World Performance of a Comprehensive Genomic Profiling Test Optimized for Small Tumor Samples. *JCO Precis Oncol* **5**, doi:10.1200/PO.20.00472 (2021).

4 McGranahan, N. *et al.* Clonal neoantigens elicit T cell immunoreactivity and sensitivity to immune checkpoint blockade. *Science* **351**, 1463-1469, doi:10.1126/science.aaf1490 (2016).

5 Anagnostou, V. *et al.* Multimodal genomic features predict outcome of immune checkpoint blockade in non-small-cell lung cancer. *Nature Cancer* **1**, 99-111, doi:10.1038/s43018-019-0008-8 (2020).

6 Conroy, J. M. *et al.* Analytical Validation of a Next-Generation Sequencing Assay to Monitor Immune Responses in Solid Tumors. *J Mol Diagn* **20**, 95-109, doi:10.1016/j.jmoldx.2017.10.001 (2018).

7 Wang, Q. *et al.* Unifying cancer and normal RNA sequencing data from different sources. *Sci Data* **5**, 180061, doi:10.1038/sdata.2018.61 (2018).

8 Niknafs, Y. S. *et al.* MiPanda: A Resource for Analyzing and Visualizing Next-Generation Sequencing Transcriptomics Data. *Neoplasia* **20**, 1144-1149, doi:10.1016/j.neo.2018.09.001 (2018).

9 Harms, K. L. *et al.* Virus-positive Merkel Cell Carcinoma Is an Independent Prognostic Group with Distinct Predictive Biomarkers. *Clin Cancer Res* **27**, 2494-2504, doi:10.1158/1078-0432.CCR-20-0864 (2021).

10 Cerami, E. *et al.* The cBio cancer genomics portal: an open platform for exploring multidimensional cancer genomics data. *Cancer Discov* **2**, 401-404, doi:10.1158/2159-8290.CD-12-0095 (2012).

11 Hovelson, D. H. *et al.* Targeted DNA and RNA Sequencing of Paired Urothelial and Squamous Bladder Cancers Reveals Discordant Genomic and Transcriptomic Events and Unique Therapeutic Implications. *Eur Urol* **74**, 741-753, doi:10.1016/j.eururo.2018.06.047 (2018).

12 Plaska, S. W. *et al.* Targeted RNAseq of Formalin-Fixed Paraffin-Embedded Tissue to Differentiate Among Benign and Malignant Adrenal Cortical Tumors. *Horm Metab Res* **52**, 607-613, doi:10.1055/a-1212-8803 (2020).

13 Salami, S. S. *et al.* Transcriptomic heterogeneity in multifocal prostate cancer. *JCI Insight* **3**, doi:10.1172/jci.insight.123468 (2018).

14 Tomlins, S. A. *et al.* Integrative molecular concept modeling of prostate cancer progression. *Nat Genet* **39**, 41-51, doi:10.1038/ng1935 (2007).

15 Tomlins, S. A. *et al.* Development and Validation of StrataNGS, a Multiplex PCR, Semiconductor Sequencing-Based Comprehensive Genomic Profiling Test. *J Mol Diagn* **23**, 1515-1533, doi:10.1016/j.jmoldx.2021.08.005 (2021).

16 Zehir, A. *et al.* Mutational landscape of metastatic cancer revealed from prospective clinical sequencing of 10,000 patients. *Nat Med* **23**, 703-713, doi:10.1038/nm.4333 (2017).

17 Cronin, M. *et al.* Analytical validation of the Oncotype DX genomic diagnostic test for recurrence prognosis and therapeutic response prediction in node-negative, estrogen receptor-positive breast cancer. *Clin Chem* **53**, 1084-1091, doi:10.1373/clinchem.2006.076497 (2007).

18 Simon, R. Roadmap for developing and validating therapeutically relevant genomic classifiers. *J Clin Oncol* **23**, 7332-7341, doi:10.1200/JCO.2005.02.8712 (2005).

19 Litchfield, K. *et al.* Meta-analysis of tumor- and T cell-intrinsic mechanisms of sensitization to checkpoint inhibition. *Cell* **184**, 596-614 e514, doi:10.1016/j.cell.2021.01.002 (2021).

20 Han, G. *et al.* 9p21 loss confers a cold tumor immune microenvironment and primary resistance to immune checkpoint therapy. *Nat Commun* **12**, 5606, doi:10.1038/s41467-021-25894-9 (2021).

21 Sholl, L. M. Biomarkers of response to checkpoint inhibitors beyond PD-L1 in lung cancer. *Mod Pathol*, doi:10.1038/s41379-021-00932-5 (2021).

22 Di Federico, A., De Giglio, A., Parisi, C. & Gelsomino, F. STK11/LKB1 and KEAP1 mutations in non-small cell lung cancer: Prognostic rather than predictive? *Eur J Cancer* **157**, 108-113, doi:10.1016/j.ejca.2021.08.011 (2021).

23 Skoulidis, F. *et al.* STK11/LKB1 Mutations and PD-1 Inhibitor Resistance in KRAS-Mutant Lung Adenocarcinoma. *Cancer Discov* **8**, 822-835, doi:10.1158/2159-8290.CD-18-0099 (2018).

24 Shen, J. *et al.* ARID1A deficiency promotes mutability and potentiates therapeutic antitumor immunity unleashed by immune checkpoint blockade. *Nat Med* **24**, 556-562, doi:10.1038/s41591-018-0012-z (2018).

25 Miao, D. *et al.* Genomic correlates of response to immune checkpoint therapies in clear cell renal cell carcinoma. *Science* **359**, 801-806, doi:10.1126/science.aan5951 (2018).

26 Braun, D. A. *et al.* Clinical Validation of PBRM1 Alterations as a Marker of Immune Checkpoint Inhibitor Response in Renal Cell Carcinoma. *JAMA Oncol* **5**, 1631-1633, doi:10.1001/jamaoncol.2019.3158 (2019).

27 Liu, X. D. *et al.* PBRM1 loss defines a nonimmunogenic tumor phenotype associated with checkpoint inhibitor resistance in renal carcinoma. *Nat Commun* **11**, 2135, doi:10.1038/s41467-020-15959-6 (2020).

28 Ebot, E. M. *et al.* Deletions on 9p21 are associated with worse outcomes after anti-PD-1/PD-L1 monotherapy but not chemoimmunotherapy. *NPJ Precis Oncol* **6**, 44, doi:10.1038/s41698-022-00286-4 (2022).

29 van de Haar, J. *et al.* Limited evolution of the actionable metastatic cancer genome under therapeutic pressure. *Nat Med* **27**, 1553-1563, doi:10.1038/s41591-021-01448-w (2021).

30 Yu, C. *et al.* A five-gene signature is a prognostic biomarker in pan-cancer and related with immunologically associated extracellular matrix. *Cancer Med* **10**, 4629-4643, doi:10.1002/cam4.3986 (2021).

31 Liu, Y. *et al.* ADAM12 is a costimulatory molecule that determines Th1 cell fate and mediates tissue inflammation. *Cell Mol Immunol* **18**, 1904-1919, doi:10.1038/s41423-020-0486-8 (2021).

32 Cheon, D. J. *et al.* ADAM12 is a prognostic factor associated with an aggressive molecular subtype of high-grade serous ovarian carcinoma. *Carcinogenesis* **36**, 739-747, doi:10.1093/carcin/bgv059 (2015).

33 Cui, C. *et al.* Ratio of the interferon-gamma signature to the immunosuppression signature predicts anti-PD-1 therapy response in melanoma. *NPJ Genom Med* **6**, 7, doi:10.1038/s41525-021-00169-w (2021).

34 Ruff, M. *et al.* The Disintegrin and Metalloprotease ADAM12 Is Associated with TGF-beta-Induced Epithelial to Mesenchymal Transition. *PLoS One* **10**, e0139179, doi:10.1371/journal.pone.0139179 (2015).

35 Atfi, A. *et al.* The disintegrin and metalloproteinase ADAM12 contributes to TGF-beta signaling through interaction with the type II receptor. *J Cell Biol* **178**, 201-208, doi:10.1083/jcb.200612046 (2007).

36 Palmer, A. C., Izar, B., Hwangbo, H. & Sorger, P. K. Predictable Clinical Benefits without Evidence of Synergy in Trials of Combination Therapies with Immune-Checkpoint Inhibitors. *Clin Cancer Res* **28**, 368-377, doi:10.1158/1078-0432.CCR-21-2275 (2022).

37 Medicine, F. (2021).

38 Yearley, J. H. *et al.* PD-L2 Expression in Human Tumors: Relevance to Anti-PD-1 Therapy in Cancer. *Clin Cancer Res* **23**, 3158-3167, doi:10.1158/1078-0432.CCR-16-1761 (2017).

39 Giraldo, N. A. *et al.* Multidimensional, quantitative assessment of PD-1/PD-L1 expression in patients with Merkel cell carcinoma and association with response to pembrolizumab. *J Immunother Cancer* **6**, 99, doi:10.1186/s40425-018-0404-0 (2018).

40 Garon, E. B. *et al.* Pembrolizumab for the treatment of non-small-cell lung cancer. *N Engl J Med* **372**, 2018-2028, doi:10.1056/NEJMoa1501824 (2015).

41 Robert, C. *et al.* Pembrolizumab versus Ipilimumab in Advanced Melanoma. *N Engl J Med* **372**, 2521-2532, doi:10.1056/NEJMoa1503093 (2015).

42 Herbst, R. S. *et al.* Predictive correlates of response to the anti-PD-L1 antibody MPDL3280A in cancer patients. *Nature* **515**, 563-567, doi:10.1038/nature14011 (2014).

43 Tumeh, P. C. *et al.* PD-1 blockade induces responses by inhibiting adaptive immune resistance. *Nature* **515**, 568-571, doi:10.1038/nature13954 (2014).

44 Mahoney, K. M. & Atkins, M. B. Prognostic and predictive markers for the new immunotherapies. *Oncology (Williston Park)* **28 Suppl 3**, 39-48 (2014).

45 Cristescu, R. *et al.* Pan-tumor genomic biomarkers for PD-1 checkpoint blockade-based immunotherapy. *Science* **362**, doi:10.1126/science.aar3593 (2018).

46 Lee, J. S. & Ruppin, E. Multiomics Prediction of Response Rates to Therapies to Inhibit Programmed Cell Death 1 and Programmed Cell Death 1 Ligand 1. *JAMA Oncol* **5**, 1614-1618, doi:10.1001/jamaoncol.2019.2311 (2019).

47 Rolfo, C. *et al.* Liquid Biopsy for Advanced NSCLC: A Consensus Statement From the International Association for the Study of Lung Cancer. *J Thorac Oncol* **16**, 1647-1662, doi:10.1016/j.jtho.2021.06.017 (2021).

48 Taube, J. M. *et al.* Colocalization of inflammatory response with B7-h1 expression in human melanocytic lesions supports an adaptive resistance mechanism of immune escape. *Sci Transl Med* **4**, 127ra137, doi:10.1126/scitranslmed.3003689 (2012).

49 Chen, D. S. & Mellman, I. Elements of cancer immunity and the cancer-immune set point. *Nature* **541**, 321-330, doi:10.1038/nature21349 (2017).

50 Sanmamed, M. F. & Chen, L. A Paradigm Shift in Cancer Immunotherapy: From Enhancement to Normalization. *Cell* **175**, 313-326, doi:10.1016/j.cell.2018.09.035 (2018).

51 Ayers, M. *et al.* IFN-gamma-related mRNA profile predicts clinical response to PD-1 blockade. *J Clin Invest* **127**, 2930-2940, doi:10.1172/JCI91190 (2017).

52 Fountzilas, E., Kurzrock, R., Hiep Vo, H. & Tsimberidou, A. M. Wedding of Molecular Alterations and Immune Checkpoint Blockade: Genomics as a Matchmaker. *J Natl Cancer Inst*, doi:10.1093/jnci/djab067 (2021).

53 Ott, P. A. *et al.* T-Cell-Inflamed Gene-Expression Profile, Programmed Death Ligand 1 Expression, and Tumor Mutational Burden Predict Efficacy in Patients Treated With Pembrolizumab Across 20 Cancers: KEYNOTE-028. *J Clin Oncol* **37**, 318-327, doi:10.1200/JCO.2018.78.2276 (2019).

54 Lu, S. *et al.* Comparison of Biomarker Modalities for Predicting Response to PD-1/PD-L1 Checkpoint Blockade: A Systematic Review and Meta-analysis. *JAMA Oncol* **5**, 1195-1204, doi:10.1001/jamaoncol.2019.1549 (2019).

55 Zhang, Z. *et al.* RNF2 ablation reprograms the tumor-immune microenvironment and stimulates durable NK and CD4+ T-cell-dependent antitumor immunity. *Nature Cancer* **2**, 1018-1038, doi:10.1038/s43018-021-00263-z (2021).

56 Cristescu, R. *et al.* Transcriptomic Determinants of Response to Pembrolizumab Monotherapy Across Solid Tumor Types. *Clin Cancer Res*, doi:10.1158/1078-0432.CCR-21-3329 (2021).

57 Pelka, K. *et al.* Spatially organized multicellular immune hubs in human colorectal cancer. *Cell* **184**, 4734-4752 e4720, doi:10.1016/j.cell.2021.08.003 (2021).

58 Mariathasan, S. *et al.* TGFbeta attenuates tumour response to PD-L1 blockade by contributing to exclusion of T cells. *Nature* **554**, 544-548, doi:10.1038/nature25501 (2018).

59 Tauriello, D. V. F. *et al.* TGFbeta drives immune evasion in genetically reconstituted colon cancer metastasis. *Nature* **554**, 538-543, doi:10.1038/nature25492 (2018).

60 Jiang, P. *et al.* Signatures of T cell dysfunction and exclusion predict cancer immunotherapy response. *Nat Med* **24**, 1550-1558, doi:10.1038/s41591-018-0136-1 (2018).

61 Bonaventura, P. *et al.* Cold Tumors: A Therapeutic Challenge for Immunotherapy. *Front Immunol* **10**, 168, doi:10.3389/fimmu.2019.00168 (2019).

62 Gajewski, T. F., Schreiber, H. & Fu, Y. X. Innate and adaptive immune cells in the tumor microenvironment. *Nat Immunol* **14**, 1014-1022, doi:10.1038/ni.2703 (2013).

63 Rousseau, B. *et al.* The Spectrum of Benefit from Checkpoint Blockade in Hypermutated Tumors. *N Engl J Med* **384**, 1168-1170, doi:10.1056/NEJMc2031965 (2021).

SUPPLEMENTARY FIGURE LEGENDS

Figure S1. Overall study diagram from the Strata Clinical Molecular Database (SCMD) used to develop and validate the Immunotherapy Response Score (IRS).

Disposition of patients from the Strata Trial (NCT03061305) used to develop and validate IRS are shown. Included populations are indicated by gray boxes. As patients could contribute to multiple analyses (e.g., a subject treated with first line angiogenesis inhibitor and second line pembrolizumab could be eligible for both the "Non-IO 1st line analysis" and the "Discovery cohort" as long as they met both inclusion/exclusion criteria [including the sample was collected before both lines of therapy]), the number of shared patients is indicated by green arrows at the highest branch point. The overall SCMD population is shown in bolded yellow. No patients were shared between the discovery and validation cohorts (bolded blue). Analyses on groups are indicated by Figure numbers. *For the "Clonal samples with IRS" group, shared subjects are not shown due to diagrammatic complexity (greatest shared subjects n=26 with "Non-IO 1st line").

Figure S2. Assignment of therapy lines from real world treatment data

For all Strata Trial (NCT03061305) subjects with treatment data (treatment start and stop dates), standardized assignment of adjuvant/systemic therapy lines was performed accounting for adjuvant/systemic therapy, monotherapy/combination therapy, potential overlap of treatment start/stop dates and repeating lines of therapy (whether monotherapy in combination). Assigned treatment lines and an example of real world-progression free survival measurement by time to next therapy (TTNT; start date of therapy to start date of subsequent therapy) are shown for a patient with metastatic renal cell carcinoma.

Figure S3. Time to next therapy (TTNT) of patients in the Strata Clinical Molecular Database (SCMD) by line of therapy.

Real-world progression-free survival by TTNT per first (Line 1, blue line), second (Line 2, green line) or third or more (Line 3+, orange line) line of therapy for the 9,899 patients in the SCMD having treatment

data from at least one systemic antineoplastic agent. The number (n) of patients, events, and median rwPFS (with 95% confidence intervals [CI]) for each group are shown, along with the overall log rank p value.

Figure S4. Strata Clinical Molecular Database (SCMD) non-small cell lung cancer (NSCLC) analysis.

a) Real world progression free survival (rwPFS, by time to next therapy) in SCMD of patients with first line NSCLC when treated with first generation (gen) targeted *EGFR*, *ALK*, *ROS1* or *MET* tyrosine kinase inhibitors ([TKI]; erlotinib, gefitinib, or crizotinib, *n*=37) versus later-generation inhibitors (*n*=120, green) is shown by Kaplan-Meier analysis with the adjusted hazard ratio (HR) and p-value shown for later vs. first generation inhibitor. The number (n) of patients, events, and median rwPFS (with 95% confidence intervals [CI]) for each group are shown. **b)** rwPFS in SCMD patients with first line NSCLC when treated with a first line oncogene NCCN preferred targeted monotherapy TKI based on whether the treatment occurred before (*n*=57, blue line; treatment decision made from orthogonal testing) or after (*n*=72, green line; treatment decision made using StrataNGS) receiving StrataNGS CGP test results is shown by Kaplan-Meier analysis with the adjusted hazard ratio (HR) and p-value shown for treatment before vs. after receiving CGP results. **c)** rwPFS in SCMD of patients with first line NSCLC treated with a biomarker matched, first line oncogene NCCN preferred targeted monotherapy TKI after receiving StrataNGS results based on whether the sample 1) passed both StrataNGS sample input criteria and relevant sequencing QC metrics (*n*=48) or 2) did not meet sample input or failed sequencing QC metrics but reported a therapy matched biomarker (*n*=17) is shown by Kaplan-Meier analysis with the adjusted hazard ratio (HR) and p-value shown for failed (2) vs. passed (1) QC metrics.

Figure S5. Correlation of real-world pembrolizumab progression-free survival (rwPFS) and overall survival (OS).

Correlation for pembrolizumab rwPFS by time to next therapy (TTNT) and OS for patients in the discovery cohort with more than one line of systemic therapy. Colored boxes indicate patients discussed in the **Supplementary Results**

Figure S6. PD-(L)1 monotherapy real world progression free survival (rwPFS) and overall survival (OS) by tumor mutation burden (TMB) status.

a) Pembrolizumab monotherapy (PD-1) rwPFS (by time to next therapy) in the discovery cohort stratified by TMB groups (TMB-High [H] ≥10 mutations/megabase by StrataNGS testing vs. TMB-Low [L]) is shown by Kaplan Meier analysis with the adjusted hazard ratio (HR) and p value for TMB-H vs. -L groups. The number (n) of patients, events, and median rwPFS (with 95% confidence intervals [CI]) for each group are shown. b) As in a except OS. c-d) As in a-b, except assessing the independent validation cohort of patients treated with non-pembrolizumab PD-(L)1 monotherapy.

Figure S7. Housekeeping gene selection and validation, accuracy vs qRT-PCR, and replicate amplicon correlation for the quantitative expression component of the integrated comprehensive genomic profiling and quantitative transcriptional profiling (CGP + qTP) laboratory developed test used to report the Immunotherapy Response Score (IRS).

IRS is reported from an integrated CGP + qTP test that combines comprehensive genomic profiling (CGP) from the analytically and clinically validated StrataNGS test with in-parallel quantitative transcriptional profiling (qTP) by multiplex RT-PCR based next generation sequencing. **a**) Initial pre-clinical versions of the qTP panel contained 6 “positive control” genes across two RNA primer pools previously used in the RNA fusion component of the Oncomine Focus/Precision Assay (OPA positive).

To evaluate the suitability of these markers as pan-cancer housekeeping genes for quantitative expression profiling, we performed a multi-part evaluation of transcriptome profiles of pan-cancer, pan-normal tissue stability. Average expression levels (in transcripts per million [TPM]), and coefficient of variation (CV) are shown from >20,000 tumor, normal and cancer cell line samples for OPA positive genes, additional candidate housekeeping genes from an assessment of TCGA (TCGA stable) and the commonly used housekeeping gene GAPDH. The eight bolded genes were included in gene expression panels used to develop the CGP + qTP test and IRS. **b**) Letter-value (boxen) plots of normalized expression for the three final housekeeping genes used in the qTP panel (*CIAO1*, *EIF2B1* and *HMBS*) and the remaining five candidates are shown from a consecutive 4-month period of CGP + qTP clinical testing of samples with reportable quantitative expression using the current test version (n=3,417 samples; regardless of tumor content). Values beyond boxes with overlapping 95% CI are not shown. **c**) The clinical accuracy of the qTP component was first determined by determining target gene expression concordance with hydrolysis probe based qRT-PCR through representational validation on 24 FFPE tumor samples. Expression of included individual target gene amplicons (n=32) are shown by color. The concordance correlation coefficient for the panel-wide validation as well as only the four IRS expression biomarkers (*PD-L1*, *PD-1*, *ADAM12* and *TOP2A*) are shown. The line of equality is shown. **d-f**) Two separate *PD-L1*, *PD-1* and *ADAM12* amplicons are present in the current qTP panel (only one of two *ADAM12* amplicons was also present on all previous panels used to develop and validate IRS). As multiplex PCR based qTP enables unambiguous read assignment to each target gene amplicon, we determined the correlation coefficients of the replicate amplicons across the 24,463 Strata Trial samples used to assess IRS distribution (n=7,911 samples on panels with both *ADAM12* amplicons). Scatter plots are shown overlying a density heatmap. The line of equality is shown.

Figure S8. Accuracy vs. clinical immunohistochemistry and reproducibility for the quantitative expression component of the integrated comprehensive genomic profiling and quantitative transcriptional profiling (CGP + qTP) laboratory developed test used to report the Immunotherapy Response Score (IRS).

IRS is reported from an integrated CGP + qTP test that combines comprehensive genomic profiling (CGP) from the analytically and clinically validated StrataNGS test with in-parallel quantitative transcriptional profiling (qTP) by multiplex RT-PCR based next generation sequencing. **a**) Accuracy of the *PD-L1* qTP component of IRS was validated against clinical IHC using a cohort of 276 non-small cell lung cancer (NSCLC) formalin fixed paraffin embedded (FFPE) tumor samples with reportable qTP (including tumor content [TC] ≥ 20%) and PD-L1 IHC expression by the 22C3 clone (using tumor proportion score [TPS]) in accompanying pathology reports. Box plots of qTP *PD-L1* expression stratified by TPS bin (0% [n=84], 1-49% [n=111], and ≥50% [n=81]) are shown (upper error bars indicate the largest value within 1.5 * interquartile range (IQR) above the 75th %; lower error bars indicate the smallest value within 1.5 * IQR below the 25th %), along with results from the Kruskal Wallis [K.W.] test (with Jonckheere-Terpstra [J.T.] trend test [increasing median from 0%, 1-49%, and ≥50%]). Only 24 of these samples came from the 154 patients in the propensity matched first line NSCLC treatment analysis (see **Figure 4**), precluding direct assessment of IRS vs. PD-L1 IHC for predicting pembrolizumab benefit. However, IRS status could be generated for all 276 NSCLC samples with PD-L1 IHC and the percentage of IRS-H samples by TPS bin is shown in dark blue. **b**) Accuracy of the *TOP2A* qTP component of IRS was validated against clinical IHC using a cohort of 956 FFPE tumor tissue samples (36 tumor types) with reportable qTP (including TC ≥ 20%) with proliferation index (percentage of Ki67 positive tumor cells) in accompanying pathology reports. The Pearson correlation coefficient of qTP *TOP2A* expression vs. clinical proliferation index from the scatter plot is shown with 95% confidence interval [CI] and p-value, with points overlying a density heatmap and the line of best fit indicated by the dashed line. **c**) Panel wide qTP reproducibility between operators, lots, and instrumentation was established using separate replicate nucleic acid aliquots isolated from FFPE tumor samples. Twenty-seven unique samples were assessed by two operators on different days using different library preparation instrumentation,

different library preparation reagent lots, and different templating and sequencing lots and instruments. For each sample, the maximum and minimum nRPM for each target gene across all replicates was plotted (individual target gene amplicons are shown by color) and the concordance correlation coefficient was determined. **d**) As in **c**), except reproducibility of IRS was determined by plotting the maximum and minimum IRS across all replicates for each sample and the concordance correlation coefficient was determined. Qualitative agreement of IRS status (High vs. Low) from the maximum and minimum IRS score across all replicates was also determined.

Figure S9. Lasso-penalized Cox proportional hazards regression for Immunotherapy Response Score (IRS) development.

To develop an integrative PD-1/PD-L1 blockade benefit predictive model, we performed Lasso-penalized Cox proportional hazards regression with five-fold cross-validation in the 648 patient pembrolizumab (PD-1 therapy) discovery cohort to perform feature selection from tumor mutation burden (TMB; log_2) and 23 candidate immune and proliferation expression biomarkers associated with pembrolizumab TTNT. The Lasso penalty term was chosen as the value which maximized the concordance index (top panel; gray line) via 5-fold cross validation, with the coefficients shown for TMB and the 23 candidate expression biomarkers vs. alpha (α) (bottom panel), resulting in a five-term model including TMB, *PD-1*, *PD-L1*, *ADAM12*, and *TOP2A*.

Figure S10. Pembrolizumab overall survival (OS) by Immunotherapy Response Score (IRS) status.

a) Pembrolizumab OS in the discovery cohort stratified by IRS groups is shown by Kaplan Meier analysis with the adjusted hazard ratio (HR) and p value (adjusted by variables shown in **b**) for IRS-H vs. -L. The number (n) of patients, events, and median rwPFS (with 95% confidence intervals [CI]) for each group are shown. **b)** Forest plot of variables included in the adjusted Cox proportional hazards model used to

evaluate the ability of IRS to stratify pembrolizumab OS. Adjusted hazard ratios with 95% confidence intervals (CI) are shown for each variable. Statistically significant variables are bolded.

Figure S11. Real world progression free survival (rwPFS) and overall survival (OS) by Immunotherapy Response Score (IRS) status in the validation cohort stratified by PD-1 vs. PD-L1 therapy.

a) rwPFS (by time to next therapy) for the monotherapy PD-L1 treated subset of the validation cohort stratified by IRS groups is shown by Kaplan Meier analysis with the adjusted hazard ratio (HR) and p value for IRS-H vs. -L groups. The number (n) of patients, events, and median rwPFS (with 95% confidence intervals [CI]) for each group are shown. **b**) As in **a)**, except assessing OS. **c&d)** As in **a&b)**, except assessing TTNT **c**) and OS **d**) for the monotherapy PD-1 treated subset of the validation cohort. In addition to the adjusted HRs and p-value, the log-rank p-value is also shown.

Figure S12. PD-(L)1 monotherapy real world progression free survival (rwPFS) and overall survival (OS) by Immunotherapy Response Score (IRS) status and Tumor Mutation Burden (TMB).

a) Pembrolizumab monotherapy rwPFS in the discovery cohort stratified by IRS (IRS-High [-H] vs. -Low [L]) and TMB (TMB-H [≥10 mutations/megabase] vs. TMB-L is shown by Kaplan Meier analysis. Benjamini Hochberg (BH) adjusted p-value for pairwise log-rank test between the IRS-H/TMB-H and IRS-H/TMB-L groups is shown. The number (n) of patients, events, and median rwPFS (with 95% confidence intervals [CI]) for the analzyed groups are shown. **b**) as in **a**), expect OS. **c&d)** As in **a&b**, except assessing rwPFS (**c**) and OS (**d**) in the independent validation cohort of patients treated with non-pembrolizumab PD-(L)1 monotherapy.

Figure S13. *CDKN2A* deep deletion status does not add to Immunotherapy Response Score (IRS) for predicting PD-(L)1 monotherapy real world progression free survival (rwPFS) or overall survival (OS).

a) Pembrolizumab monotherapy rwPFS in the subset (*n*=310) of the discovery cohort evaluable for *CDKN2A* deep deletion (equivalent to homozygous/two copy deletion if diploid) status (≥ 40% tumor content and evaluable copy number alterations) stratified by IRS groups is shown by Kaplan Meier analysis with the adjusted hazard ratio (HR) and p value for IRS-High vs. -L groups. The number (n) of patients, events, and median rwPFS (with 95% confidence intervals [CI]) for each group are shown. The forest plot shows adjusted hazard ratios with 95% confidence intervals (CI) for IRS (IRS-H vs. IRS-L) and *CDKN2A* deep deletion status (*CDKN2A* deep deletion present vs. *CDKN2A* deep deletion not present) in the same adjusted model. **b**) As in **a**, except assessing OS. **c&d)** As in **a&b**, except assessing TTNT (**c**) and OS (**d**) in the subset (*n*=199) of the independent validation cohort evaluable for *CDKN2A* deep deletion treated with non-pembrolizumab PD-(L)1 monotherapy.

Figure S14. Immunotherapy Response Score (IRS) is robust to pre-PD-(L)1 sample collection timing.

a) Pearson correlation of IRS from clonal tumor specimens from the same patient with different collection dates and no checkpoint-inhibitor therapy in between tested sample collection dates (n=104 patients). **b)** PD-(L)1 rwPFS stratified by IRS group in 181 patients who otherwise would have been included in the discovery or validation cohorts but had their samples collected after starting PD-(L)1 therapy is shown by Kaplan Meier analysis with the adjusted hazard ratio (HR) and p value for IRS-H vs. IRS-L. The number (n) of patients, events, and median rwPFS (with 95% confidence intervals [CI]) for each group are shown.

Figure S15. Immunotherapy Response Score (IRS) is robust to variable tumor content.

a) A continuous tumor content term was included in the adjusted Cox proportional hazards (CPH) model for pembrolizumab real world progression free survival (rwPFS; by time to next therapy) in the overall discovery cohort as in **Figure 1b** (including age, gender, most common tumor type [NSCLC] vs. others,

therapy type [monotherapy/combination], and line of therapy). Adjusted hazard ratios with 95% confidence intervals (CIs) are shown for each variable with statistically significant variables bolded. **b-d)** Pembrolizumab rwPFS binned by tumor content (20-35%, 40-70%, and >70%) and stratified by IRS groups is shown by Kaplan Meier analysis. The number (n) of patients, events, and median rwPFS (with 95% confidence intervals [CI]) for each group are shown. **e**) PD-(L)1 rwPFS stratified by IRS group in 64 patients who otherwise would have been included in the discovery or validation cohorts except the tested sample tumor content was <20% is shown by Kaplan Meier analysis with the adjusted hazard ratio (HR) and p value for IRS-H vs. -L groups. The number (n) of patients, events, and median rwPFS (with 95% confidence intervals [CI]) for each group are shown

Figure S16. Additional analyses supporting the predictive nature of the Immunotherapy Response Score (IRS) biomarker.

a&b) To establish the predictive nature of the IRS model, we assessed an internal comparator cohort for the pembrolizumab monotherapy cohort, consisting of the 146 patients who had received a previous line of systemic therapy prior to monotherapy pembrolizumab therapy. For each patient, real-world progression free survival (rwPFS) was determined for the line of systemic therapy immediately prior to pembrolizumab and the pembrolizumab monotherapy line, with rwPFS stratified by IRS status (**see Figure 3**). Here, Kaplan Meier analysis of pembrolizumab monotherapy rwPFS (purple) vs. prior systemic therapy rwPFS (yellow) in the subset of non-microsatellite instability high (MSI-H) tumors in non-PD-(L)1 monotherapy approved tumor types is shown, along with the log-rank p-value between pembrolizumab and the prior therapy in the **a**) IRS-high [H] and **b**) IRS-low [L] populations. The number (n) of patients, events, and median rwPFS (with 95% confidence intervals [CI]) for each group are shown. **c**) To confirm the predictive nature of the IRS model we determined rwPFS in 3,184 patients in the SCMD treated with systemic first-line non-immunotherapy (IO), who otherwise met criteria for the discovery and validation cohorts. Kaplan-Meier analysis of non-IO systemic first line rwPFS stratified by

IRS status is shown with the adjusted hazard ratio (HR) and p-value for IRS-H vs. IRS-L groups. **d**) Ipilimumab + nivolumab (ipi+nivo) rwPFS in 70 patients who otherwise would have been eligible for the validation cohort but received combination ipilimumab (CTLA4) + nivolumab (PD-1) therapy stratified by IRS status is shown by Kaplan Meier analysis with the adjusted hazard ratio (HR) and p value for IRS-H vs. -L groups.

Figure S17. Clinical utility of integrated comprehensive genomic profiling and quantitative transcriptional profiling (CGP + qTP) outside of immunotherapy treatment decision making.

IRS is reported from an integrated CGP + qTP test that combines comprehensive genomic profiling (CGP) from the analytically and clinically validated StrataNGS test with in-parallel quantitative transcriptional profiling (qTP) by multiplex RT-PCR based next generation sequencing. **a**) Accuracy of *ESR1* (*estrogen receptor*; *ER*) by qTP validated against clinical IHC using a cohort of 300 breast cancer formalin fixed paraffin embedded (FFPE) tumor samples with reportable qTP (including tumor content [TC] ≥ 20%) and ER IHC expression (by % tumor cells positive) in accompanying pathology reports. The entire cohort was used for accuracy, however the cohort was randomly split into equivalent training (n=150) and validation (n=150) cohorts to establish clinical validity (see **d**) prior to performing the accuracy assessment. The correlation coefficient of qTP *ER* expression vs. clinical ER % tumor cells positive (log_2) from the scatter plot is shown with 95% confidence interval [CI] and p-value, with points overlying a density heatmap and the line of best fit indicated by the dashed line. **b**) Accuracy of *PGR* (*progesterone receptor*; *PR*) by qTP validated against clinical IHC using a cohort of 291 breast cancer formalin fixed paraffin embedded (FFPE) tumor samples with reportable qTP (including tumor content [TC] ≥ 20%) and PR IHC expression (by % tumor cells positive) in accompanying pathology reports. The entire cohort was used for accuracy, however the cohort was randomly split into equivalent training (n=145) and validation (n=146) cohorts to establish clinical validity (see **e**) prior to performing the accuracy assessment. The correlation coefficient of qTP *PR* expression vs. clinical PR % tumor cells

positive (log_2) from the scatter plot is shown with 95% confidence interval [CI] and p-value, with points overlying a density heatmap and the line of best fit indicated by the dashed line. **c**) Accuracy of the *HER2* (*ERBB2*) by qTP was validated against clinical IHC using a cohort of 545 breast cancer formalin fixed paraffin embedded (FFPE) tumor samples with reportable qTP (including tumor content [TC] ≥ 20%) and HER2 IHC expression (0 [n=233], 1+ [n=172], 2+ [n=100] or 3+ [n=40]) in accompanying pathology reports. The entire cohort was used for accuracy, however the cohort was randomly split into equivalent training (n=273) and validation (n=272) cohorts to establish clinical validity (see **f**) prior to performing the accuracy assessment. Box plots of qTP *HER2* expression (upper error bars indicate the largest value within 1.5 * interquartile range (IQR) above the 75th %; lower error bars indicate the smallest value within 1.5 * IQR below the 25th %) stratified by clinical IHC category are shown in the accuracy cohort, along with the Kruskal Wallis (K.W.) test p-value and Jonckheere-Terpstra [J.T.] trend test p-value (increasing median from 0 to 1+ to 2+ to 3+). **d)** Clinical validity for ER status by qTP was established by setting thresholds for qTP ER Negative (<12.75; green dashed line) and Positive (>14.5; red dashed line) in the training cohort (n=150) of breast cancer FFPE tissue samples with clinical ER status (by % tumor cells positive) in accompanying pathology reports (see **a**) based on the clinical IHC defined categories of ER Negative (Neg.; 0%), Low (1-10%) and Positive (Pos; >10%). Expression between the Negative and Positive thresholds were defined as qTP ER inconclusive (light gray). Desired sensitivity (sens; positive percent agreement [PPA]) and specificity (spec; negative percent agreement [NPA]) for qTP *ER* Negative/Positive status (vs. IHC Negative and Positive) was pre-specified as >95% each. Locked thresholds were then applied to the validation cohort (n=150), with box plots of qTP ER expression by clinical IHC categories shown (Neg. [n=42], Low [n=7], Pos. [n=201]; error bars as in **c**), along with PPA and NPA values and 95% confidence intervals (CI). In this validation cohort, the qTP ER inconclusive category (n=8 of 150 validation samples) correctly identified 6/7 clinical IHC ER-low samples, supporting the clinical utility of this category. **e)** Clinical validity for PR status by qTP was established by setting a threshold for qTP PR Negative (<12.3; red dashed line) in the training cohort (n=145) of breast cancer FFPE tissue samples with clinical PR status (by % tumor cells positive) in accompanying pathology reports (see **b**). Although PR does not have a “Low” clinical IHC reporting group, three clinical

IHC defined categories of PR Negative (Neg.; 0%), Low (1-10%) and Positive (Pos; >10%) were used in the training cohort to facilitate appropriate balancing of PPA and NPA in the threshold setting. As the potential clinical implications of false positive PR status, namely inappropriately considering an ER negative / HER2 negative breast cancer as hormone receptor positive (vs. triple negative) are more impactful than false negative PR status (it is unclear if ER negative/PR positive breast cancer are biologically plausible), the threshold was set to favoring NPA and pre-specified acceptable NPA (versus PR 0% IHC) of greater than 95% was set. The locked threshold was then applied to the validation cohort (n=146), with box plots of qTP PR expression by IHC categories shown (Neg. [n=80], Low [n=16], Pos. [n=50]; error bars as in **c**), along with PPA and NPA values and 95% confidence intervals (CI). **f**) Clinical validity for HER2 status by qTP was established by setting thresholds in the training cohort (n=273) of breast cancer FFPE tissue samples with clinical HER2 status (by the clinically recognized 0, 1+, 2+ or 3+ categories) in accompanying pathology reports (see **c**). As with ER, given that the clinical utility of HER2 IHC 2+ is to reflex to FISH/ISH (and StrataNGS provides *ERBB2* copy status), and the unclear validity of 0 vs. 1+ expression in retrospective samples clinically scored before the FDA approval of trastuzumab deruxtecan in HER2 1+ and 2+ (FISH/ISH negative) breast cancer, we set thresholds for qTP HER2 Low (<18.0; green dashed line) and High (>19.2; red dashed line), with expression in between those thresholds reported as qTP HER2 Inconclusive (light gray); the threshold was set by balancing desired maximum sensitivity vs. IHC 3+ with the observation that the majority of IHC 3+ tumors with the lowest qTP *HER2* expression also lacked *ERBB2* amplifications in the training cohort. Hence, desired NPA and PPA for qTP HER2 Low/High status (vs. IHC 0-1+ and 3+) was pre-specified as NPA >95% and PPA > 70%; no performance metrics for IHC 2+ samples were prespecified. Locked thresholds were then applied to the validation cohort (n=272 [including 51 IHC 2+ not formally evaluated]), with box plots of qTP HER2 expression by clinical IHC categories shown (0 [n=124, 1 of which was not evaluable for copy number status], 1+ [n=79], 2+ [n=51] or 3+ [n=18, 16 of which were amplified, 3 of which were not amplified]; error bars as in **c**), stratified by *ERBB2* copy number status (red = amplified, green = not amplified [wildtype; wt], gray = copy number status not evaluable), along with PPA and NPA values and 95% confidence intervals (CI). Notably, of the three false negative samples in the validation cohort (IHC 3+

but qTP HER2 Low), two lacked *ERBB2* amplifications by StrataNGS testing. Additionally, more than 50% (n=7) of the of the qTP HER2 inconclusive category (n=12 of 272 total validation samples) was IHC 2+, supporting the clinical utility of this category and deferral to amplification status. **g**) Although the above analyses support clinical utility of ER and PR (collectively hormone receptor [HR]) and HER2 status by qTP as the clinical utility of these biomarkers is already established, as an additional demonstration of the clinical utility of integrating qTP results with CGP results, we determined the impact of qTP HR status on *PIK3CA* mutation treatment association in patients with breast cancer (standard of care [SOC] *PIK3CA* mutations are associated with FDA-approved alpelisib + fulvestrant therapy only in patients with hormone receptor positive/HER2 negative breast cancer [green box]) from a 4-month period of consecutively tested pan-solid FFPE tumor samples (n=3,904) submitted for clinical CGP testing. As shown in the sample disposition diagram, of the 3,904 samples, 288 samples were breast cancer and met qTP and CGP QC metrics (including the final ≥20% tumor content requirement) needed to evaluate HR status, *PIK3CA* mutations and *ERBB2* copy number status, of which 31% (n=90) harbored SOC *PIK3CA* mutations associated with alpelisib therapy. Of the 90 PIK3CA mutant samples, 2 (2%) would be correctly identified as not associated with alpelisib therapy by CGP testing alone (based on the presence of an *ERBB2* amplification; pink boxes), while 11 (12%) could only be correctly identified as not associated with alpelisib therapy by integration of qTP findings (based on HR negative status; dark red box).

Figure S18. Exploratory analysis defining an Immunotherapy Response Score (IRS) ultra-low subset.

In a post-hoc, exploratory analysis in the combined discovery (n=648; pembrolizumab [pembro] treated) and validation (n=248; non-pembrolizumab PD-[L]1 treated) cohorts, we identified a threshold (<0.41) that subdivided the IRS-Low (-L) group into intermediate (IRS-L [I]) and ultra-low (IRS-L [U]) subsets. **a**) PD-(L)1 real-world progression free survival (rwPFS) in the combined cohorts stratified by IRS-High [H], IRS-L (I), and IRS-L (U) groups is shown by Kaplan Meier analysis with the Benjamini Hochberg (BH) adjusted p-value for pairwise log-rank test between the IRS-L (I) vs. IRS-L (U) groups and the

adjusted hazard ratio (HR) and p value for IRS-L (I) vs. IRS-L (U) groups shown. The Cox proportional hazard model was adjusted for age, gender, most common tumor type (NSCLC vs. other), line of therapy, type of therapy (monotherapy vs. combination therapy) and IRS (-H, -L [I], and -L [U]). The number (n) of patients, events, and median rwPFS (with 95% confidence intervals [CI]) for the analyzed groups are shown. **b**) As in a), except overall survival (OS). **c**) This three group IRS classification was applied to all 24,463 patients in the Strata Clinical Molecular Database (SCMD) with valid tumor mutation burden (TMB) and gene expression data (see **Figure 5**). IRS group distribution is shown by box plot (numbers indicated percentages). Stratification and breakdown of PD-(L)1 monotherapy approved tumor types is shown. Tumor type abbreviations: NSCLC (non-small cell lung cancer), RCC (renal cell carcinoma), NMSC (non-melanoma skin cancer), SCLC (small cell lung cancer).

Figure S19. Confirmation of the predictive nature of the Immunotherapy Response Score (IRS) Biomarker when an ultra-low subset is defined.

As shown in **Figure 3**, to establish the predictive nature of the IRS model, we assessed an internal comparator in the pembrolizumab (pembro) monotherapy cohort, consisting of the 146 patients who had received a prior line of systemic therapy prior to pembrolizumab monotherapy. Here, we subdivided the IRS-Low (-L) group into intermediate (IRS-L [I]) and ultra-low (IRS-L [U]) subsets as defined in **Supplementary Figure S18**. **a**) For each patient, rwPFS was determined for the line of systemic therapy immediately prior to pembrolizumab and the pembrolizumab monotherapy line, with rwPFS stratified by IRS status. a) Kaplan-Meier analysis of the immediately prior systemic therapy rwPFS in the IRS-High [H], IRS-L (I), and IRS-L (U) groups (overall log-rank p-value is shown). The number (n) of patients, events, and median rwPFS (with 95% confidence intervals [CI]) for each group are shown. **b-d**) Kaplan-Meier analysis of pembrolizumab monotherapy rwPFS (purple) vs. prior systemic therapy rwPFS (yellow) in the **b**) IRS-L (U) subset, **c**) the IRS-L (I) subset, and **d**) the IRS-H group of patients (log-rank p-value shown).

Figure S1

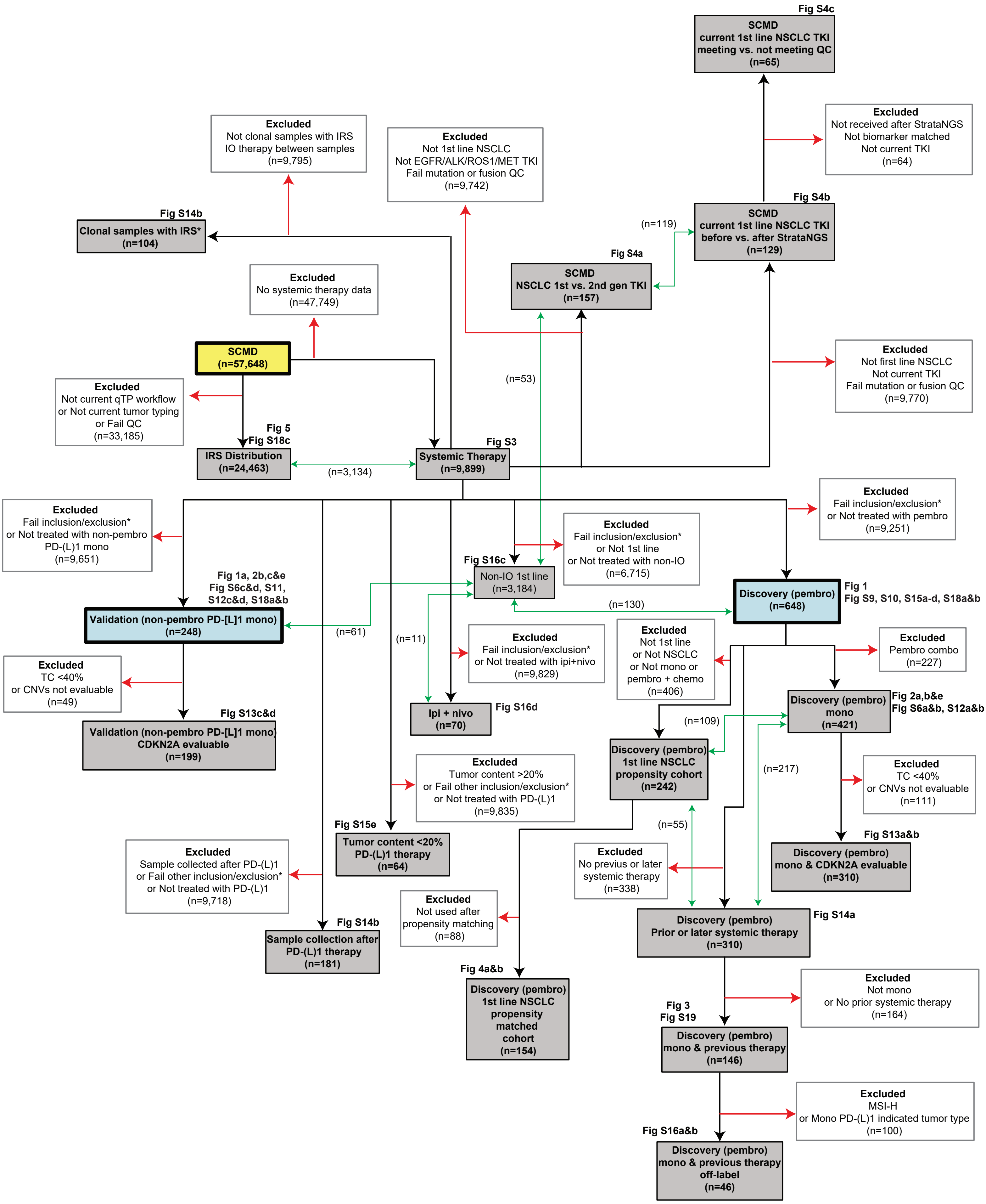

SCMD (n=57,648)
Excluded No systemic therapy data (n=47,749)
Excluded Not current qTP workflow or Not current tumor typing or Fail QC (n=33,185)
Fig 5 Fig S18c IRS Distribution (n=24,463)
(n=3,134)
Fig S3 Systemic Therapy (n=9,899)
Excluded Not clonal samples with IRS IO therapy between samples (n=9,795)
Fig S14b Clonal samples with IRS* (n=104)
Excluded Not 1st line NSCLC Not EGFR/ALK/ROS1/MET TKI Fail mutation or fusion QC (n=9,742)
Fig S4a SCMD NSCLC 1st vs. 2nd gen TKI (n=157)
(n=119)
Fig S4b SCMD current 1st line NSCLC TKI before vs. after StrataNGS (n=129)
Fig S4c SCMD current 1st line NSCLC TKI meeting vs. not meeting QC (n=65)
Excluded Not received after StrataNGS Not biomarker matched Not current TKI (n=64)
Excluded Not first line NSCLC Not current TKI Fail mutation or fusion QC (n=9,770)
(n=53)
Excluded Fail inclusion/exclusion* or Not treated with non-pembro PD-(L)1 mono (n=9,651)
Fig 1a, 2b,c&e Fig S6c&d, S11, S12c&d, S18a&b Validation (non-pembro PD-[L]1 mono) (n=248)
Excluded TC <40% or CNVs not evaluable (n=49)
Fig S13c&d Validation (non-pembro PD-[L]1 mono) CDKN2A evaluable (n=199)
(n=61)
Fig S16c Non-IO 1st line (n=3,184)
Excluded Fail inclusion/exclusion* or Not 1st line or Not treated with non-IO (n=6,715)
(n=130)
Excluded Fail inclusion/exclusion* or Not treated with pembro (n=9,251)
Discovery (pembro) (n=648) Fig 1 Fig S9, S10, S15a-d, S18a&b
(n=11)
Excluded Fail inclusion/exclusion* or Not treated with ipi+nivo (n=9,829)
Ipi + nivo (n=70) Fig S16d
Excluded Not 1st line or Not NSCLC or Not mono or pembro + chemo (n=406)
Excluded Pembro combo (n=227)
Discovery (pembro) mono (n=421) Fig 2a,b&e Fig S6a&b, S12a&b
(n=109)
Discovery (pembro) 1st line NSCLC propensity cohort (n=242)
(n=217)
Excluded TC <40% or CNVs not evaluable (n=111)
Excluded Tumor content >20% or Fail other inclusion/exclusion* or Not treated with PD-(L)1 (n=9,835)
Fig S15e Tumor content <20% PD-(L)1 therapy (n=64)
(n=55)
Excluded No previus or later systemic therapy (n=338)
Fig S13a&b Discovery (pembro) mono & CDKN2A evaluable (n=310)
Excluded Sample collected after PD-(L)1 or Fail other inclusion/exclusion* or Not treated with PD-(L)1 (n=9,718)
Fig S14b Sample collection after PD-(L)1 therapy (n=181)
Excluded Not used after propensity matching (n=88)
Fig S14a Discovery (pembro) Prior or later systemic therapy (n=310)
Fig 4a&b Discovery (pembro) 1st line NSCLC propensity matched cohort (n=154)
Excluded Not mono or No prior systemic therapy (n=164)
Fig 3 Fig S19 Discovery (pembro) mono & previous therapy (n=146)
Excluded MSI-H or Mono PD-(L)1 indicated tumor type (n=100)
Fig S16a&b Discovery (pembro) mono & previous therapy off-label (n=46)

Figure S2

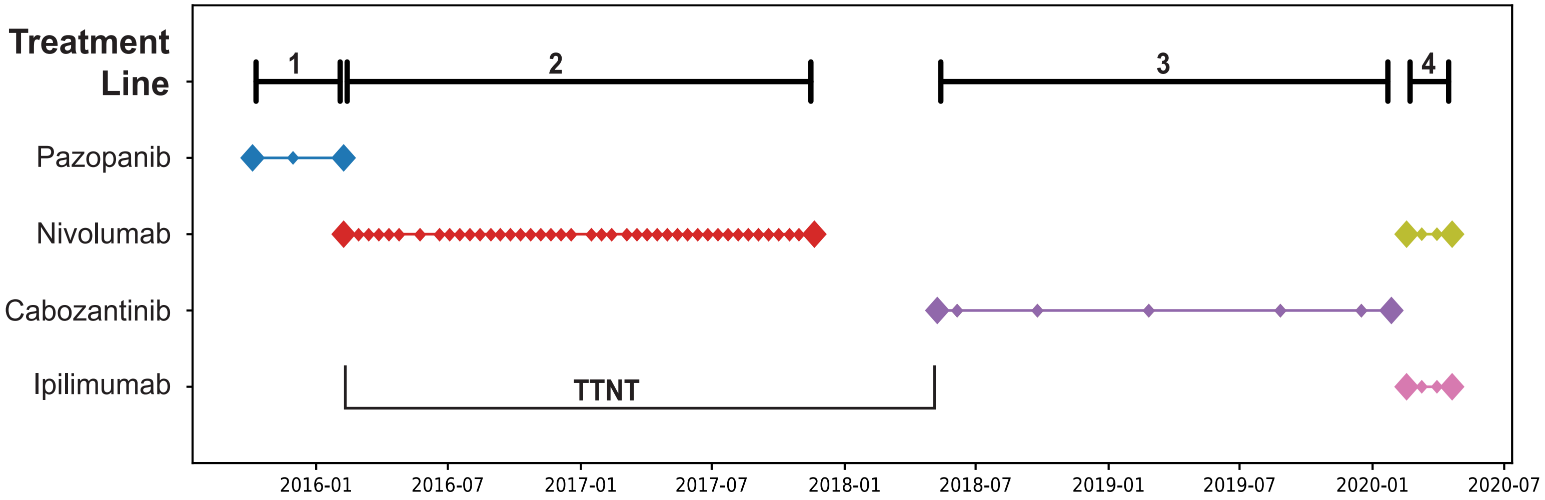
Treatment Line
1
2
3
4
Pazopanib
Nivolumab
Cabozantinib
Ipilimumab
TTNT
2016-01
2016-07
2017-01
2017-07
2018-01
2018-07
2019-01
2019-07
2020-01
2020-07

Figure S3

Line of Systemic Therapy

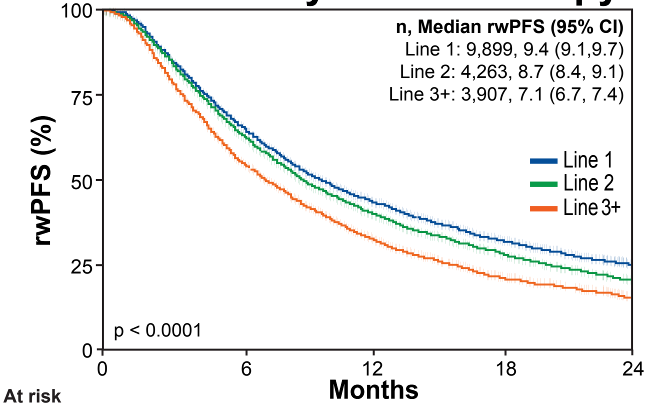

At risk

Line 1	9,899	4,697	2,450	1,376	858
Line 2	4,263	2,121	1055	597	340
Line 3+	3,907	1,681	770	355	183

Figure S4

a

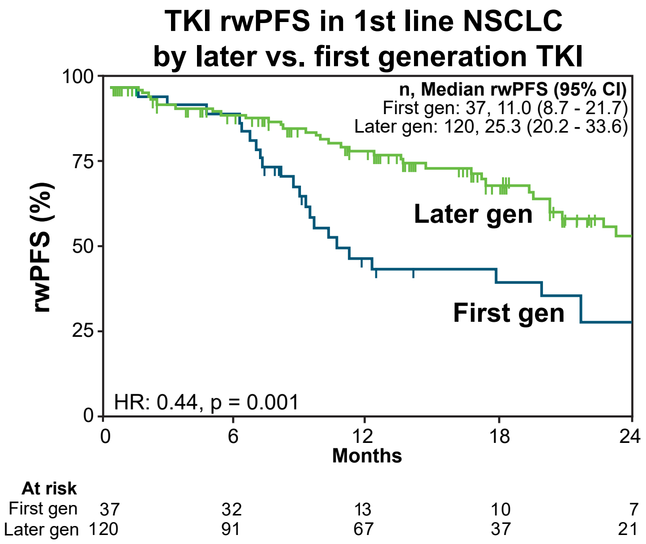

b

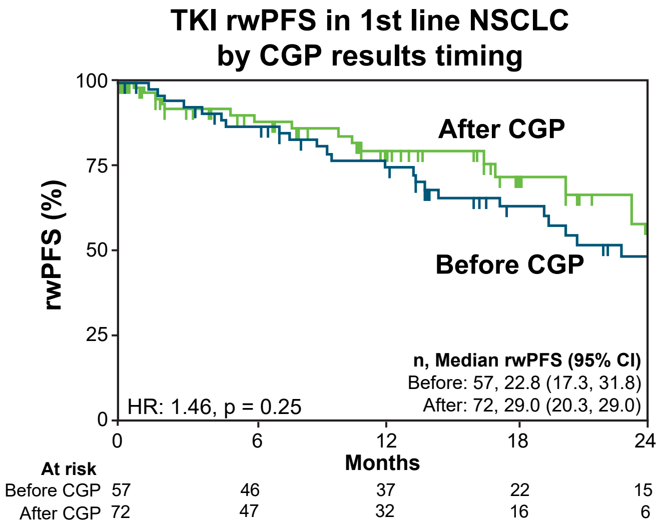

c

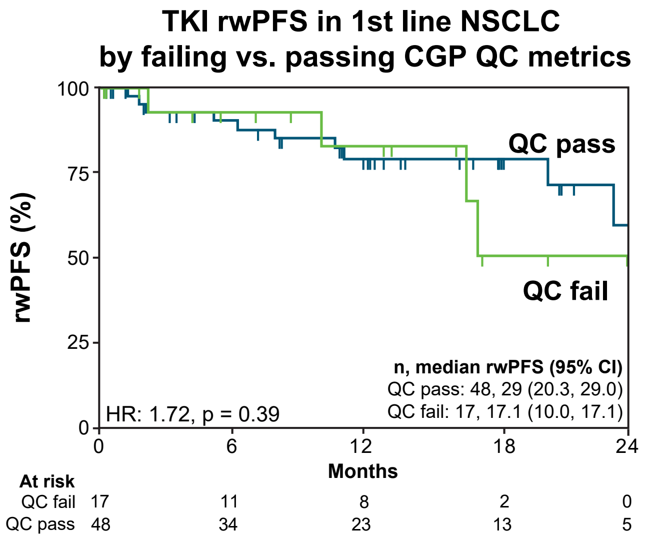

Figure S5

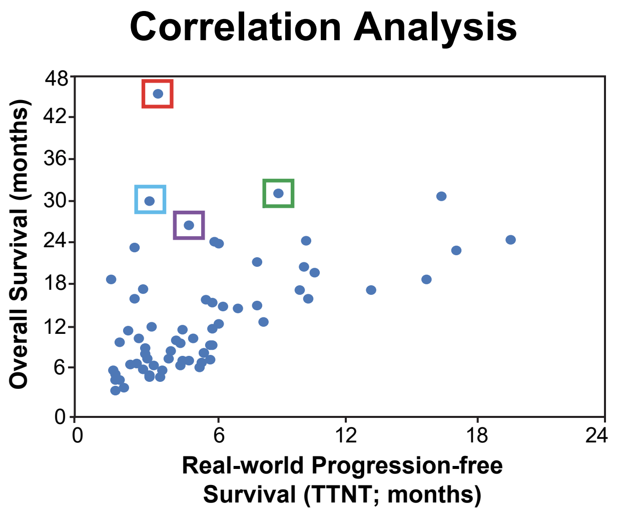
Correlation Analysis
Overall Survival (months)
48
42
36
30
24
18
12
6
0
0
6
12
18
24
Real-world Progression-free
Survival (TTNT; months)

Figure S6

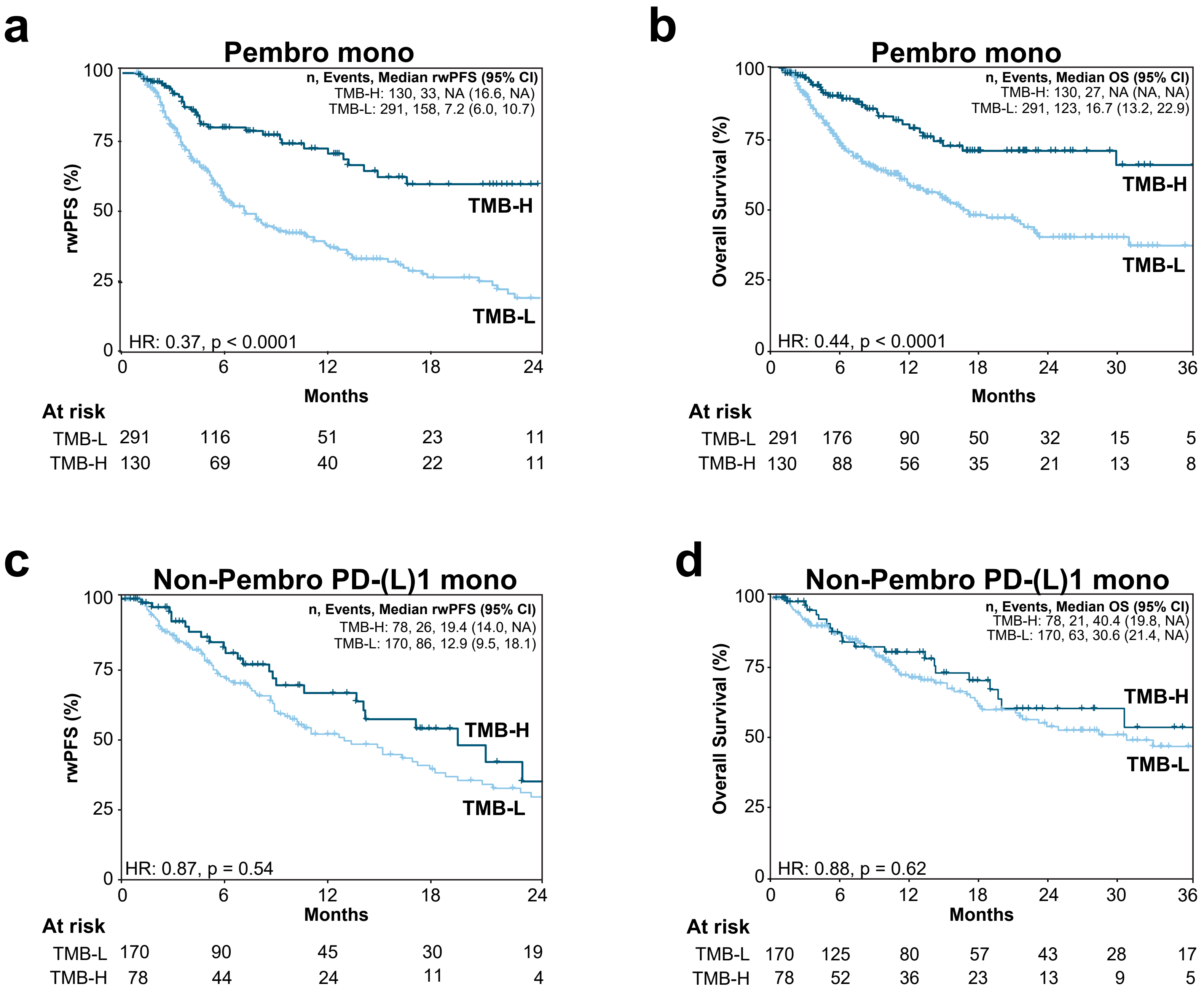
a
Pembro mono
n, Events, Median rwPFS (95% CI)
TMB-H: 130, 33, NA (16.6, NA)
TMB-L: 291, 158, 7.2 (6.0, 10.7)
rwPFS (%)
TMB-H
TMB-L
HR: 0.37, p < 0.0001
Months
At risk
TMB-L 291 116 51 23 11
TMB-H 130 69 40 22 11
b
Pembro mono
n, Events, Median OS (95% CI)
TMB-H: 130, 27, NA (NA, NA)
TMB-L: 291, 123, 16.7 (13.2, 22.9)
Overall Survival (%)
TMB-H
TMB-L
HR: 0.44, p < 0.0001
Months
At risk
TMB-L 291 176 90 50 32 15 5
TMB-H 130 88 56 35 21 13 8
c
Non-Pembro PD-(L)1 mono
n, Events, Median rwPFS (95% CI)
TMB-H: 78, 26, 19.4 (14.0, NA)
TMB-L: 170, 86, 12.9 (9.5, 18.1)
rwPFS (%)
TMB-H
TMB-L
HR: 0.87, p = 0.54
Months
At risk
TMB-L 170 90 45 30 19
TMB-H 78 44 24 11 4
d
Non-Pembro PD-(L)1 mono
n, Events, Median OS (95% CI)
TMB-H: 78, 21, 40.4 (19.8, NA)
TMB-L: 170, 63, 30.6 (21.4, NA)
Overall Survival (%)
TMB-H
TMB-L
HR: 0.88, p = 0.62
Months
At risk
TMB-L 170 125 80 57 43 28 17
TMB-H 78 52 36 23 13 9 5

Figure S7

a

Type	Gene	Average (TPM)	Coefficient of Variation All (n=20,841)	Cell line (n=935)	Normal (n=9,966)	TCGA (n=9,940)
TCGA stable	***SLC4A1AP***	16.7	46.2%	50.0%	47.6%	40.3%
TCGA stable	***CTCF***	18.1	45.6%	44.8%	42.1%	40.9%
TCGA stable	***EIF2B1***	27.8	42.9%	37.8%	42.4%	38.7%
TCGA stable	*ELAVL1*	31.4	53.6%	34.9%	47.8%	41.6%
TCGA stable	***CIAO1***	36.0	44.3%	30.9%	36.9%	40.7%
TCGA stable	***GGNBP2***	41.3	44.7%	33.4%	45.0%	39.2%
TCGA stable	*FAM32A*	58.8	43.7%	34.9%	41.3%	38.1%
TCGA stable	*KHDRBS1*	61.1	50.5%	37.3%	46.7%	39.8%
TCGA stable	*DHX9*	70.4	47.2%	39.1%	43.9%	39.2%
TCGA stable	*ZNF207*	79.0	50.2%	28.1%	51.0%	48.4%
TCGA stable	*TARDBP*	112.5	44.0%	30.5%	42.4%	36.4%
TCGA stable	*PTBP1*	120.1	63.0%	35.7%	55.8%	42.1%
TCGA stable	*SF3B2*	126.7	43.4%	34.7%	45.8%	40.4%
TCGA stable	*EWSR1*	138.2	42.6%	31.6%	42.1%	38.7%
TCGA stable	*EIF4H*	169.3	41.8%	31.2%	38.6%	38.7%
TCGA stable	*HNRNPL*	187.4	43.8%	33.2%	40.4%	34.6%
TCGA stable	*PCBP1*	213.2	41.8%	30.6%	47.4%	35.9%
TCGA stable	*HNRNPK*	281.2	46.4%	27.5%	40.1%	39.2%
OPA positive	***ITGB7***	6.4	840.4%	945.9%	191.0%	139.6%
OPA positive	*TBP*	11.1	53.2%	40.4%	57.0%	43.7%
OPA positive	***HMBS***	18.1	83.7%	96.1%	67.9%	54.7%
OPA positive	*MRPL13*	34.5	79.2%	61.6%	46.6%	64.3%
OPA positive	***MYC***	59.3	128.4%	126.3%	145.9%	102.9%
OPA positive	*LRP1*	93.7	106.0%	209.6%	86.5%	120.0%
HK gene	*GAPDH*	2841.4	82.8%	49.6%	90.7%	68.0%

c

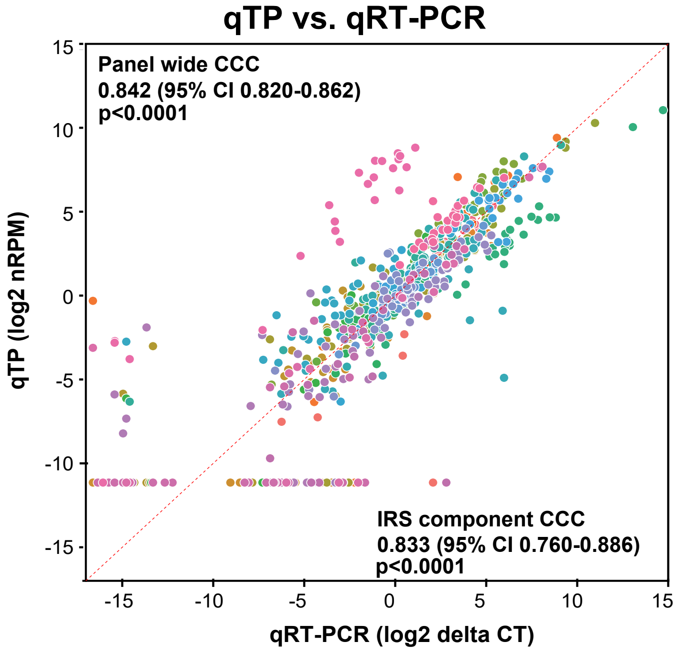

b

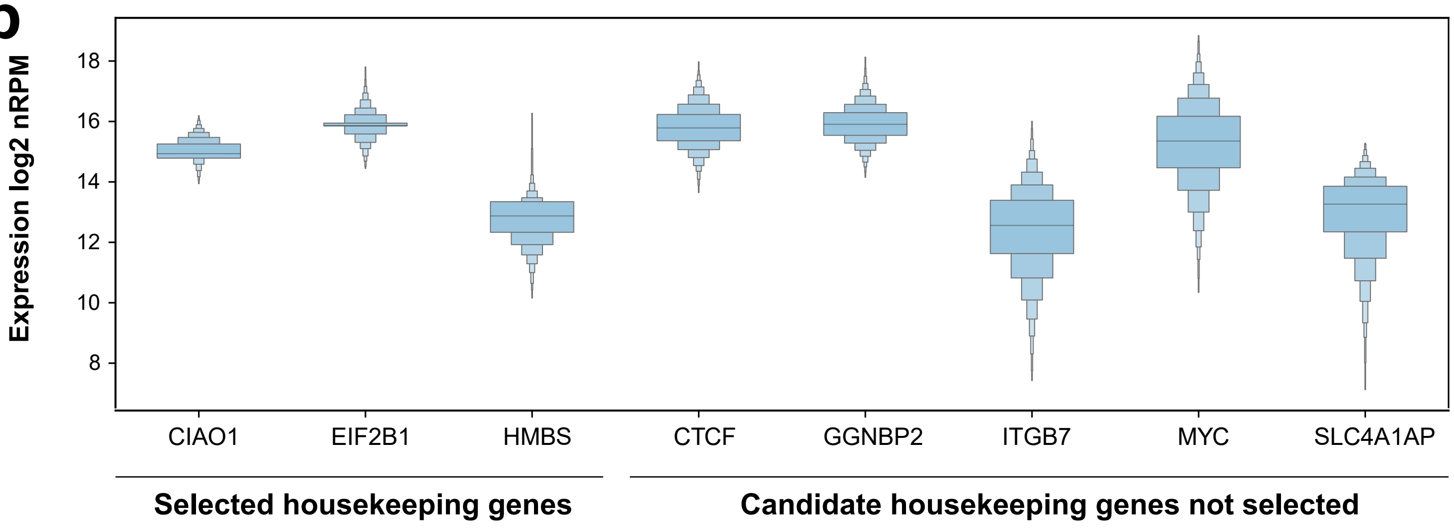

d

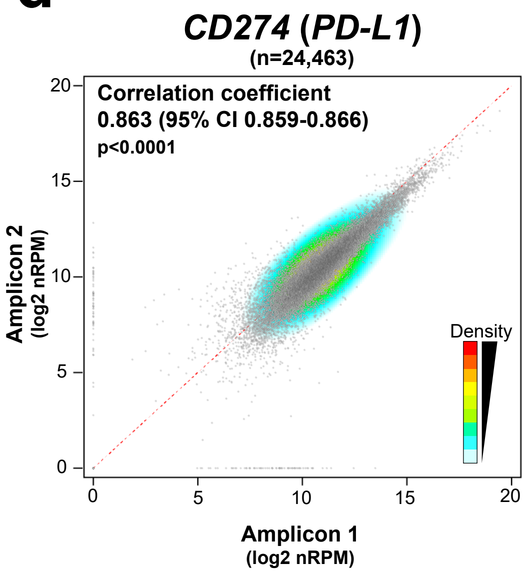

e

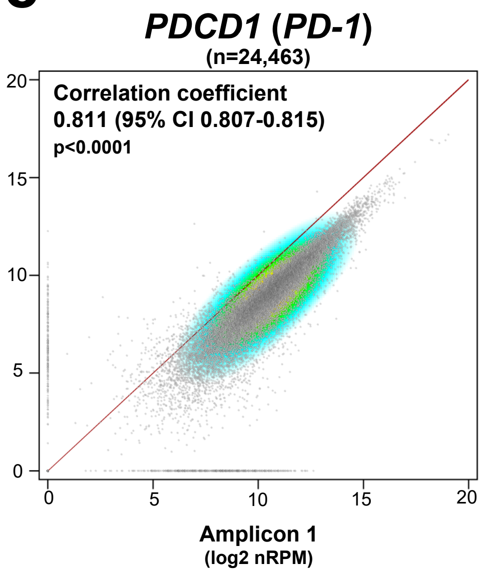

f

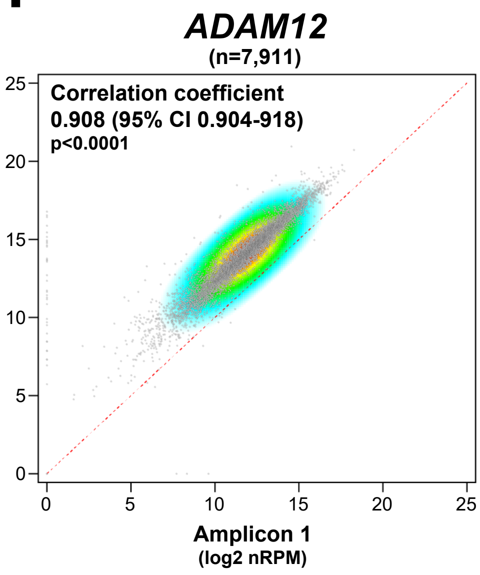

Figure S8

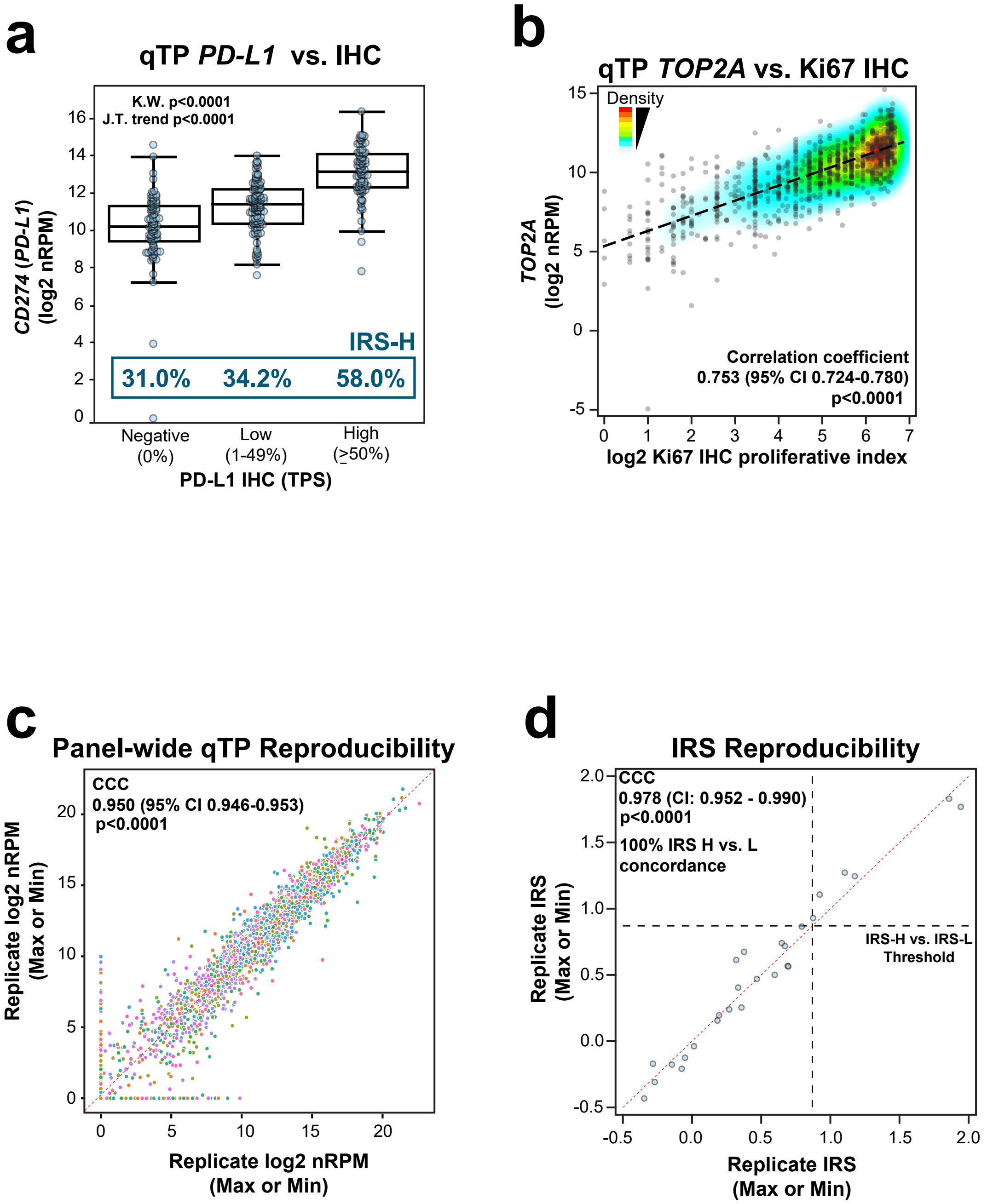
a
qTP PD-L1 vs. IHC
K.W. p<0.0001
J.T. trend p<0.0001
CD274 (PD-L1) (log2 nRPM)
IRS-H
31.0%
34.2%
58.0%
Negative (0%)
Low (1-49%)
High (≥50%)
PD-L1 IHC (TPS)
b
qTP TOP2A vs. Ki67 IHC
Density
TOP2A (log2 nRPM)
Correlation coefficient
0.753 (95% CI 0.724-0.780)
p<0.0001
log2 Ki67 IHC proliferative index
c
Panel-wide qTP Reproducibility
CCC
0.950 (95% CI 0.946-0.953)
p<0.0001
Replicate log2 nRPM (Max or Min)
Replicate log2 nRPM (Max or Min)
d
IRS Reproducibility
CCC
0.978 (CI: 0.952 - 0.990)
p<0.0001
100% IRS H vs. L concordance
IRS-H vs. IRS-L Threshold
Replicate IRS (Max or Min)
Replicate IRS (Max or Min)

Figure S9

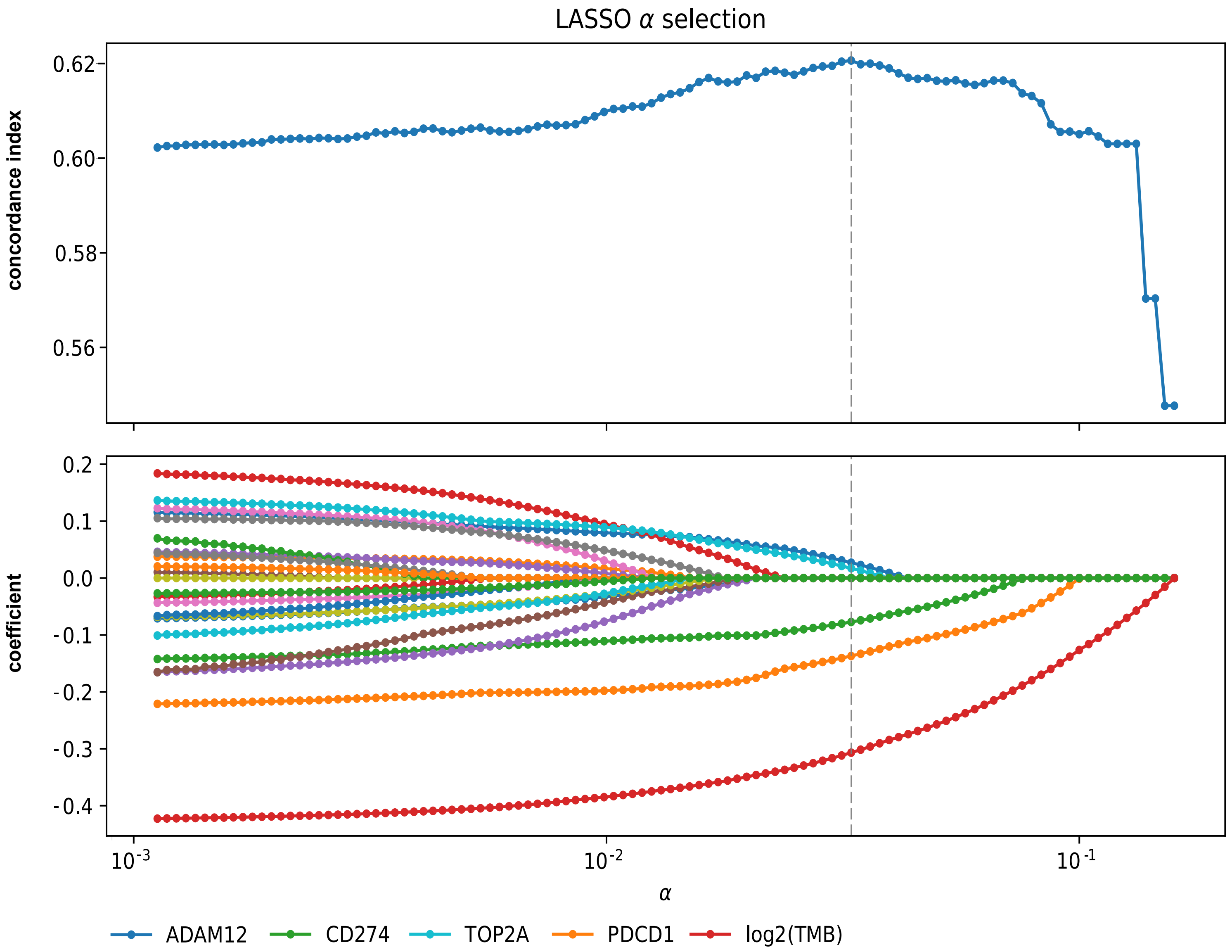

LASSO α selection
concordance index
0.62
0.60
0.58
0.56
coefficient
0.2
0.1
0.0
-0.1
-0.2
-0.3
-0.4
10^{-3}
10^{-2}
10^{-1}
α
ADAM12
CD274
TOP2A
PDCD1
log2(TMB)

Figure S10

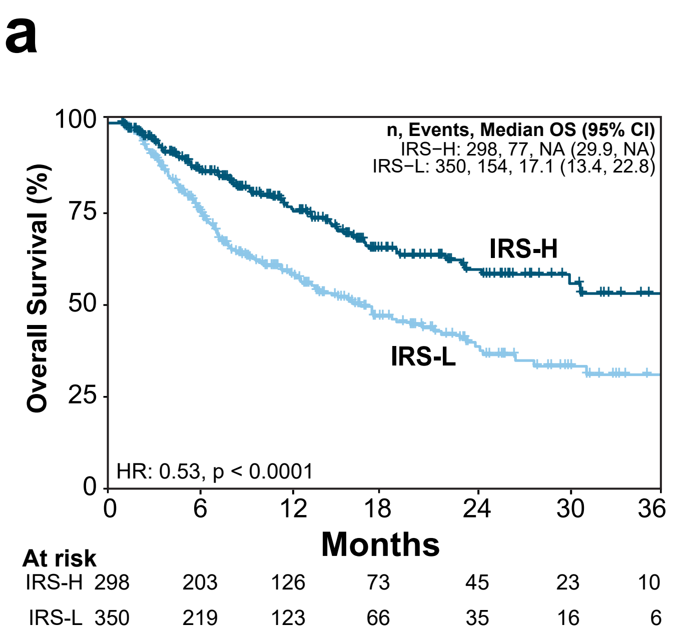
a
100
75
50
25
0
Overall Survival (%)
n, Events, Median OS (95% CI)
IRS−H: 298, 77, NA (29.9, NA)
IRS−L: 350, 154, 17.1 (13.4, 22.8)
IRS-H
IRS-L
HR: 0.53, p < 0.0001
0
6
12
18
24
30
36
Months
At risk
IRS-H 298 203 126 73 45 23 10
IRS-L 350 219 123 66 35 16 6

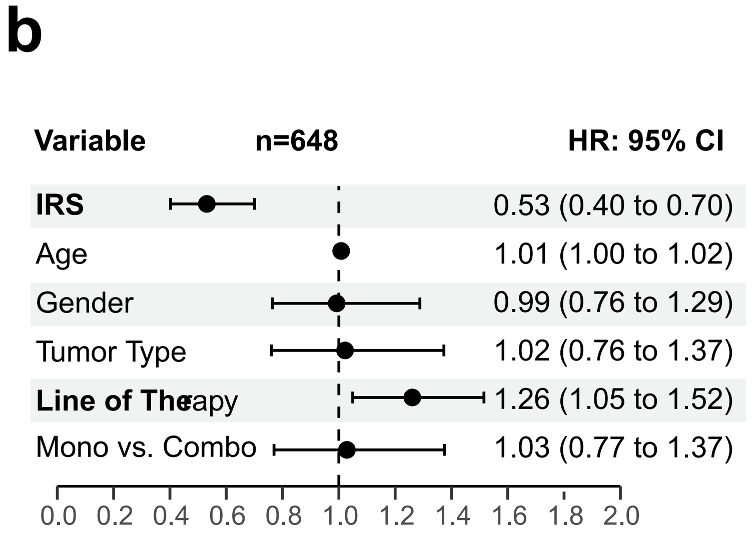
b
Variable
n=648
HR: 95% CI
IRS
0.53 (0.40 to 0.70)
Age
1.01 (1.00 to 1.02)
Gender
0.99 (0.76 to 1.29)
Tumor Type
1.02 (0.76 to 1.37)
Line of Therapy
1.26 (1.05 to 1.52)
Mono vs. Combo
1.03 (0.77 to 1.37)
0.0 0.2 0.4 0.6 0.8 1.0 1.2 1.4 1.6 1.8 2.0

Figure S11

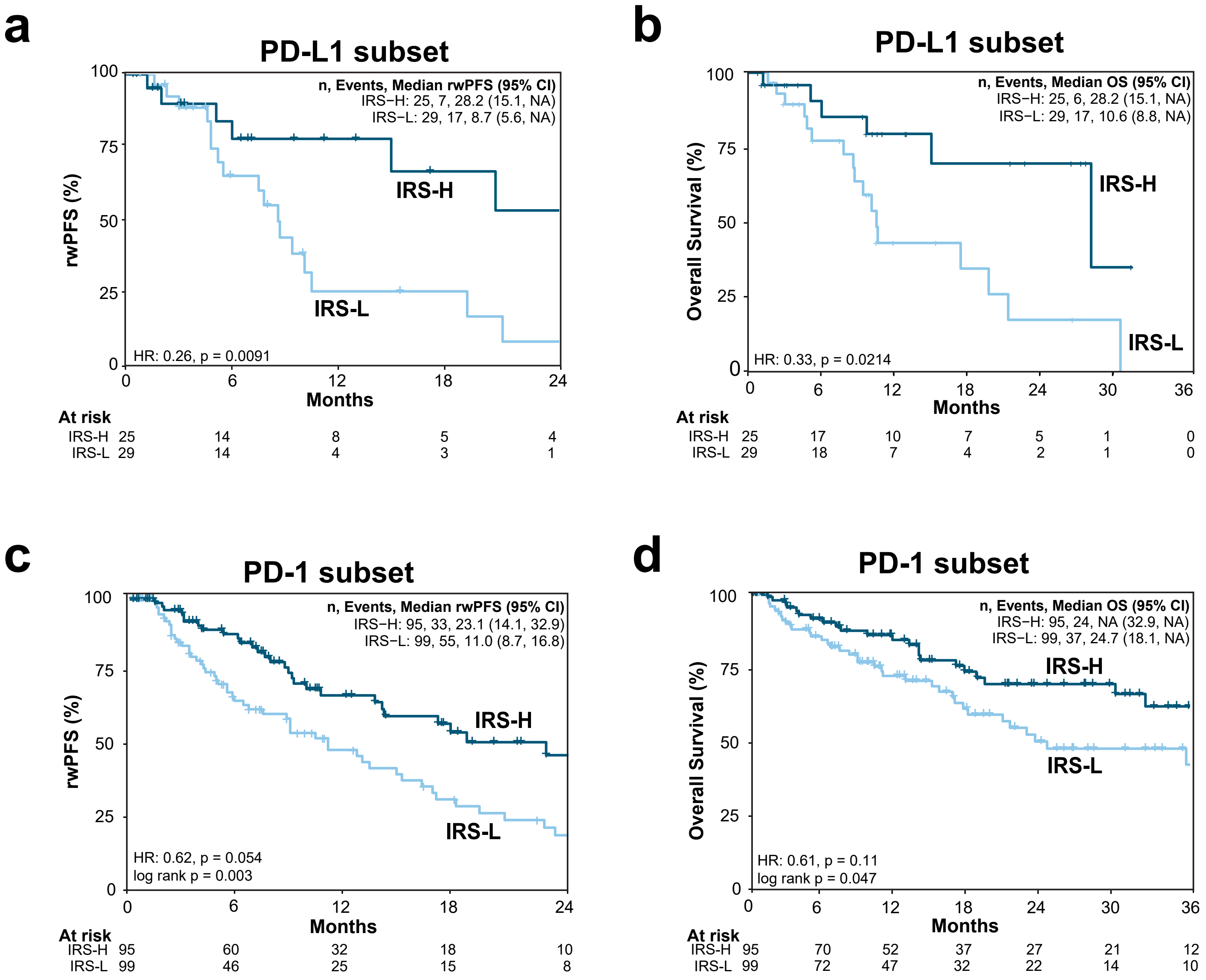
a
PD-L1 subset
n, Events, Median rwPFS (95% CI)
IRS−H: 25, 7, 28.2 (15.1, NA)
IRS−L: 29, 17, 8.7 (5.6, NA)
IRS-H
IRS-L
HR: 0.26, p = 0.0091
rwPFS (%)
Months
At risk
IRS-H 25 14 8 5 4
IRS-L 29 14 4 3 1
b
PD-L1 subset
n, Events, Median OS (95% CI)
IRS−H: 25, 6, 28.2 (15.1, NA)
IRS−L: 29, 17, 10.6 (8.8, NA)
IRS-H
IRS-L
HR: 0.33, p = 0.0214
Overall Survival (%)
Months
At risk
IRS-H 25 17 10 7 5 1 0
IRS-L 29 18 7 4 2 1 0
c
PD-1 subset
n, Events, Median rwPFS (95% CI)
IRS−H: 95, 33, 23.1 (14.1, 32.9)
IRS−L: 99, 55, 11.0 (8.7, 16.8)
IRS-H
IRS-L
HR: 0.62, p = 0.054
log rank p = 0.003
rwPFS (%)
Months
At risk
IRS-H 95 60 32 18 10
IRS-L 99 46 25 15 8
d
PD-1 subset
n, Events, Median OS (95% CI)
IRS−H: 95, 24, NA (32.9, NA)
IRS−L: 99, 37, 24.7 (18.1, NA)
IRS-H
IRS-L
HR: 0.61, p = 0.11
log rank p = 0.047
Overall Survival (%)
Months
At risk
IRS-H 95 70 52 37 27 21 12
IRS-L 99 72 47 32 22 14 10

Figure S12

a

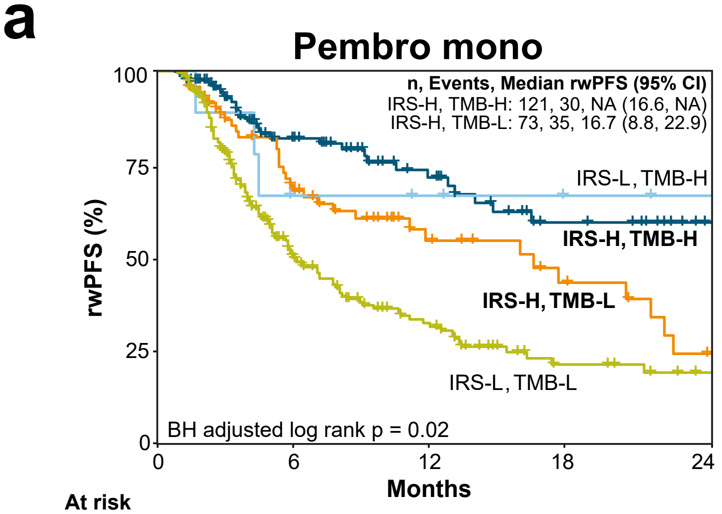

At risk	0	6	12	18	24
IRS-H ,TMB-H	121	64	36	19	10
IRS-H , TMB-L	73	43	18	11	5
IRS-L ,TMB-H	9	5	4	3	1
IRS-L , TMB-L	218	73	33	12	6

b

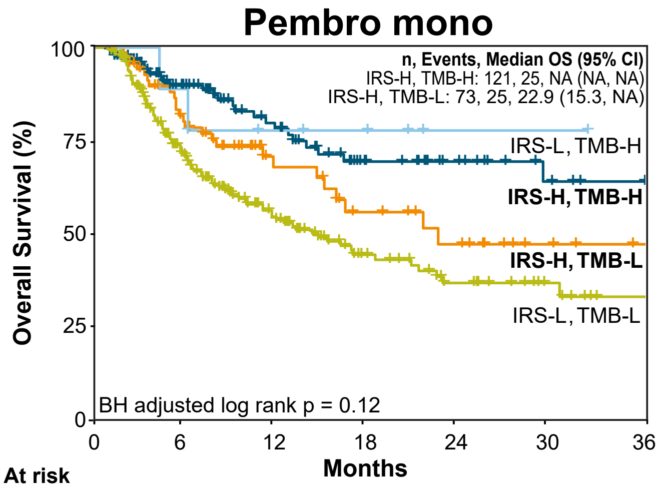

At risk	0	6	12	18	24	30	36
IRS-H ,TMB-H	121	80	51	31	20	12	8
IRS-H , TMB-L	73	50	24	16	11	4	1
IRS-L ,TMB-H	9	8	5	4	1	1	0
IRS-L , TMB-L	218	126	66	34	21	11	4

c

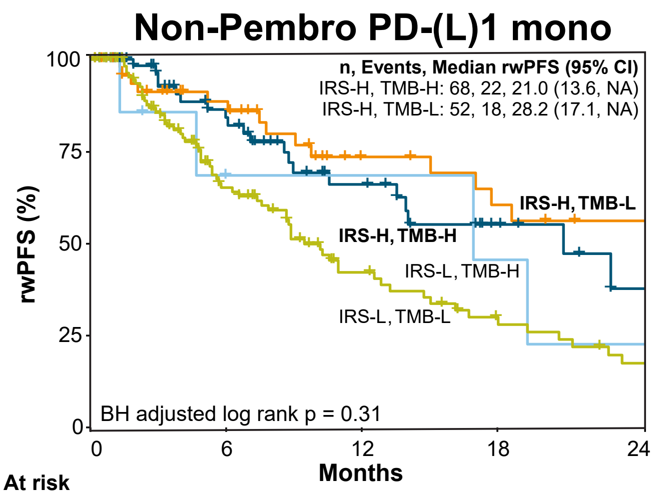

At risk	0	6	12	18	24
IRS-H ,TMB-H	68	40	21	9	3
IRS-H , TMB-L	52	34	19	14	11
IRS-L ,TMB-H	10	4	3	2	1
IRS-L , TMB-L	118	56	26	16	8

d

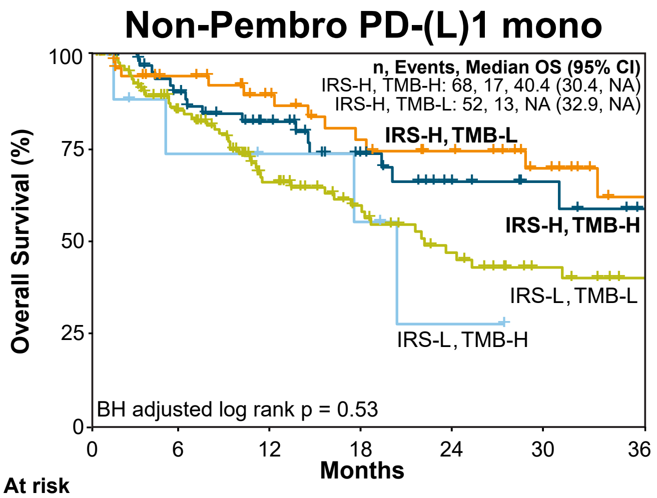

At risk	0	6	12	18	24	30	36
IRS-H ,TMB-H	68	47	32	20	12	9	5
IRS-H , TMB-L	52	40	30	24	20	13	7
IRS-L ,TMB-H	10	5	4	3	1	0	0
IRS-L , TMB-L	118	85	50	33	23	15	10

Figure S13

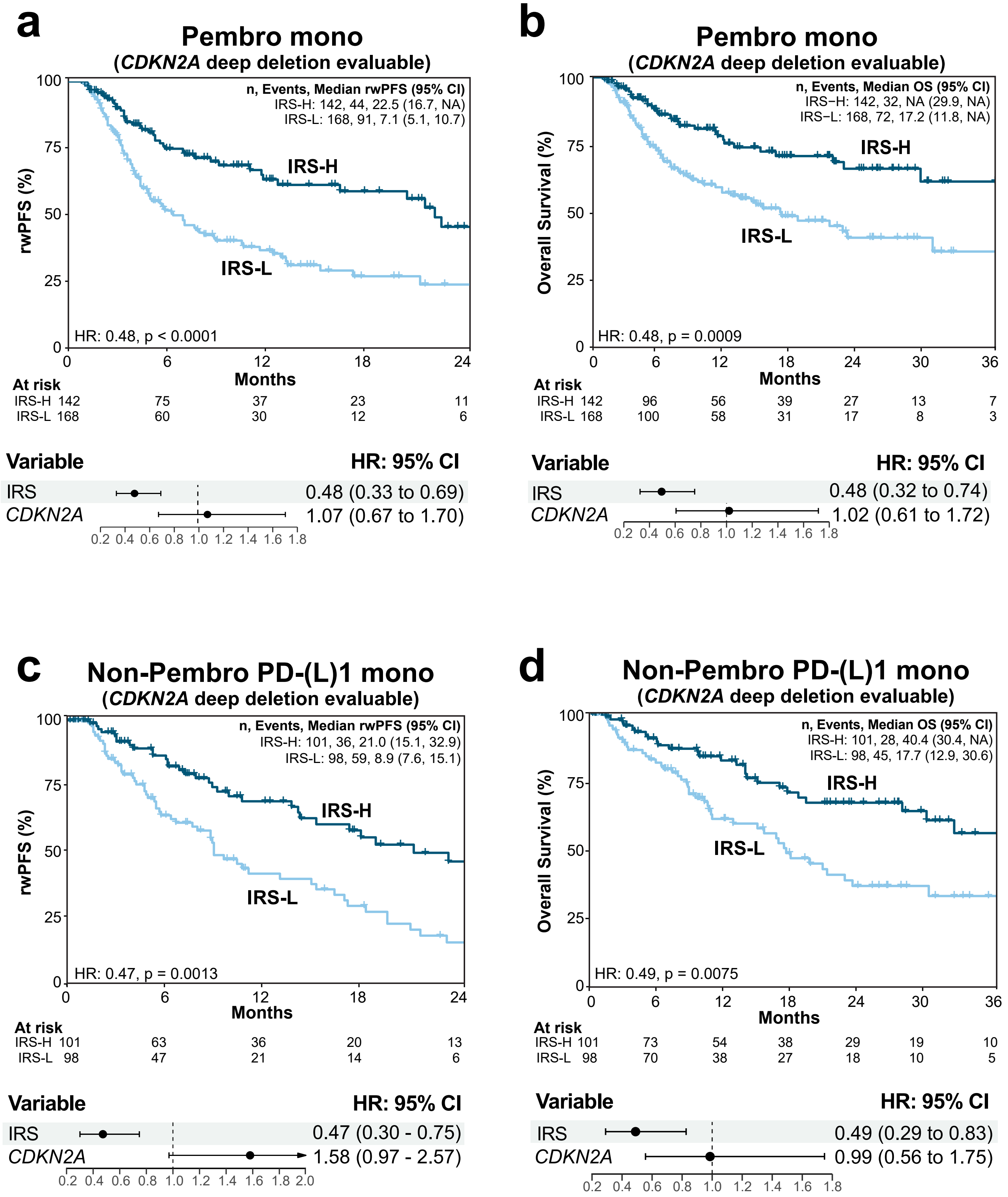
a
Pembro mono
(CDKN2A deep deletion evaluable)
n, Events, Median rwPFS (95% CI)
IRS-H: 142, 44, 22.5 (16.7, NA)
IRS-L: 168, 91, 7.1 (5.1, 10.7)
IRS-H
IRS-L
HR: 0.48, p < 0.0001
rwPFS (%)
Months
At risk
IRS-H 142 75 37 23 11
IRS-L 168 60 30 12 6
Variable
HR: 95% CI
IRS 0.48 (0.33 to 0.69)
CDKN2A 1.07 (0.67 to 1.70)
b
Pembro mono
(CDKN2A deep deletion evaluable)
n, Events, Median OS (95% CI)
IRS−H: 142, 32, NA (29.9, NA)
IRS−L: 168, 72, 17.2 (11.8, NA)
IRS-H
IRS-L
HR: 0.48, p = 0.0009
Overall Survival (%)
Months
At risk
IRS-H 142 96 56 39 27 13 7
IRS-L 168 100 58 31 17 8 3
Variable
HR: 95% CI
IRS 0.48 (0.32 to 0.74)
CDKN2A 1.02 (0.61 to 1.72)
c
Non-Pembro PD-(L)1 mono
(CDKN2A deep deletion evaluable)
n, Events, Median rwPFS (95% CI)
IRS-H: 101, 36, 21.0 (15.1, 32.9)
IRS-L: 98, 59, 8.9 (7.6, 15.1)
IRS-H
IRS-L
HR: 0.47, p = 0.0013
rwPFS (%)
Months
At risk
IRS-H 101 63 36 20 13
IRS-L 98 47 21 14 6
Variable
HR: 95% CI
IRS 0.47 (0.30 - 0.75)
CDKN2A 1.58 (0.97 - 2.57)
d
Non-Pembro PD-(L)1 mono
(CDKN2A deep deletion evaluable)
n, Events, Median OS (95% CI)
IRS-H: 101, 28, 40.4 (30.4, NA)
IRS-L: 98, 45, 17.7 (12.9, 30.6)
IRS-H
IRS-L
HR: 0.49, p = 0.0075
Overall Survival (%)
Months
At risk
IRS-H 101 73 54 38 29 19 10
IRS-L 98 70 38 27 18 10 5
Variable
HR: 95% CI
IRS 0.49 (0.29 to 0.83)
CDKN2A 0.99 (0.56 to 1.75)

Figure S14

a

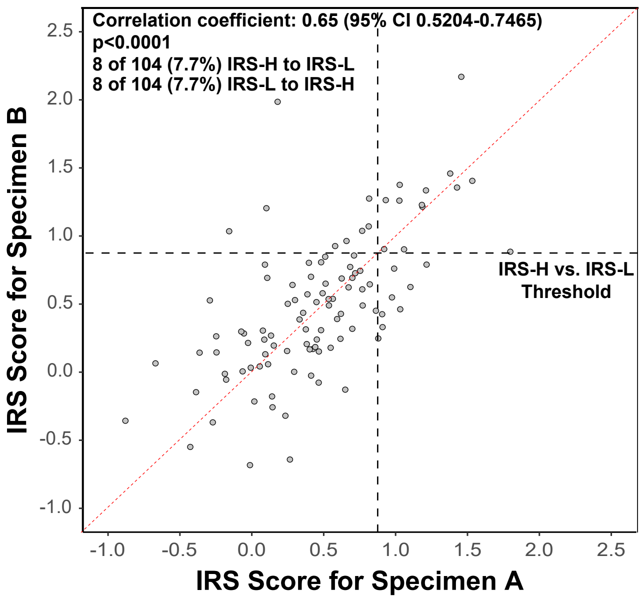

b

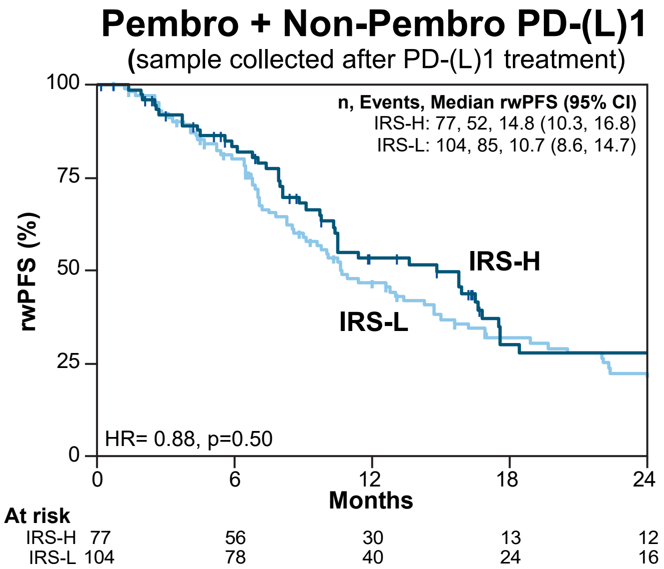

Figure S15

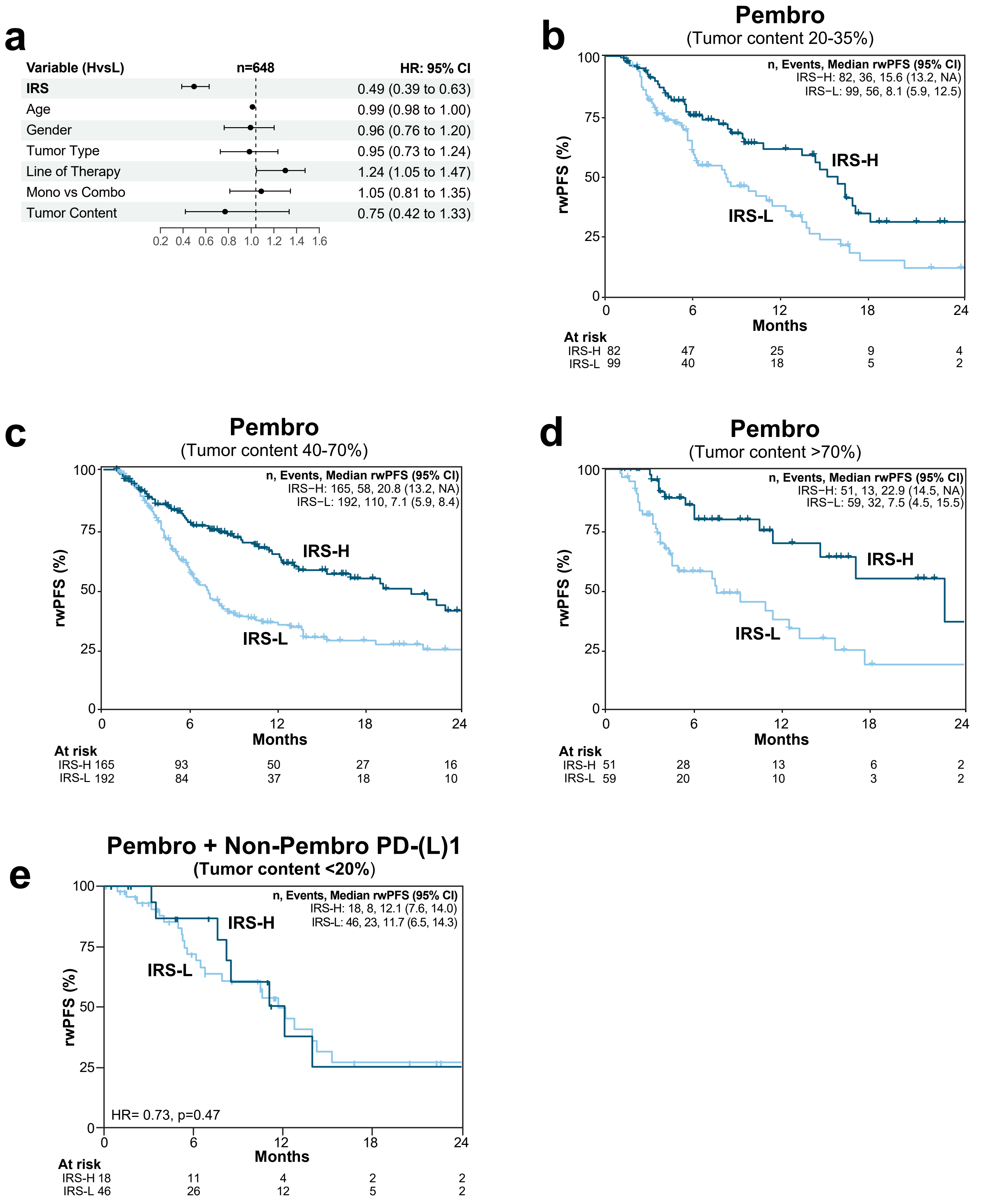
a
Variable (HvsL)
n=648
HR: 95% CI
IRS
0.49 (0.39 to 0.63)
Age
0.99 (0.98 to 1.00)
Gender
0.96 (0.76 to 1.20)
Tumor Type
0.95 (0.73 to 1.24)
Line of Therapy
1.24 (1.05 to 1.47)
Mono vs Combo
1.05 (0.81 to 1.35)
Tumor Content
0.75 (0.42 to 1.33)
0.2 0.4 0.6 0.8 1.0 1.2 1.4 1.6
b
Pembro
(Tumor content 20-35%)
n, Events, Median rwPFS (95% CI)
IRS−H: 82, 36, 15.6 (13.2, NA)
IRS−L: 99, 56, 8.1 (5.9, 12.5)
IRS-H
IRS-L
rwPFS (%)
Months
At risk
IRS-H 82 47 25 9 4
IRS-L 99 40 18 5 2
c
Pembro
(Tumor content 40-70%)
n, Events, Median rwPFS (95% CI)
IRS−H: 165, 58, 20.8 (13.2, NA)
IRS−L: 192, 110, 7.1 (5.9, 8.4)
IRS-H
IRS-L
rwPFS (%)
Months
At risk
IRS-H 165 93 50 27 16
IRS-L 192 84 37 18 10
d
Pembro
(Tumor content >70%)
n, Events, Median rwPFS (95% CI)
IRS−H: 51, 13, 22.9 (14.5, NA)
IRS−L: 59, 32, 7.5 (4.5, 15.5)
IRS-H
IRS-L
rwPFS (%)
Months
At risk
IRS-H 51 28 13 6 2
IRS-L 59 20 10 3 2
e
Pembro + Non-Pembro PD-(L)1
(Tumor content <20%)
n, Events, Median rwPFS (95% CI)
IRS-H: 18, 8, 12.1 (7.6, 14.0)
IRS-L: 46, 23, 11.7 (6.5, 14.3)
IRS-H
IRS-L
HR= 0.73, p=0.47
rwPFS (%)
Months
At risk
IRS-H 18 11 4 2 2
IRS-L 46 26 12 5 2

Figure S16

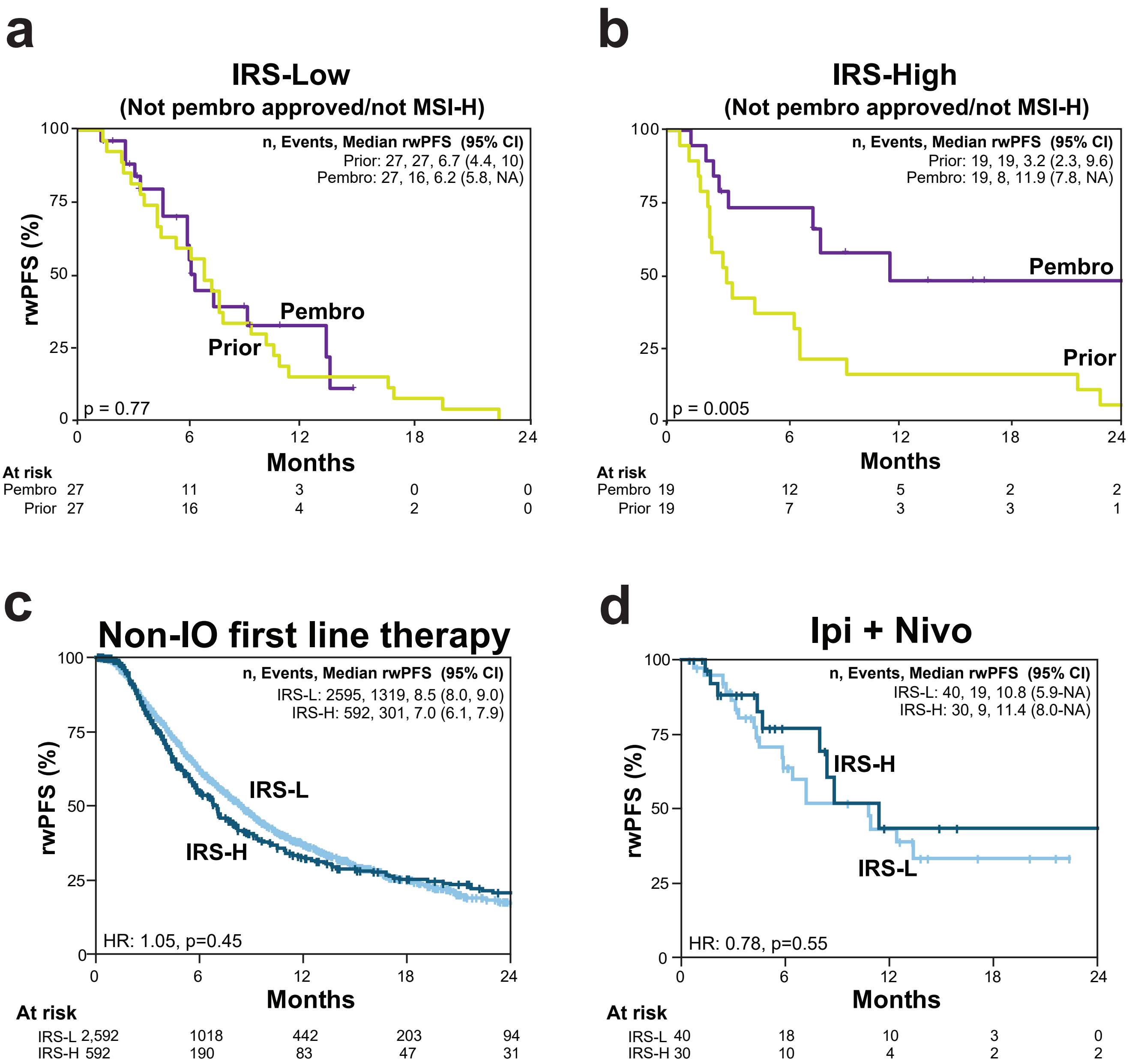
a
IRS-Low
(Not pembro approved/not MSI-H)
n, Events, Median rwPFS (95% CI)
Prior: 27, 27, 6.7 (4.4, 10)
Pembro: 27, 16, 6.2 (5.8, NA)
Pembro
Prior
p = 0.77
rwPFS (%)
Months
At risk
Pembro 27 11 3 0 0
Prior 27 16 4 2 0
b
IRS-High
(Not pembro approved/not MSI-H)
n, Events, Median rwPFS (95% CI)
Prior: 19, 19, 3.2 (2.3, 9.6)
Pembro: 19, 8, 11.9 (7.8, NA)
Pembro
Prior
p = 0.005
Months
At risk
Pembro 19 12 5 2 2
Prior 19 7 3 3 1
c
Non-IO first line therapy
n, Events, Median rwPFS (95% CI)
IRS-L: 2595, 1319, 8.5 (8.0, 9.0)
IRS-H: 592, 301, 7.0 (6.1, 7.9)
IRS-L
IRS-H
HR: 1.05, p=0.45
rwPFS (%)
Months
At risk
IRS-L 2,592 1018 442 203 94
IRS-H 592 190 83 47 31
d
Ipi + Nivo
n, Events, Median rwPFS (95% CI)
IRS-L: 40, 19, 10.8 (5.9-NA)
IRS-H: 30, 9, 11.4 (8.0-NA)
IRS-H
IRS-L
HR: 0.78, p=0.55
rwPFS (%)
Months
At risk
IRS-L 40 18 10 3 0
IRS-H 30 10 4 2 2

Figure S17

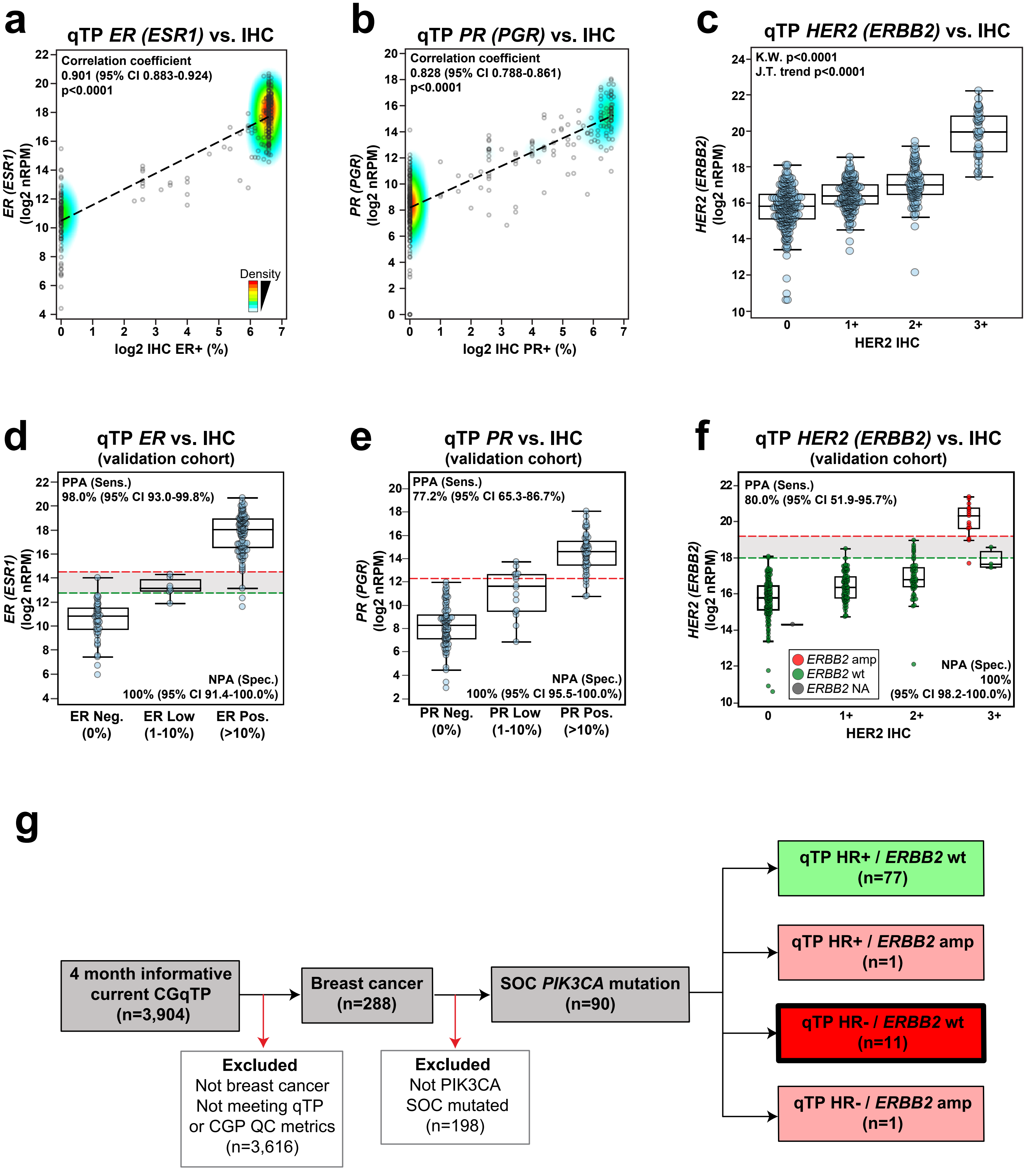
a
qTP ER (ESR1) vs. IHC
Correlation coefficient
0.901 (95% CI 0.883-0.924)
p<0.0001
Density
ER (ESR1) (log2 nRPM)
log2 IHC ER+ (%)
b
qTP PR (PGR) vs. IHC
Correlation coefficient
0.828 (95% CI 0.788-0.861)
p<0.0001
PR (PGR) (log2 nRPM)
log2 IHC PR+ (%)
c
qTP HER2 (ERBB2) vs. IHC
K.W. p<0.0001
J.T. trend p<0.0001
HER2 (ERBB2) (log2 nRPM)
HER2 IHC
d
qTP ER vs. IHC (validation cohort)
PPA (Sens.)
98.0% (95% CI 93.0-99.8%)
NPA (Spec.)
100% (95% CI 91.4-100.0%)
ER (ESR1) (log2 nRPM)
ER Neg. (0%)
ER Low (1-10%)
ER Pos. (>10%)
e
qTP PR vs. IHC (validation cohort)
PPA (Sens.)
77.2% (95% CI 65.3-86.7%)
NPA (Spec.)
100% (95% CI 95.5-100.0%)
PR (PGR) (log2 nRPM)
PR Neg. (0%)
PR Low (1-10%)
PR Pos. (>10%)
f
qTP HER2 (ERBB2) vs. IHC (validation cohort)
PPA (Sens.)
80.0% (95% CI 51.9-95.7%)
ERBB2 amp
ERBB2 wt
ERBB2 NA
NPA (Spec.)
100%
(95% CI 98.2-100.0%)
HER2 (ERBB2) (log2 nRPM)
HER2 IHC
g
4 month informative current CGqTP (n=3,904)
Breast cancer (n=288)
SOC PIK3CA mutation (n=90)
Excluded
Not breast cancer
Not meeting qTP or CGP QC metrics
(n=3,616)
Excluded
Not PIK3CA SOC mutated
(n=198)
qTP HR+ / ERBB2 wt (n=77)
qTP HR+ / ERBB2 amp (n=1)
qTP HR- / ERBB2 wt (n=11)
qTP HR- / ERBB2 amp (n=1)

Figure S18

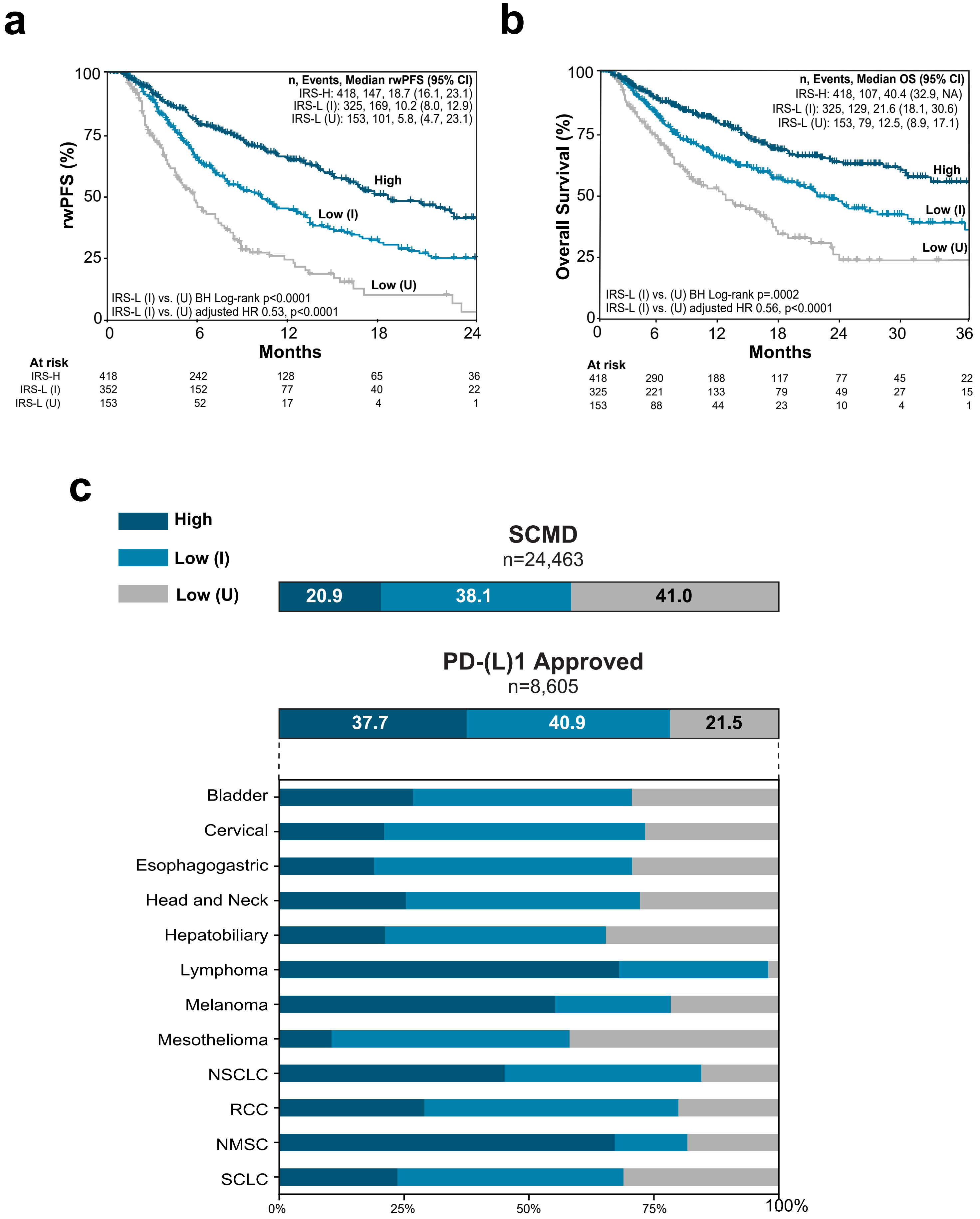
a
n, Events, Median rwPFS (95% CI)
IRS-H: 418, 147, 18.7 (16.1, 23.1)
IRS-L (I): 325, 169, 10.2 (8.0, 12.9)
IRS-L (U): 153, 101, 5.8, (4.7, 23.1)
rwPFS (%)
High
Low (I)
Low (U)
IRS-L (I) vs. (U) BH Log-rank p<0.0001
IRS-L (I) vs. (U) adjusted HR 0.53, p<0.0001
Months
At risk
IRS-H 418 242 128 65 36
IRS-L (I) 352 152 77 40 22
IRS-L (U) 153 52 17 4 1
b
n, Events, Median OS (95% CI)
IRS-H: 418, 107, 40.4 (32.9, NA)
IRS-L (I): 325, 129, 21.6 (18.1, 30.6)
IRS-L (U): 153, 79, 12.5, (8.9, 17.1)
Overall Survival (%)
High
Low (I)
Low (U)
IRS-L (I) vs. (U) BH Log-rank p=.0002
IRS-L (I) vs. (U) adjusted HR 0.56, p<0.0001
Months
At risk
418 290 188 117 77 45 22
325 221 133 79 49 27 15
153 88 44 23 10 4 1
c
High
Low (I)
Low (U)
SCMD
n=24,463
20.9
38.1
41.0
PD-(L)1 Approved
n=8,605
37.7
40.9
21.5
Bladder
Cervical
Esophagogastric
Head and Neck
Hepatobiliary
Lymphoma
Melanoma
Mesothelioma
NSCLC
RCC
NMSC
SCLC
0%
25%
50%
75%
100%

Figure S19

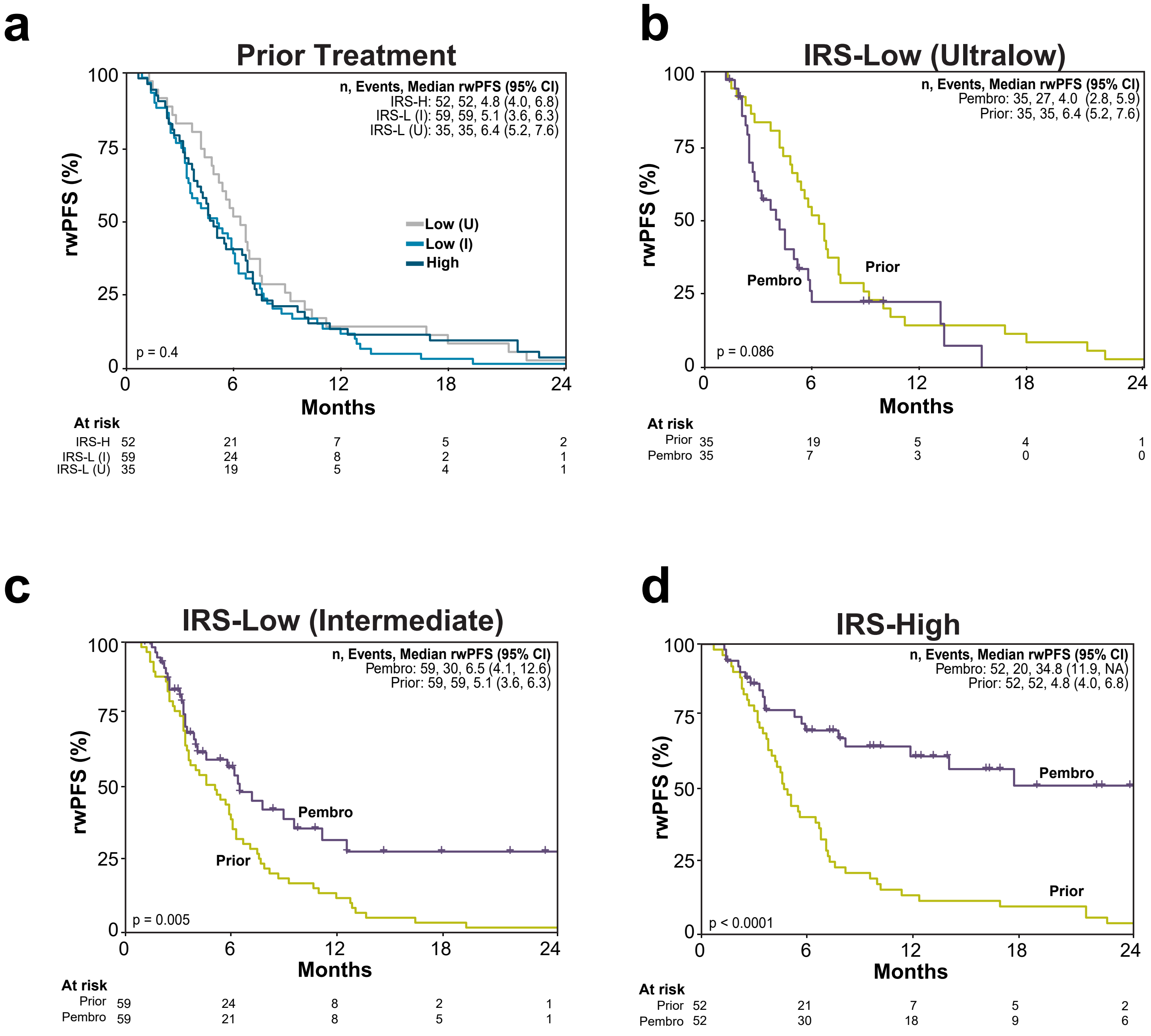
a
Prior Treatment
n, Events, Median rwPFS (95% CI)
IRS-H: 52, 52, 4.8 (4.0, 6.8)
IRS-L (I): 59, 59, 5.1 (3.6, 6.3)
IRS-L (U): 35, 35, 6.4 (5.2, 7.6)
Low (U)
Low (I)
High
p = 0.4
rwPFS (%)
100
75
50
25
0
0
6
12
18
24
Months
At risk
IRS-H 52 21 7 5 2
IRS-L (I) 59 24 8 2 1
IRS-L (U) 35 19 5 4 1
b
IRS-Low (Ultralow)
n, Events, Median rwPFS (95% CI)
Pembro: 35, 27, 4.0 (2.8, 5.9)
Prior: 35, 35, 6.4 (5.2, 7.6)
Prior
Pembro
p = 0.086
rwPFS (%)
Months
At risk
Prior 35 19 5 4 1
Pembro 35 7 3 0 0
c
IRS-Low (Intermediate)
n, Events, Median rwPFS (95% CI)
Pembro: 59, 30, 6.5 (4.1, 12.6)
Prior: 59, 59, 5.1 (3.6, 6.3)
Pembro
Prior
p = 0.005
rwPFS (%)
Months
At risk
Prior 59 24 8 2 1
Pembro 59 21 8 5 1
d
IRS-High
n, Events, Median rwPFS (95% CI)
Pembro: 52, 20, 34.8 (11.9, NA)
Prior: 52, 52, 4.8 (4.0, 6.8)
Pembro
Prior
p < 0.0001
rwPFS (%)
Months
At risk
Prior 52 21 7 5 2
Pembro 52 30 18 9 6

SUPPLMENTARY TABLES

Table S1. Univariate and multivariate associations of comprehensive genomic and quantitative transcriptomic profiling derived biomarkers and pembrolizumab real-world progression-free survival

Biomarker	Univariate		Multivariate (IRS model)	
	HR (95% CI)	p-value	HR	p-value
**TMB**	0.79 (0.73 - 0.86)	1.6E-08	0.76 (0.7 - 0.83)	7.1E-11
**_(PD-1) PDCD1_ Amplicon 1**	0.91 (0.86 - 0.96)	0.001	0.89 (0.84 - 0.95)	0.001
**_(PD-L1) CD274_ Amplicon 1**	0.92 (0.87 - 0.98)	0.005	0.94 (0.88 - 1)	0.048
_(PD-L1) CD274_ Amplicon 2	0.92 (0.87 - 0.98)	0.005		
IFNG**	0.97 (0.94 - 1)	0.028		
(PD-1) PDCD1 Amplicon 2	0.94 (0.9 - 0.99)	0.029		
_CD8A_	0.95 (0.9 - 1)	0.038		
TIGIT	0.95 (0.9 - 1)	0.054		
TNFRSF9	0.95 (0.9 - 1.01)	0.086		
IDO1	0.97 (0.93 - 1.01)	0.096		
PDCD1LG2	0.95 (0.89 - 1.01)	0.102		
GZMA	0.96 (0.91 - 1.01)	0.126		
LAG3	0.95 (0.89 - 1.02)	0.155		
UBE2C*	0.95 (0.88 - 1.03)	0.225		
**_ADAM12_**	1.03 (0.97 - 1.1)	0.276	1.06 (1 - 1.13)	0.055
TCF7	0.97 (0.9 - 1.05)	0.486		
_CD4_	1.02 (0.94 - 1.12)	0.618		
VTCN1	0.99 (0.97 - 1.02)	0.639		
CTLA4	0.99 (0.94 - 1.04)	0.648		
HAVCR2	0.99 (0.92 - 1.06)	0.724		
AXL	1.01 (0.94 - 1.1)	0.725		
TNFRSF4	1.00 (0.94 - 1.07)	0.930		
**_TOP2A*_**	1.00 (0.92 - 1.09)	0.972	1.08 (0.99 - 1.18)	0.080
FOXP3	1.00 (0.94 - 1.06)	0.988		

Derived candidate biomarkers and real-world progression-free survival in pembrolizumab treated patients (n=648). For each biomarker amplicon, the hazard ratio (HR; with 95% confidence interval [CI]) and log-likelihood p-value are shown. TMB (log_2) was from StrataNGS CGP testing; the remaining biomarkers were target gene expression from in-parallel quantitative transcriptomic profiling (qTP). The multivariate analysis was performed using the final five component Immunotherapy Response Score (IRS) model (indicated by bolded text). *Candidate proliferation markers. **Limit of quantification (LOQ) could not be established for this gene; therefore, it was not used for subsequent analysis.

Table S2. Pan-cancer correlation of candidate IRS target gene expression between Strata multiplex PCR-based NGS and TCGA

Gene	Type	Target gene expression Strata (n=18,305) vs. TCGA (n= 9,223)	
		**ρ**	**p-value**
TOP2A	IRS component (Proliferation)	0.896	9.71E-17
CD274 (PD-L1)	IRS component (Immune candidate)	0.831	1.62E-12
PDCD1 (PD-1)	IRS component (Immune candidate)	0.815	9.22E-12
ADAM12	IRS component (Immune candidate)	0.707	5.63E-08
	**Subset Median:**	**0.823**	
VTCN1	Immune candidate	0.938	1.92E-21
IDO1	Immune candidate	0.915	1.44E-18
UBE2C	Proliferation	0.887	4.67E-16
TNFRSF9	Immune candidate	0.869	9.41E-15
CTLA4	Immune candidate	0.869	1.02E-14
HAVCR2	Immune candidate	0.845	2.79E-13
LAG3	Immune candidate	0.833	1.28E-12
GZMA	Immune candidate	0.831	1.56E-12
TIGIT	Immune candidate	0.809	1.82E-11
TCF7	Immune candidate	0.778	3.26E-10
TNFRSF4	Immune candidate	0.761	1.28E-09
FOXP3	Immune candidate	0.749	3.17E-09
CD8A	Immune candidate	0.749	3.20E-09
CD4	Immune candidate	0.708	5.27E-08
AXL	Immune candidate	0.679	3.02E-07
PDCD1LG2	Immune candidate	0.624	4.72E-06
	**Overall Median:**	**0.831**	

The correlation of expression profiles of 20 candidate genes between 9,223 TCGA tumors and 18,305 quantitative transcriptomic profiling (qTP) assessed tumors (of 24,463 samples, limited to 27 directly comparable tumor types) was determined. The number of samples used in comparison (n), the correlation (Spearman rho, *P*) and the significance with respect to no correlation (p-value, reported in scientific notation). TCGA data was obtained from cBioPortal. Components of the final Immunotherapy Response Score (IRS) model are bolded. For PD-L1 (*CD274*) and PD-1 (*PDCD1*), two independent target amplicons were assessed for each gene; normalized target gene expression was averaged from the independent amplicons (per gene) to yield a composite result. TCGA= the Cancer Genome Atlas. As limit of quantification (LOQ) could not be established for *IFNG* by Strata Multiplex PCR based-NGS profiling, it was excluded from this analysis.

Table S3. Correlation of individual components of the IRS model

	PD-L1 (CD274)	***PD-1 (PDCD1)***	***ADAM12***	***TOP2A***	**TMB**
PD-L1 (CD274)		0.571	0.251	0.049	0.134
PD-1 (PDCD1)	0.571		0.219	0.033	0.102
ADAM12	0.251	0.219		0.06	0.032
TOP2A	0.049	0.033	0.06		0.211
**TMB**	0.134	0.102	0.032	0.211	

The Spearman correlation (ρ) of individual components of IRS, including TMB (log2) and the four individual gene expression biomarkers (*PD-L1*, *PD-1*, *ADAM12*, and *TOP2A*) was determined across 24,463 samples with informative IRS within the cohort used to evaluated IRS distribution across tumors submitted for comprehensive genomic profiling. TMB= tumor mutation burden, IRS= Immunotherapy response score.

Table S4. Real-world PFS (rwPFS) and overall survival (OS) benefit after unadjusted and adjusted restricted mean survival time analysis

Analysis	Endpoint	Restricted Time (tau)	Variable	Estimate (95% CI)	p-value
Unadjusted RMST	rwPFS	24 Months	IRS-H	15.70 (14.53 - 16.88)	<0.0001*
			IRS-L	10.63 (9.61 - 11.65)	
	OS	36 Months	IRS-H	25.50 (23.61 - 27.39)	<0.0001*
			IRS-L	19.24 (17.48 - 21.00)	
Adjusted RMST	rwPFS	24 Months	Intercept	10.63 (5.27 - 15.99)	0.0001
			IRS (H vs. L)	4.80 (3.20 - 6.41)	<0.0001
			Age	0.02 (-0.05 - 0.09)	0.5984
			Gender (M vs. F)	0.24 (-1.31 - 1.79)	0.7662
			Tumor Type (NSCLC vs. Other)	0.03 (-1.74 - 1.80)	0.9748
			Line of Therapy	-1.22 (-2.32 - -0.12)	0.0298
			Type of Therapy (Mono vs. Combo)	0.48 (-1.26 - 2.22)	0.5891
	OS	36 Months	Intercept	27.95 (18.80 - 37.10)	<0.0001
			IRS (H vs. L)	6.00 (3.37 - 8.63)	<0.0001
			Age	-0.10 (-0.22 - 0.02)	0.0920
			Gender (M vs. F)	-0.02 (-2.62 - 2.59)	0.9897
			Tumor Type (NSCLC vs. Other)	0.12 (-2.92 - 3.17)	0.9382
			Line of Therapy	-1.66 (-3.53 - 0.21)	0.0825
			Type of Therapy (Mono vs. Combo)	0.91 (-2.08 - 3.89)	0.5524

rwPFS= real-world progression-free survival, OS= overall survival, CI= confidence interval, IRS= immunotherapy response score, H= high, L= low, vs.= versus, RMST= restricted mean survival time, M= male, F= female, NSCLC= Non-Small Cell Lung Cancer, mono= monotherapy, combo= combination therapy. *p-value is for a test of RMST equality between IRS-H and IRS-L

Table S5. Dependency of rwPFS from immediately prior therapy to pembrolizumab monotherapy after adjustment for various covariates

	**Reduced Model**		**Full Model**	
**Covariate**	**HR (05% CI)**	**p-value**	**HR (95% CI)**	**p-value**
IRS group (IRS-high vs. IRS-low)	0.69 (0.52 – 0.93)	0.015	0.98 (0.70 - 1.38)	0.92
Age	1.00 (0.99 - 1.02)	0.7	1.00 (0.99 - 1.02)	0.81
Gender (Male vs. Female)	0.90 (0.70 - 1.16)	0.397	0.90 (0.70 - 1.17)	0.43
Tumor Type (NSCLC vs. Other)	1.10 (0.72 - 1.69)	0.659	1.09 (0.69 - 1.70)	0.72
Line of Therapy	1.06 (0.85 - 1.33)	0.59	1.05 (0.82 - 1.34)	0.68
Type of Therapy (pembro monotherapy vs. prior therapy)	0.53 (0.36 - 0.76)	0.001	0.74 (0.50 - 1.10)	0.14
Type of Therapy x IRS Group	NA	NA	0.37 (0.19 - 0.69)	0.002
**Likelihood ratio test for interaction p = 0.001**				

rwPFS= real-world progression-free survival, HR = hazard ratio, CI= confidence interval, IRS= immunotherapy response score, NSCLC= Non-Small Cell Lung Cancer

Table S6. Pre-/post-propensity score matching covariates in first-line NSCLC cohort treated with pembrolizumab monotherapy or pembrolizumab + chemotherapy combination therapy

Matching Method	Variable	Statistic	Pembro + Chemo	Pembro Mono	p-value *
**None**		n	133	109	NA
	Age	Mean	66.5	71.9	<0.0001
	TMB	Mean	2.8	3	0.293
	CD274	Mean	10.63	11.82	<0.0001
	Gender (Female)	n (%)	60 (45.1%)	54 (49.5%)	0.5193
	IRS (High)	n (%)	69 (51.9%)	61 (56%)	0.6044
**NN – 0.25*sd caliper**		n	77	77	NA
	Age	Mean	68.94	69.97	0.5258
	TMB	Mean	3.01	2.84	0.375
	CD274	Mean	11.23	11.42	0.4596
	Gender (Female)	n (%)	41 (53.2%)	35 (45.5%)	0.4204
	IRS (High)	n (%)	46 (59.7%)	40 (51.9%)	0.4172

NN = nearest neighbor, sd = standard deviation, n = sample size, % = percent, NA = not applicable, IRS = immunotherapy response score, TMB = tumor mutation burden. * p-value is based on a t-test for difference in means for continuous variables and a Fisher's exact test for categorical variables

Table S7. Impact of the tumor type term on the adjusted hazard ratio (HR) of IRS in the Cox proportional hazards model in the discovery and validation cohorts

Cohort	n	Analysis	Most Common vs. Other Tumor Type		MSKCC Sensitive vs. Insensitive Tumors	
			IRS (-H vs. -L) Adjusted HR (95% CI)	p-value	IRS (-H vs. -L) Adjusted HR (95% CI)	p-value
Discovery	648	rwPFS	0.49 (0.39-0.63)	<0.0001	0.50 (0.39-0.64)	<0.0001
Discovery	648	OS	0.53 (0.40-0.70)	<0.0001	0.54 (0.41-0.71)	<0.0001
Validation	248	rwPFS	0.52 (0.34-0.80)	0.003	0.46 (0.30-0.71)	0.0004
Validation	248	OS	0.49 (0.30-0.80)	0.005	0.43 (0.26-0.73)	0.001

The primary analyses presented herein used most common vs. other (e.g. NSCLC vs. all other tumor types in the discovery cohort) as the tumor type term in the Cox proportional hazards model used to evaluate the predictive ability of IRS for rwPFS and OS in the discovery (monotherapy and combination therapy) and validation cohorts. The impact on the adjusted HR (for IRS-High [H] vs. IRS-Low [L]) and p-value in these models was determined after replacing that tumor type term with the MSKCC definition of TMB sensitive tumor types (MSI-H, $\textit{POLE}^{\text{mutant}}$, NSCLC, head and neck cancer, or melanoma as TMB sensitive; all other samples as TMB insensitive)[63].
