# Supplementary material for: Development and validation of an integrative pan-solid tumor predictor of PD-1/PD-L1 blockade benefit
Source: Commun Med (Lond). 2023 Feb 7;3:14. doi: 10.1038/s43856-023-00243-7 (PMC9905474; doi:10.1038/s43856-023-00243-7)
Supplement: Supplementary file 8 — Reporting Summary [file 43856_2023_243_MOESM8_ESM.pdf]

nature portfolio

Corresponding author(s): Scott Tomlins and Daniel Rhodes

Last updated by author(s): Jan 9, 2023

Reporting Summary

Nature Portfolio wishes to improve the reproducibility of the work that we publish. This form provides structure for consistency and transparency in reporting. For further information on Nature Portfolio policies, see our Editorial Policies and the Editorial Policy Checklist.

Statistics

For all statistical analyses, confirm that the following items are present in the figure legend, table legend, main text, or Methods section.

n/a	Confirmed	
☐	☒	The exact sample size (*n*) for each experimental group/condition, given as a discrete number and unit of measurement
☐	☒	A statement on whether measurements were taken from distinct samples or whether the same sample was measured repeatedly
☐	☒	The statistical test(s) used AND whether they are one- or two-sided<br>*Only common tests should be described solely by name; describe more complex techniques in the Methods section.*
☐	☒	A description of all covariates tested
☐	☒	A description of any assumptions or corrections, such as tests of normality and adjustment for multiple comparisons
☐	☒	A full description of the statistical parameters including central tendency (e.g. means) or other basic estimates (e.g. regression coefficient) AND variation (e.g. standard deviation) or associated estimates of uncertainty (e.g. confidence intervals)
☐	☒	For null hypothesis testing, the test statistic (e.g. *F*, *t*, *r*) with confidence intervals, effect sizes, degrees of freedom and *P* value noted<br>*Give P values as exact values whenever suitable.*
☒	☐	For Bayesian analysis, information on the choice of priors and Markov chain Monte Carlo settings
☐	☒	For hierarchical and complex designs, identification of the appropriate level for tests and full reporting of outcomes
☐	☒	Estimates of effect sizes (e.g. Cohen's *d*, Pearson's *r*), indicating how they were calculated

Our web collection on statistics for biologists contains articles on many of the points above.

Software and code

Policy information about availability of computer code

Data collection	No code was in the collection of data for this study.
Data analysis	The IRS model algorithm is available through GitHub:<br>https://github.com/StrataOncology/immune-response-score<br>https://doi.org/10.5281/zenodo.7510725

For manuscripts utilizing custom algorithms or software that are central to the research but not yet described in published literature, software must be made available to editors and reviewers. We strongly encourage code deposition in a community repository (e.g. GitHub). See the Nature Portfolio guidelines for submitting code & software for further information.

Data

Policy information about availability of data

All manuscripts must include a data availability statement. This statement should provide the following information, where applicable:

- Accession codes, unique identifiers, or web links for publicly available datasets
- A description of any restrictions on data availability
- For clinical datasets or third party data, please ensure that the statement adheres to our policy

Due to applicable data sharing agreements and/or patient informed consent forms with Strata Trial health care systems and participants, the authors are restricted

from making raw patient-level genomic sequencing data publicly available or deposited. Interested parties may contact the authors at BD@strataoncology.com to request access for research purposes, and such requests will be handled on a case-by-case basis. All clinical and treatment data for the discovery and validation cohorts described herein (including the raw TMB and expression biomarker data used to derive the IRS algorithm output) are available in Supplementary Data 4; this file also provides source data for all figures on separate tabs. All other data are available from the corresponding author on reasonable request.

Human research participants

Policy information about studies involving human research participants and Sex and Gender in Research.

Reporting on sex and gender	Study findings are not restricted to only one gender and the study populations included both females and males. Gender was based on what was self-reported. For the IRS Distribution Cohort (n=24,463), there were 12,948 (52.9%) females. For the Systemic Therapy cohort (n=9,899), there were 5,317 (53.7%) females. Additional information regarding gender with respect to other cohorts are found in Supplement. To evaluate the potential of the multivariate model to predict PD-1/PD-L1 blockade treatment outcome, Immunotherapy Response Scores (IRS) group outcomes were compared by Kaplan Meier analysis and Cox proportional hazards modeling after adjusting for age, gender, most frequent tumor type (NSCLC) vs. others, line type (monotherapy/combination therapy) and line of systemic therapy. Propensity score matching was used to identify matched cohorts of patients with NSCLC treated with 1st line systemic pembrolizumab (pembro) monotherapy (n=77) or pembrolizumab + chemotherapy (chemo) combination therapy (n=77) that did not significantly differ in age, gender, TMB status, PD-L1 expression by quantitative transcriptomic profiling (qTP; the expression biomarker component of IRS), or IRS status.
Population characteristics	With a data-cutoff of 12 July 2022, the SCMD contains clinical and/or molecular data from 57,648 unique patients with advanced solid tumors (from 47 tumor types) enrolled from 59 United States health care systems who had routine FFPE tumor tissue molecularly profiled by the StrataNGS CGP test, with 9,899 patients (from 43 tumor types and 30 United States health care systems) having treatment data from at least one antineoplastic agent. Among the 9,899 patients, the median follow-up from start of first systemic treatment was 15.4 months [interquartile range (IQR) 6.9-29.4 months]. The median number of total systemic lines of therapy per patient was 1 (IQR 1-2), with 49.2% of systemic lines being monotherapy, and the median number of systemic therapies per line was 2 (IQR 1-2). The median number of total systemic lines of therapy per patient after Strata trial enrollment was 1 (IQR 0-1), with 47.2% of systemic lines being monotherapy, and the median number of systemic therapies per line was 2 (IQR 1-2). Additional population characteristics including gender, age, tumor type, ethnicity, etc. are extensively detailed in the Supplemental Materials.
Recruitment	At enrolling health care systems (59 study locations), all adult patients with unresectable or metastatic solid tumors and available formalin-fixed paraffin-embedded (FFPE) tumor tissue were eligible to participate in The Strata Trial.
Ethics oversight	The Strata Trial has been reviewed and approved by Advarra Institutional Review Board (IRB; IRB Pro00019183) prior to study start.

Note that full information on the approval of the study protocol must also be provided in the manuscript.

Field-specific reporting

Please select the one below that is the best fit for your research. If you are not sure, read the appropriate sections before making your selection.

☒ Life sciences ☐ Behavioural & social sciences ☐ Ecological, evolutionary & environmental sciences

For a reference copy of the document with all sections, see nature.com/documents/nr-reporting-summary-flat.pdf

Life sciences study design

All studies must disclose on these points even when the disclosure is negative.

Sample size	Post-hoc power analysis was not performed to determine the sample size of this discovery cohort. A power analysis was then performed to determine the cohort size needed for an independent validation cohort. In the overall discovery cohort, 46% patients were IRS-H, and we observed an adjusted hazard ratio for IRS-H vs. IRS-L rwPFS of 0.49 (47% event rate); therefore, assuming an IRS-H to IRS-L ratio of 1:1 and a 50% event rate, a validation cohort of 180 patients would have 90% power to detect a similar (0.5) hazard ratio. We then identified all (n=248) patients in the SCMD meeting the above-described validation cohort inclusion/exclusion criteria (the same as the discovery cohort except only including any non-pembrolizumab PD-(L)1 monotherapy treatment); the locked IRS model (and -H vs. -L threshold) was then applied to these subjects.
Data exclusions	Exclusion and inclusion criteria for the discovery and validation cohorts are described in the Methods. A study diagram showing all excluded patients for each analysis is provided as Figure S1.
Replication	The IRS model was trained and validated in independent cohorts of patients. Reproducibility and analytical validation of the multiplex PCR-based quantitative transcriptomic profiling (qTP) and the integrated IRS model were performed and described in the Supplementary Results and Figure S8.
Randomization	Randomization was not performed as part of IRS development or validation given the observational nature of the clinical trial used to collect treatment data. For the clinical validation/clinical utility analysis of qTP for ER, PR and HER2 status in breast cancer (Figure S17), the overall

	cohort of each biomarker was randomly split into training and validation cohorts for clinical validity/utility assessment prior to performing accuracy assessment in the overall cohort.
Blinding	Blinding was not performed as part of IRS development or validation given the observational nature of the clinical trial used to collect treatment data.

Reporting for specific materials, systems and methods

We require information from authors about some types of materials, experimental systems and methods used in many studies. Here, indicate whether each material, system or method listed is relevant to your study. If you are not sure if a list item applies to your research, read the appropriate section before selecting a response.

Materials & experimental systems

n/a	Involved in the study
☒	☐ Antibodies
☒	☐ Eukaryotic cell lines
☒	☐ Palaeontology and archaeology
☒	☐ Animals and other organisms
☐	☒ Clinical data
☒	☐ Dual use research of concern

Methods

n/a	Involved in the study
☒	☐ ChIP-seq
☒	☐ Flow cytometry
☒	☐ MRI-based neuroimaging

Clinical data

Policy information about clinical studies

All manuscripts should comply with the ICMJE guidelines for publication of clinical research and a completed CONSORT checklist must be included with all submissions.

Clinical trial registration	NCT03061305
Study protocol	Study Protocol is available by contacting the corresponding author.
Data collection	Deidentified patients tested by a version of StrataNGS assessing TMB with parallel gene expression testing data from 25 January 2017 to 12 July 2022 were potentially eligible for analysis using a data cutoff of 12 July 2022.
Outcomes	We assigned patients to one of two IRS groups (IRS-low and IRS-high) to compare patient outcomes, which included treatment data (rwPFS and OS) for evaluation of PD-1 or PD-L1 inhibitor monotherapy benefit. Real-world progression-free survival (rwPFS) was by time to next therapy (TTNT) defined as the time in months from the initiation of a therapy to the date of commencement of the next line of therapy (or date of death).
